# Supplementary material for: Photoinitiated Multicomponent Anti-Markovnikov Alkoxylation over Graphene Oxide
Source: Molecules. 2022 Jan 12;27(2):475. doi: 10.3390/molecules27020475 (PMC8777657; doi:10.3390/molecules27020475)

Supporting Information for

Photoinitiated Multicomponent anti-Markovnikov Alkoxylation over Graphene Oxide

Liang Nie,[a] Xiangjun Peng,*[b] Haiping He,[b] Jian Hu,[b] Zhiyang Yao,[b] Lin Zhou,[a] Ming Yang[a], Fan Li,[a] Qing Huang*[a]

[a] *Department of Chemistry and Chemical Engineering, Gannan Normal University, Ganzhou, Jiangxi 341000, P. R. China*

[b] *School of Pharmaceutical Science, Gannan Medical University, Ganzhou, Jiangxi 341000, P. R. China*

E-mail: liuliangxian1962@163.com

CONTENTS

1. General Methods……………...…………………………………………………….2

2. Experimental Procedures……………...…………………………..…………….3

3. Characterization of Graphene Oxide (GO)...6

4. Mechanism Studies..…………………...9

5. Control Experiments..13

6. The X-ray Crystal Structure of 4f..…………………..15

7. ^{1}H, ^{19}F, ^{13}C NMR, MP and MS Data of All products..16

8. References..…………………...26

9. Copies of ^{1}H NMR, ^{19}F NMR and ^{13}C NMR Spectra for All Compounds........27

1. General Methods

Unless otherwise specified, commercial reagents and solvents were used without further purification. Commercially available chemicals were purchased from Shanghai Haohong Scientific Co., Ltd. (Leyan) and used without any further purification. ^{1}H and ^{13}C NMR spectra were recorded on a Bruker spectrometers at 400 and 100 MHz, respectively. The chemical shifts were given in parts per million relative to $CDCl_3$ (7.26 ppm for ^{1}H) and $CDCl_3$ (77.0 ppm for ^{13}C. Peak multiplicities were reported as follows: s, singlet; d, doublet; t, triplet; m, multiplet; br. s, broad singlet and *J*, coupling constant (Hz). Mass spectra were recorded with Bruker Dalton Esquire 3000 plus LC-MS apparatus. Elemental analysis were carried out on a Perkin-Elmer 240B instrument. HRFABMS spectra were recorded on a FTMS apparatus. Silica gel (300-400 mesh) was used for flash column chromatography, eluting (unless otherwise stated) with an ethyl acetate/petroleum ether (PE) (60-90 ℃) mixture.

The graphene oxide sample was recorded with Zeiss Sigma 300. Fourier transform infrared spectroscopy of graphene oxide recorded on a Jasco ATR MIRacle spectrophotometer. Samples were scanned in the 400-4000cm^{-1} region with KBr pellet. X-ray photoelectron spectroscopy recorded with Thermo Fisher Scientific K-Alpha using Al K_{α} radiation ($\hbar\omega$ =1253.6 eV). The X-ray power was 125 W. The spectra were recorded in the constant analyzer energy (CAE) mode with analyzer pass energies of 50 eV for the high resolution spectra. Charging effects were corrected by energy calibration on Al level relative to 284.80 eV. Compound **4f** were collected by a diffractometer Rigaku Oxford Diffraction Supernova Dual Source, Cu at Zero equipped with an AtlasS2 CCD using Cu Kα radiation (1.54178 Å) by using a w scan mode.

2. Experimental Procedures

2.1 General Procedure of the Products 4

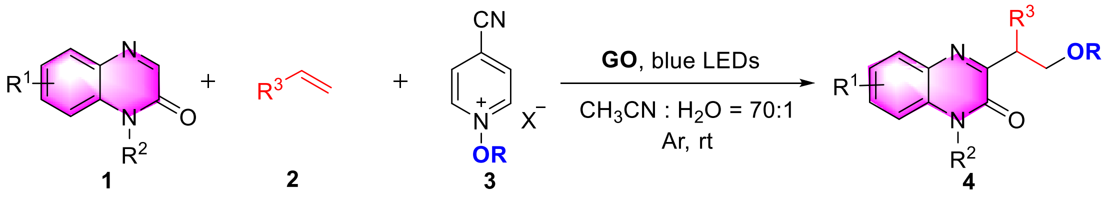

To a mixed solvent of MeCN and H_2O (70:1 v/v) of quinoxalone **1** (0.2 mmol), N-methoxypyridinium salt **3a** (0.6 mmol), and GO (80 wt%) was added styrene **2a** (0.3 mmol) under an argon atmosphere irradiated by 15w blue LED and the mixture was stirred at room temperature for 24 h. The reaction mixture was concentrated under reduced pressure. The residue was purified by flash chromatography on silica gel (eluent: EtOAc/PE = 1:4) to yield the corresponding product **4**.

2.2 General Procedure for the Synthesis of Quinoxalones 1

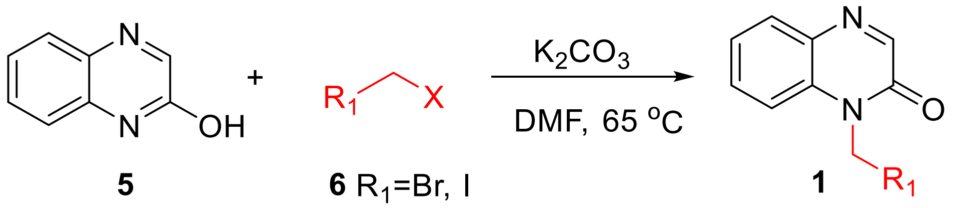

A 10 mL round-bottom flask was charged with quinoxalin-2-ol **5** (1 mmol), halogenated hydrocarbons **6** (1.6 equiv) and K_2CO_3 (1.2 equiv). Then anhydrous DMF (3 mL) was added to the flask and the resulting mixture was stirred at 65 °C for 12 h. Then, 30 mL of saturated NaCl (aq) was added to the mixture, which was extracted with ethyl acetate (5 mL × 5). The organic phase was dried over anhydrous $MgSO_4$ and filtered. The filtrate was concentrated under reduced pressure. The residue was purified by flash chromatography on silica gel (eluent: EtOAc/PE = 1:2) to yield the corresponding product **1**.[1]

2.3 Table S1. Optimization of the reaction conditions.[a]

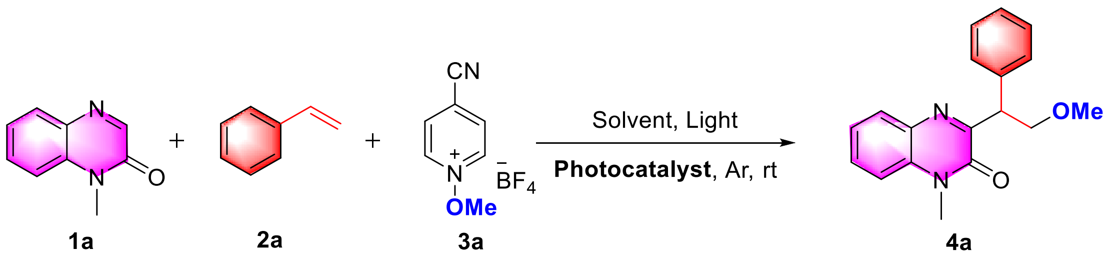

Entry	solvent	Light	Photocatalyst	Yield(%)[b]
1	Acetone	Blue	GO (100 wt%)	12%
2	EA	Blue	GO (100 wt%)	34%
3	DCE	Blue	GO (100 wt%)	NR

4	DMF	Blue	GO (100 wt%)	NR
5	MeOH	Blue	GO (100 wt%)	30%
6	THF	Blue	GO (100 wt%)	NR
7	DMSO	Blue	GO (100 wt%)	20%
8	H_2O	Blue	GO (100 wt%)	NR
9	CH_3CN	Blue	GO (100 wt%)	40%
10	dry CH_3CN	Blue	GO (100 wt%)	NR
11	CH_3CN:H_2O=200:1	Blue	GO (100 wt%)	50%
12	CH_3CN:H_2O=70:1	Blue	GO (100 wt%)	69%
13	CH_3CN:H_2O=40:1	Blue	GO (100 wt%)	45%
14	CH_3CN:H_2O=20:1	Blue	GO (100 wt%)	NR
15	CH_3CN:H_2O=70:1	Blue	Eosin Y (5 mol%)	25%
16	CH_3CN:H_2O=70:1	Blue	Fluorescein (5 mol%)	23%
17	CH_3CN:H_2O=70:1	Blue	Rose Bengal (5 mol%)	NR
18	CH_3CN:H_2O=70:1	Blue	GO (50 wt%)	58
**19**	**CH_3CN:H_2O=70:1**	**Blue**	**GO (80 wt%)**	**70%**
20	CH_3CN:H_2O=70:1	Blue	GO (200 wt%)	70%
21	CH_3CN:H_2O=70:1	Green	GO (80 wt%)	NR
22	CH_3CN:H_2O=70:1	White	GO (80 wt%)	NR
23[c]	CH_3CN:H_2O=70:1	Blue	GO (100 wt%)	62%
24[d]	CH_3CN:H_2O=70:1	Blue	GO (100 wt%)	NR
25[e]	CH_3CN:H_2O=70:1	Blue	GO (100 wt%)	trace
26	CH_3CN:H_2O=70:1	–	GO (80 wt%)	NR
27	CH_3CN:H_2O=70:1	Blue	–	NR

[a]Reaction conditions: **1a** (0.2 mmol, 1 equiv), **2a** (0.3 mmol, 1.5 equiv), **3a** (0.6 mmol, 3 equiv), 2.0 mL solvent under air atmosphere and irradiation of 15 W LEDs for 24 h at Ar and ambient temperature. [b]Isolated yields were showed. NR = no reaction. [c]**3a** (2 equiv). [d]O_2. [e]Air.

2.4 Table S2. Optimization kinds of various N-methoxypyridiniums[a]

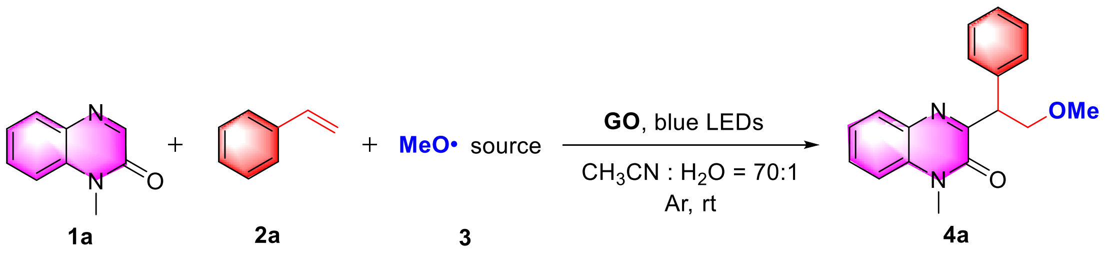

MeO• source

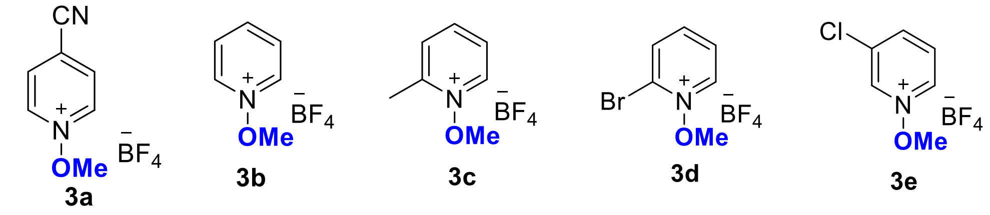

Yeild^{b} (%)	70	30	NR	NR	NR

^{a}Reaction conditions: **1** (0.2 mmol, 1 equiv), **2a** (0.3 mmol, 1.5 equiv), **3** (0.6 mmol, 3 equiv), GO (80 wt%), 15w blue LEDs, Ar, rt, and 24 h. ^{b}Isolated yield.

3. Characterization of Graphene Oxide (GO)

3.1 Infrared spectrum of GO

GO was prepared by graphite oxidation using the Hummers and Offeman method and subsequent exfoliation.[2] Considering the π-π interactions and oxygen-containing functional groups could facilitate the mass transfer and then increase catalytic performance, Fourier Transform infrared spectroscopy (FT-IR), scanning electron microscope (SEM) and X-ray photoelectron spectroscopy (XPS) were used to analyze and characterize this inorganic-organic hybrid material. As shown in Figure S1, the FT-IR spectrum of GO was observed the deformation vibration and stretching vibration of hydroxyl (O-H) at 3425 cm^{-1}, 2925 cm^{-1}, 2854 cm^{-1} and 2362 cm^{-1} respectively. The peak of 1626 cm^{-1} was stretching vibration absorption of carbonyl group (C=O), and the peak of 1399 cm^{-1} was stretching vibration of epoxy group (C-O). The wide peak at 1062 cm^{-1} came from the stretching vibration of alkoxy group (C-O) at the surface of GO.[3] The above FT-IR data indicated that GO surface was rich in hydroxyl, carbonyl, epoxide, ether and other oxygen-containing functional groups.

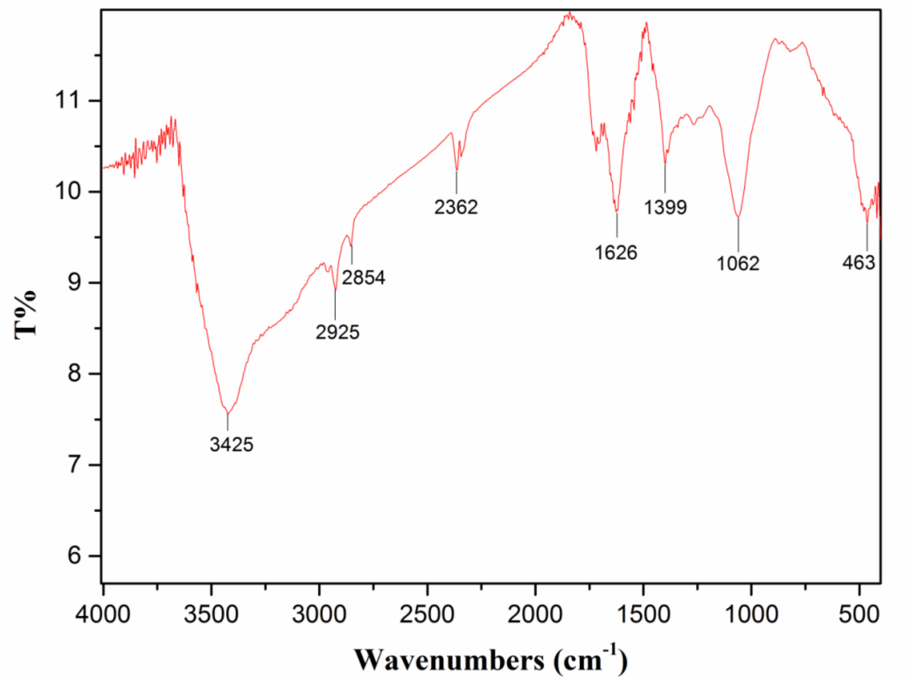

Figure S1. The infrared spectrum of GO

3.2 SEM of GO

The SEM image of the sample, a black flaky and blocky structure, was clearly observed that the GO has 1 μm in Figure S2. The surface of GO showed a classic folded morphology,[4] and its specific surface area was large, providing an ideal catalytic surface for the introduction of active centers.

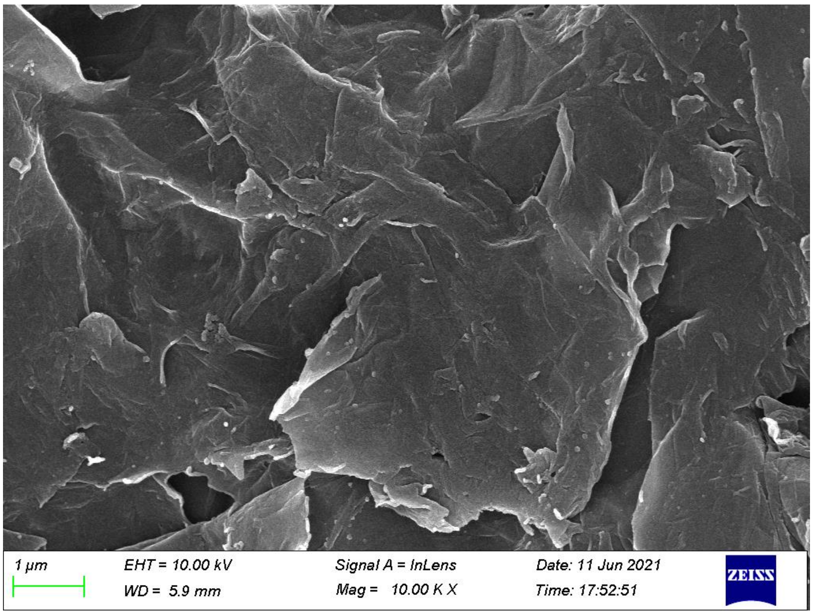

Figure S2. SEM image of GO.

3.3 XPS of GO

The samples were analyzed by XPS in order to study the surface chemical state and chemical composition of GO and GO-R. The full-scale XPS spectrum (Figure S3) proved that GO have C, O and S elements. For GO-R, the presence of C, O, S, F and B were confirmed by survey XPS spectra, indicating that the anion BF_4^- was successfully doped in carbon catalyst.

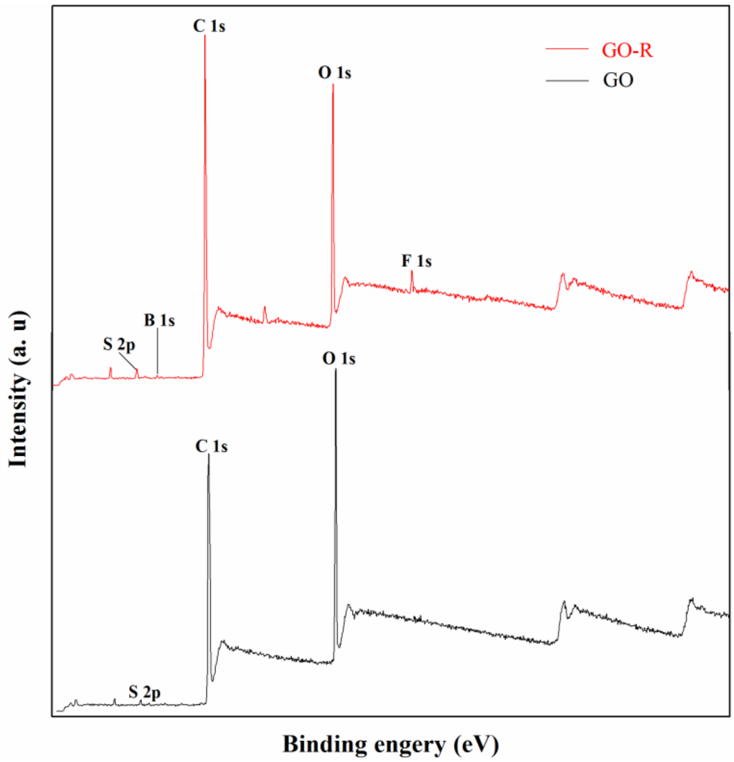

Figure S3. XPS image of GO.

The high-resolution spectra were given in Figure S4 to clarify the bond configurations of GO and GO-R. For both GO and GO-R, four characteristic peaks at about 284, 530, 680 and 190 eV appeared, which belonged to C 1s, O 1s, F 1s, and B 1s, respectively.

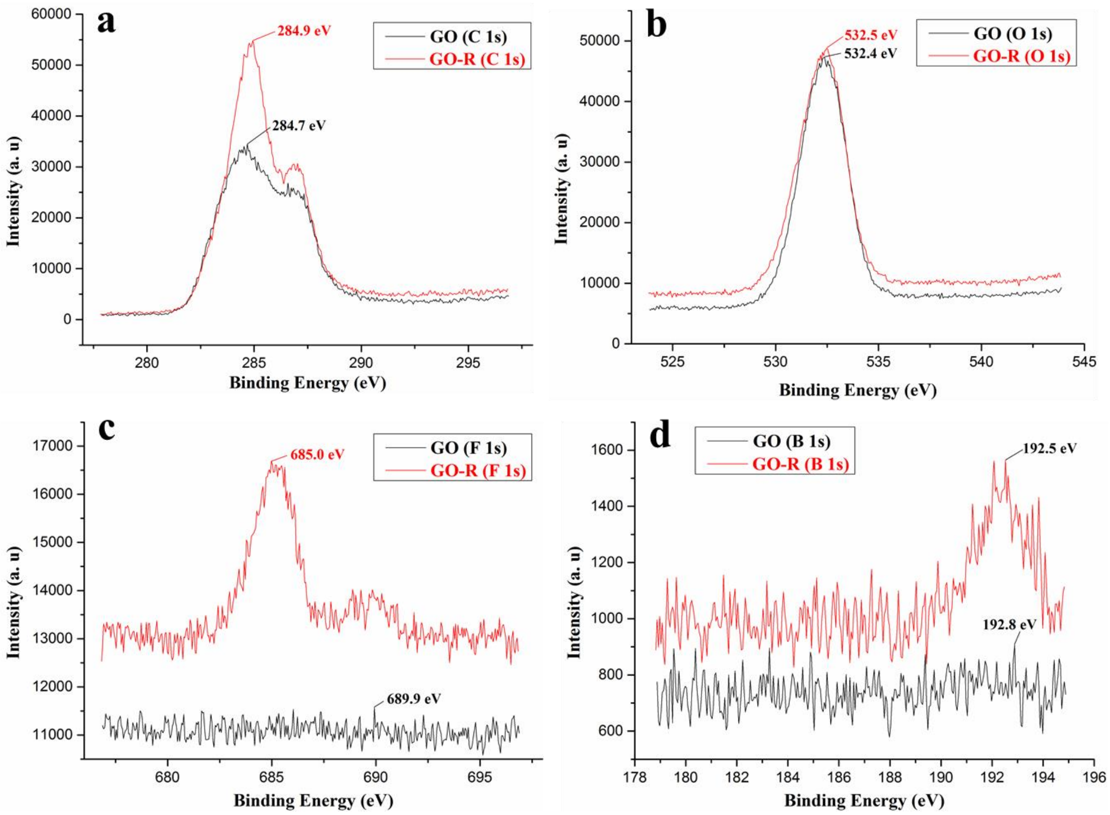

Figure S4. GO and GO-R XPS spectra of (a) C 1s, (b) O 1s, (c) F 1s and (d) B 1s.

XPS data also supported the conclusion that oxygen containing functional groups were slowly draining away in the recovered GO. As shown in Table S3, the percentage of C was increased substantially to 46.52% from 54.85%, while the O content was decreased to 33.98% from 47.63% suggesting that the oxygen containing functional groups of GO were consumed concomitantly to the reaction course, and aromatic carbons were not affected by the reaction. In addition, the F and B elements increased from 1.04% to 5.63% and 3.41% to 4.83%, respectively, while the S content was decreased to 0.7% from 1.4%.

Table S3. Summary of elemental analysis of GO and recovered GO catalyst by XPS measurement.

Catalyst	C%	O%	S%	F%	B%
GO	46.52	47.63	1.4	1.04	3.41
Recovered GO	54.85	33.98	0.7	5.63	4.83

4. Mechanism Studies

4.1. Ultraviolet–visible absorption of quinoxalone 1a, styrene 2a, 4-cyano-substituted N-methoxypyridinium 3a and GO

Ultraviolet–visible absorption experiments were performed using an Agilent Cary Eclipse UV-visible spectrophotometer. In each experiment, the varying samples were combined in CH_3CN/H_2O = 70/1 (v/v) in screw-top 1.0 cm quartz cuvettes. The UV−vis absorption experiments showed that GO possessed obvious absorption in the visible-light region (Figure S5). The UV-visible experiments were also performed to investigate whether GO with substrates to form electron donar-acceptor (EDA) complexes. After different substrates were added to 10^{-5} M solution of GO, the UV-visible spectra were recorded and no red-shift band was observed, indicating that no EDA ground state association occurred.

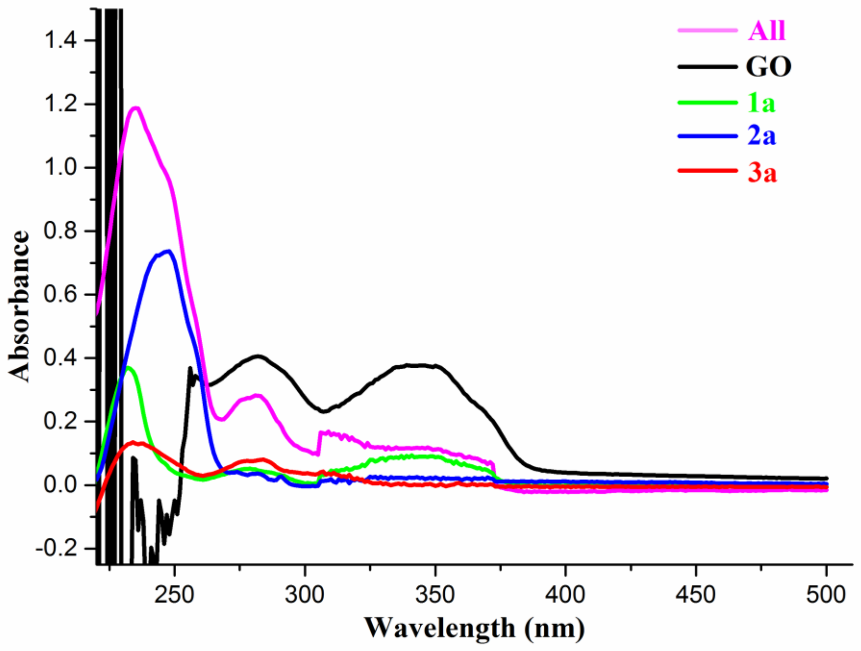

Figure S5. The UV-vis Absorption Experiments. The concentration of each component was 1.0 x 10^{-5} M.

4.2 The fluorescence spectrum of GO and substrates

We investigated the excitation and emission spectra of the GO. A solution of GO (1.0 mM) in a mixture of CH_3CN/H_2O in a 70:1 ratio was chosen as the model. The fluorescence excitation spectrum was obtained with the detection wavelength of 620 nm (Figure S6), and the fluorescence emission spectrum was excited at 419 nm (one excitation maximum of GO) (Figure S7). Then in the fluorescence quenching experiments, 1.0 mL of solution of GO (1.0 mM) in a mixture of CH_3CN/H_2O in a 70:1 ratio was added to the appropriate amount of quencher in a screw-top 1.0

cm quartz cuvette, 1 M solution of the quenchers (**1a, 2a, and 3a**) were added into the cuvette by 5 μL, and the emission of the sample was collected (Figure S8). The solution was excited at λ= 419 nm (excitation maximum of GO) and the emission intensity at 425 nm (emission maximum of GO) was observed (Figure S9). The fluorescence quenching experiments revealed that substrates effectively proceeded single-electron transfer (SET) between GO and substrates under visible-light irradiation.

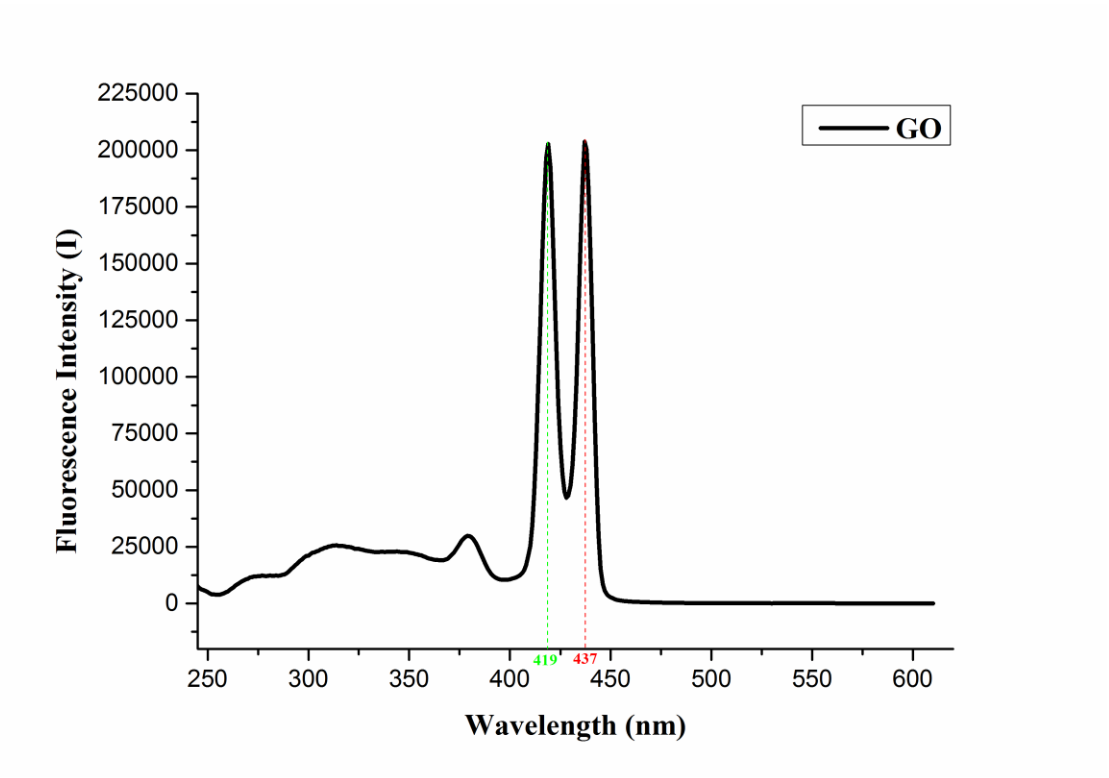

Figure S6. The fluorescence excitation spectrum of GO with the detection wavelength of 620 nm.

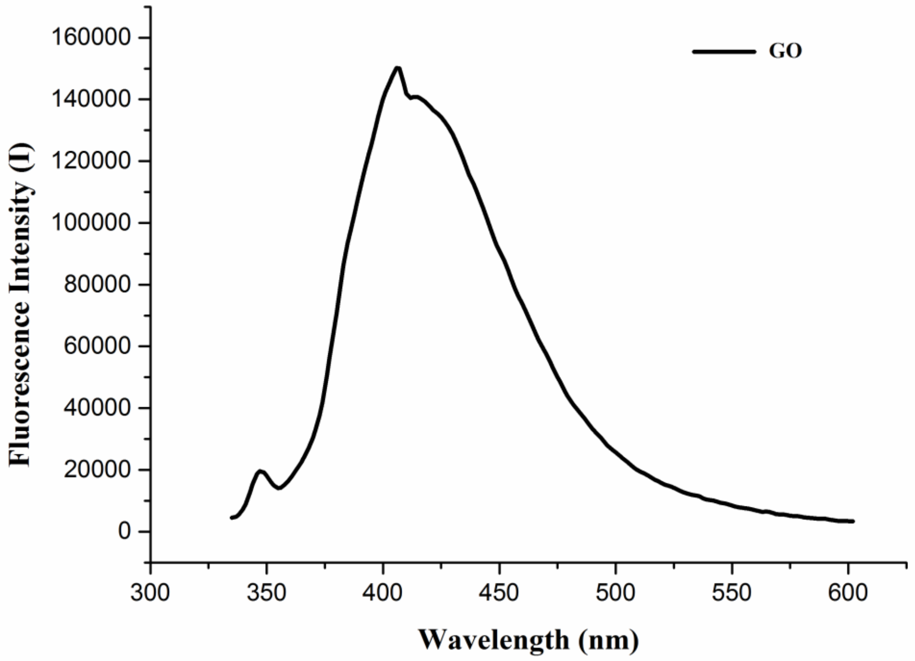

Figure S7. The fluorescence emission spectrum of GO excited at 419 nm.

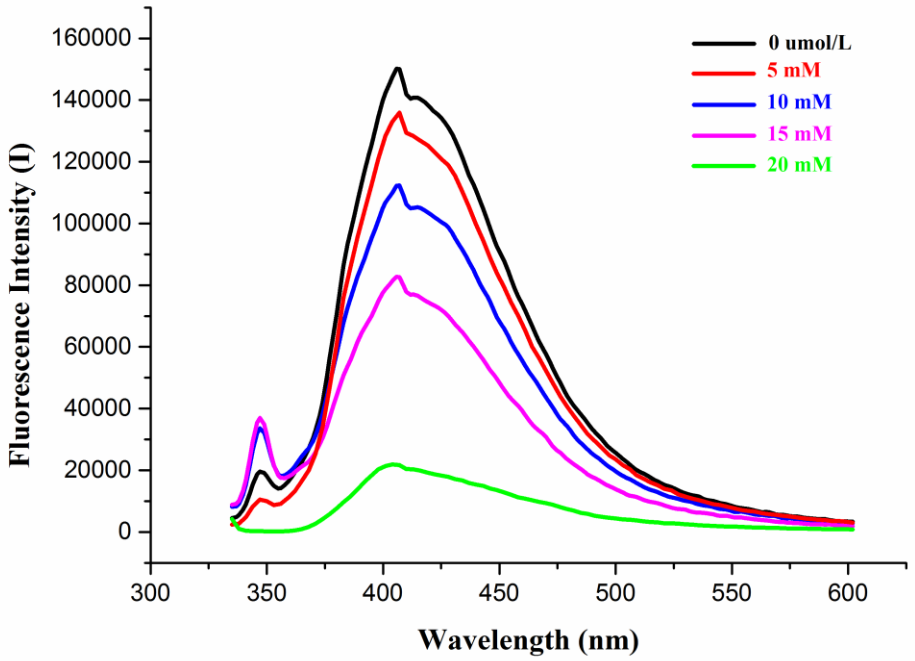

Figure S8. The fluorescence emission spectra of GO with different concentration of added quenchers excited at 419 nm.

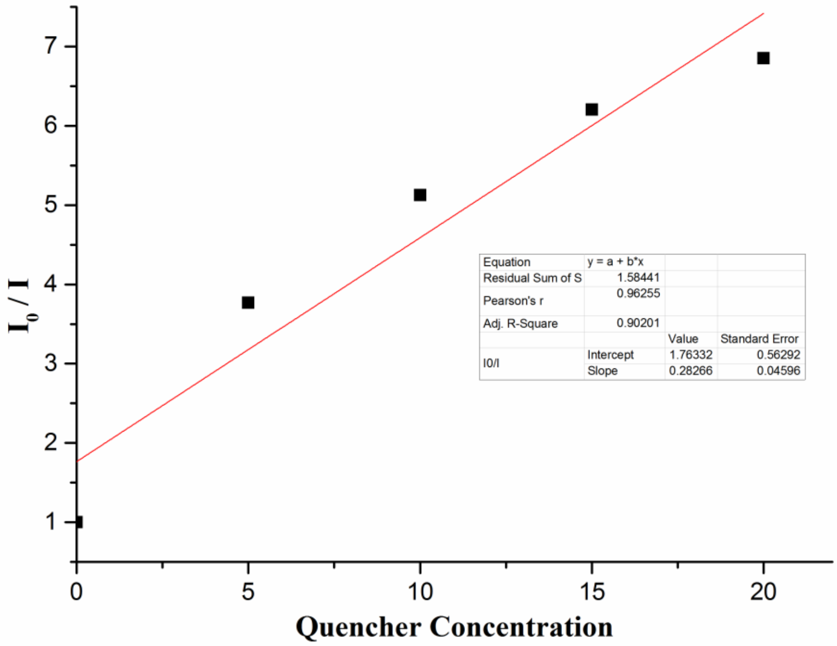

Figure S9. GO emission quenching by substrates linear quenching is observed.

5. Control Experiments

5.1 Control experiments from the anti-Markovnikov alkoxylation reaction

Some control experiments were performed to further investigate the mechanism of the anti-Markovnikov alkoxylation reaction (Figure S10). 2,2,6,6-Tetramethylpiperidinooxy (TEMPO) or 2,6-di-tert-butyl-4-methylphenol (BHT) was added to the reaction system, and the reaction was completely suppressed, which suggested the reaction could undergo a radical process.

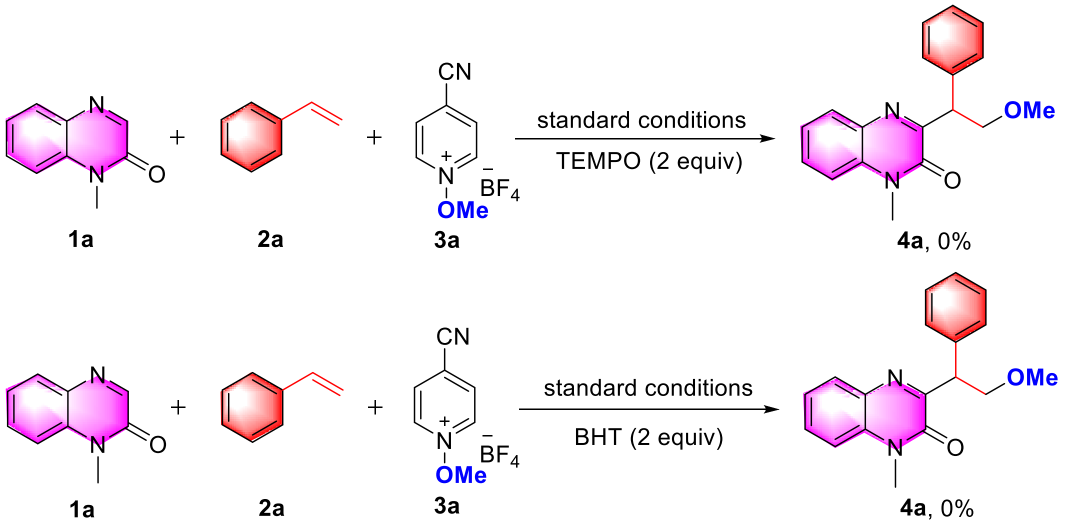

Figure S10. Control experiments.

5.2 Detection of intermediates from the reaction by ESI-HRMS

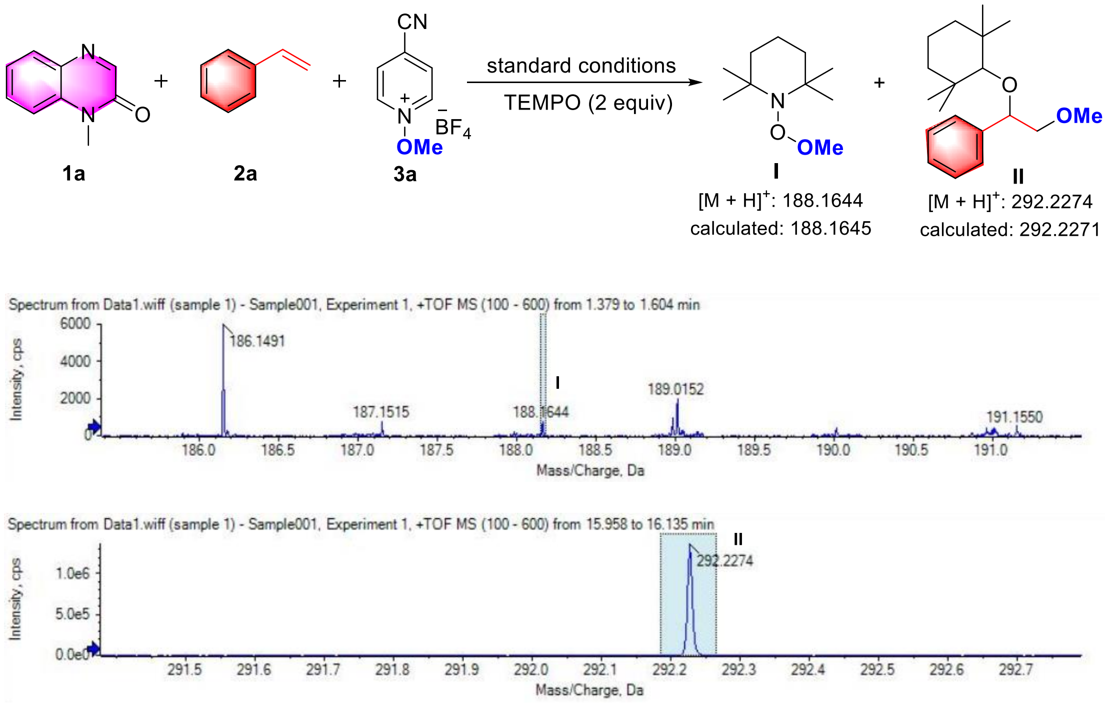

Figure S11. Reaction conditions: **1a** (0.2 mmol, 1 equiv), **2a** (0.3 mmol, 1.5 equiv), **3a** (0.6 mmol, 3 equiv), GO (80 wt%), 15w blue LEDs, Ar, rt, and 4 h.

5.3 Radical clock experiment

The cascade reaction of diethyl 2,2-diallylmalonate **5** as alkene with **1a** and **3a** furnished the cyclopentane derivative **III** in 18%, strongly supported that a radical mechanism was involved in this cascade reaction.

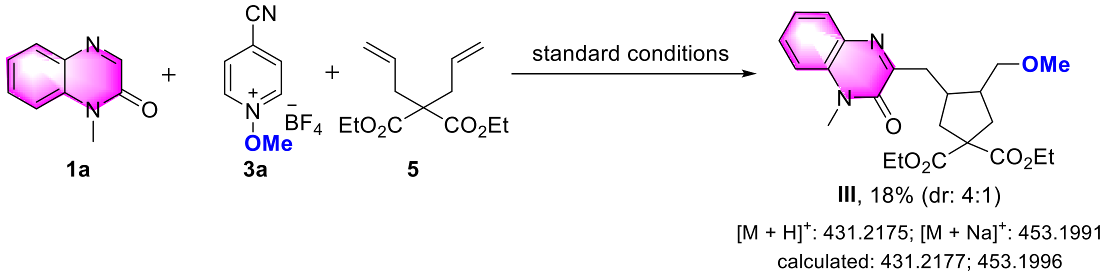

**Sample Name**	293-A	**Position**	P1-A7	**Instrument Name**	Instrument 1
**User Name**		**Inj Vol**	0.1	**InjPosition**	
**Sample Type**	Sample	**IRM Calibration Status**	Success	**Data Filename**	293-A.d
**ACQ Method**	yijing10-95% 8min Zheng.m	**Comment**		**Acquired Time**	7/15/2021 8:06:02 PM

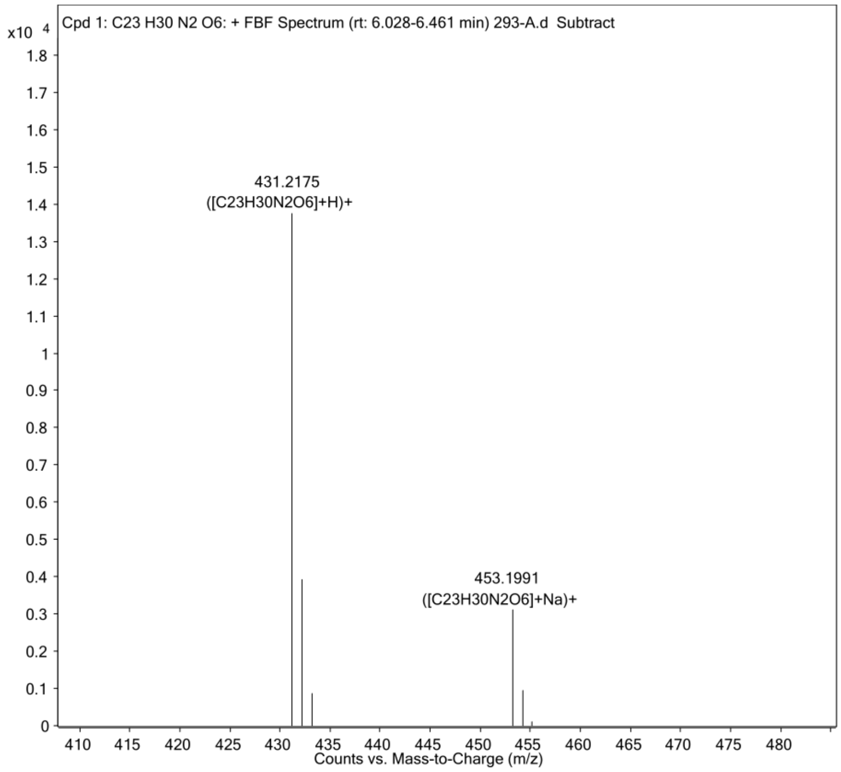

Figure S12. Radical clock experiment. Reaction conditions: **1a** (0.2 mmol, 1 equiv), **3a** (0.6 mmol, 3 equiv), **5** (0.4 mmol, 2 equiv), GO (80 wt%), 15w blue LEDs, Ar, rt, and 24 h.

6. The X-ray Crystal Structure of 4f

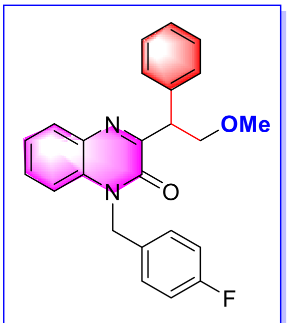

4f, CCDC: 2088403

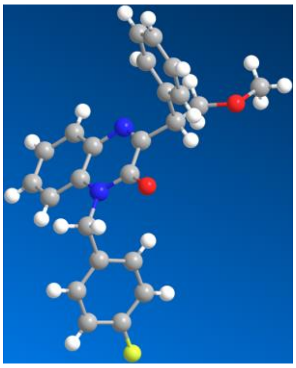

Table S4. Crystal data and structure refinement for complex 4f.

**Complex**	**4f**
Identification code	219A
Empirical formula	$C_{24}H_{21}FN_2O_2$
Formula weight	388.43
Temperature/K	149.99(10)
Crystal system	monoclinic
Space group	C2/c
a/Å	18.6213(18)
b/Å	11.8209(7)
c/Å	21.878(2)
α/°	90
β/°	123.235(13)
γ/°	90
Volume/Å^3	4028.0(8)
Z	8
ρ_{calc}g/cm^3	1.281
μ/mm^{-1}	0.088
F(000)	1632.0
Crystal size/mm^3	0.14 × 0.12 × 0.11
Radiation	Mo Kα (λ = 0.71073)
2Θ range for data collection/°	4.158 to 49.996
Index ranges	-19 ≤ h ≤ 22, -12 ≤ k ≤ 14, -26 ≤ l ≤ 26
Reflections collected	9151
Independent reflections	3543 [R_{int} = 0.0181, R_{sigma} = 0.0236]
Data/restraints/parameters	3543/0/263
Goodness-of-fit on F^2	1.047
Final R indexes [I>=2σ (I)]	R_1 = 0.0448, wR_2 = 0.1125
Final R indexes [all data]	R_1 = 0.0541, wR_2 = 0.1190
Largest diff. peak/hole / e Å^{-3}	0.18/-0.21

7. ^{1}H, ^{19}F, ^{13}C NMR, MP and MS Data of All products

3-(2-Methoxyl-1-phenylethyl)-1-methylquinoxalin-2(1*H*)-one (**4a**)

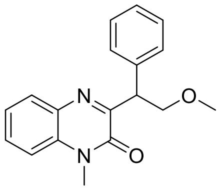

Yellow solid (41 mg, 70%). Mp: 98-101 ℃. ^{1}H NMR (400 MHz, $CDCl_3$): δ 7.96 (dd, J = 8.0, 1.2 Hz, 1H), 7.53 (dt, J = 1.2, 8.5 Hz, 1H), 7.50 (d, J = 8.5 Hz, 2H), 7.36 (t, J = 7.3 Hz, 1H), 7.31 (t, J = 7.3 Hz, 2H), 7.27 (d, J = 8.0 Hz, 1H), 7.23 (t, J = 7.3 Hz, 1H), 5.06 (dd, J = 9.0, 6.3 Hz, 1H), 4.41 (t, J = 9.0 Hz, 1H), 3.92 (dd, J = 9.0, 6.3 Hz, 1H), 3.64 (s, 3H), 3.41 (s, 3H). ^{13}C NMR (100 MHz, $CDCl_3$): δ 159.3, 154.6, 138.7, 133.1, 132.7, 130.3, 129.9, 128.7, 128.5, 127.1, 123.5, 113.5, 74.8, 59.0, 47.3, 29.1. HRESIMS calcd for $[C_{18}H_{18}N_2O_2 + H]^+$ 295.1447, found 295.1454.

3-(2-Methoxy-1-phenylethyl)-1,6,7-trimethylquinoxalin-2(1*H*)-one (**4b**)

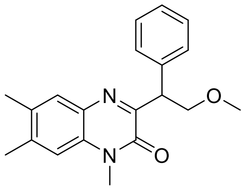

Yellow solid (37 mg, 58%), Mp: 129-132 ℃. ^{1}H NMR (400 MHz, $CDCl_3$): δ 7.71 (s, 1H), 7.49 (d, J = 7.4 Hz, 2H), 7.30 (t, J = 7.4 Hz, 2H), 7.21 (t, J = 7.4 Hz, 1H), 7.03 (s, 1H), 5.04 (t, J = 7.1 Hz, 1H), 4.37 (t, J = 8.9 Hz, 1H), 3.92 (t, J = 7.9 Hz, 1H), 3.61 (s, 3H), 3.40 (s, 3H), 2.42 (s, 3H), 2.38 (s, 3H). ^{13}C NMR (100 MHz, $CDCl_3$): δ 157.9, 154.6, 139.7, 139.0, 132.3, 131.1, 130.3, 128.7, 128.5, 128.4, 127.0, 114.1, 74.9, 59.0, 47.1, 29.1, 20.6, 19.2. HRESIMS calcd for $[C_{20}H_{22}N_2O_2 + H]^+$ 323.1760, found 323.1769.

6-Chloro-3-(2-methoxyl-1-phenylethyl)-1-methylquinoxalin-2(1*H*)-one (**4c**)

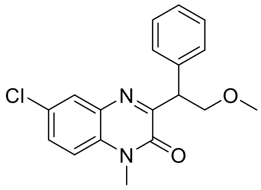

Yellow solid (36 mg, 55%). Mp: 127-130 ℃. ^{1}H NMR (400 MHz, $CDCl_3$): δ 7.96 (s, 1H), 7.48 (d, J = 8.5 Hz, 2H), 7.47 (s, 1H), 7.31 (t, J = 7.0 Hz, 2H), 7.24 (d, J = 7.0 Hz, 1H), 7.19 (d, J = 8.5 Hz, 1H), 5.07 (t, J = 6.7 Hz, 1H), 4.38 (t, J = 9.1 Hz, 1H), 3.87 (dd, J = 6.7, 1.8 Hz, 1H), 3.61 (s, 3H), 3.40 (s, 3H). ^{13}C NMR (100 MHz, $CDCl_3$): δ 160.7, 154.2, 138.2, 133.2, 131.8, 129.9, 129.5, 128.8, 128.7, 128.6, 127.3, 114.7, 74.6, 59.0, 47.4, 29.3. HRESIMS calcd for $[C_{18}H_{17}ClN_2O_2 + H]^+$ 329.1057, found 329.1067.

1-Benzyl-3-(2-methoxy-1-phenylethyl)quinoxalin-2(1*H*)-one (**4d**)

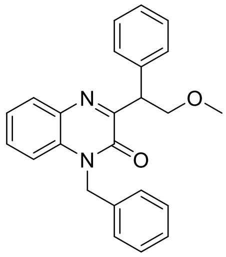

Yellow solid (48 mg, 65%). Mp: 85-87 ℃. ^{1}H NMR (400 MHz, $CDCl_3$): *δ* 7.99 (d, *J* = 7.8 Hz, 1H), 7.55 (d, *J* = 7.4 Hz, 2H), 7.41 (t, *J* = 7.7 Hz, 1H), 7.38-7.27 (m, 8H), 7.20 (d, *J* = 7.4 Hz, 2H), 5.54 (d, *J* = 15.6 Hz, 1H), 5.34 (d, *J* = 15.6 Hz, 1H), 5.17 (t, *J* = 7.0 Hz, 1H), 4.46 (t, *J* = 8.9 Hz, 1H), 3.98 (t, *J* = 8.3 Hz, 1H), 3.45 (s, 3H). ^{13}C NMR (100 MHz, $CDCl_3$): *δ* 159.4, 154.7, 138.7, 135.3, 133.0, 132.5, 130.4, 129.9, 128.9, 128.8, 128.6, 127.6, 127.2, 126.9, 123.6, 114.4. 74.9, 59.1, 47.4, 46.0. HRESIMS calcd for $[C_{24}H_{23}N_2O_4 + H]^+$ 371.1754, found 371.1769.

3-(2-Methoxyl-1-phenylethyl)-1-(4-methylbenzyl)quinoxalin-2(1*H*)-one (**4e**)

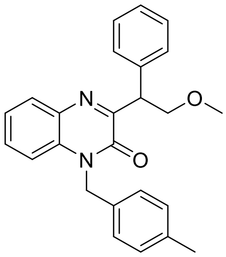

Yellow oil (46 mg, 60%). ^{1}H NMR (400 MHz, $CDCl_3$): *δ* 7.95 (d, *J* = 7.9 Hz, 1H), 7.52 (d, *J* = 7.5 Hz, 2H), 7.42 (t, *J* = 7.5 Hz, 1H), 7.33 (t, *J* = 7.1 Hz, 2H), 7.31 (t, *J* = 7.1 Hz, 1H), 7.25 (d, *J* = 7.9 Hz, 2H), 7.10 (s, 4H), 5.50 (d, *J* = 15.5 Hz, 1H), 5.29 (d, *J* = 15.5 Hz, 1H), 5.13 (t, *J* = 6.5 Hz, 1H), 4.43 (t, *J* = 9.1 Hz, 1H), 3.95 (dd, *J* = 9.1, 6.5 Hz, 1H), 3.43 (s, 3H), 2.31 (s, 3H). ^{13}C NMR (100 MHz, $CDCl_3$): *δ* 159.4, 154.6, 138.7, 137.3, 132.9, 132.5, 132.3, 130.4, 129.9, 129.5, 128.7, 128.5, 127.1, 126.9, 123.5, 114.4, 74.9, 59.0, 47.4, 45.8, 21.1. HRESIMS calcd for $[C_{25}H_{24}N_2O_2 + H]^+$ 385.1916, found 385.1929.

1-(4-Fluorobenzyl)-3-(2-methoxy-1-phenylethyl)quinoxalin-2(1*H*)-one (**4f**)

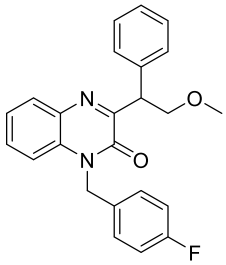

White solid (27 mg, 35%). Mp: 114-116 ℃. ^{1}H NMR (400 MHz, $CDCl_3$): *δ* 7.94 (d, *J* = 7.4 Hz, 1H), 7.48 (d, *J* = 7.2 Hz, 2H), 7.40 (t, *J* = 7.2 Hz, 1H), 7.38-7.13 (m, 7H), 6.94 (d, *J* = 8.4 Hz, 2H), 5.44 (d, *J* = 15.3 Hz, 1H), 5.27 (d, *J* = 15.3 Hz, 1H), 5.09 (t, *J* = 7.3 Hz, 1H), 4.40 (d, *J* = 9.0 Hz, 1H), 3.91 (dd, *J* = 9.0, 7.3 Hz, 1H), 3.39 (s, 3H). ^{13}C NMR (100 MHz, $CDCl_3$): *δ* 162.0 (d, *J* = 246.4 Hz), 159.4, 154.5, 138.6, 132.9, 132.3, 131.0 (d, *J* = 3.1 Hz), 130.5, 129.9, 128.8, 128.7, 128.5, 127.1, 123.6, 115.8 (d, *J* = 21.7 Hz), 114.1, 74.8, 59.0, 47.4, 45.3. ^{19}F NMR (376 MHz,

$CDCl_3$): δ -114.52; HRESIMS calcd for $[C_{24}H_{21}FN_2O_2 + H]^+$ 389.1665, found 389.1651.

1-(2,6-Dichlorobenzyl)-3-(2-methoxy-1-phenylethyl)quinoxalin-2(1*H*)-one (**4g**)

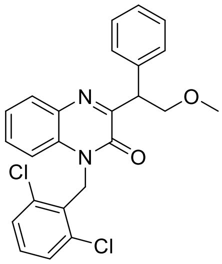

Yellow solid (29 mg, 33%). Mp: 85-86 ℃. ^1H NMR (400 MHz, $CDCl_3$): δ 8.10 (d, J = 8.0 Hz, 1H), 7.87 (d, J = 8.0 Hz, 1H), 7.65 (t, J = 7.3 Hz, 1H), 7.59 (t, J = 7.3 Hz, 1H), 7.40 (d, J = 7.6 Hz, 2H), 7.30 (t, J = 7.6 Hz, 1H), 7.26 (d, J = 7.6 Hz, 2H), 7.18 (s, 3H), 5.79 (d, J = 11.4 Hz, 1H), 5.64 (d, J = 11.4 Hz, 1H), 4.80 (t, J = 7.0 Hz, 1H), 4.48 (t, J = 8.5 Hz, 1H), 4.02 (t, J = 7.9 Hz, 1H), 3.39 (s, 3H). ^{13}C NMR (100 MHz, $CDCl_3$): δ 155.2, 149.7, 139.7, 139.0, 138.8, 137.2, 132.1, 130.5, 129.3, 128.9, 128.7, 128.4, 128.3, 126.9, 126.8, 126.4, 74.6, 63.2, 59.1, 47.4. HRESIMS calcd for $[C_{24}H_{20}Cl_2N_2O_2 + H]^+$ 439.0980, found 439.0989.

3-(2-Methoxyl-1-phenylethyl)-1-(4-(trifluoromethyl)benzyl)quinoxalin-2(1*H*)-one (**4h**)

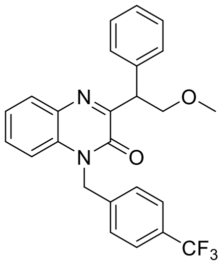

Yellow oil (39 mg, 45%). ^1H NMR (400 MHz, $CDCl_3$): δ 8.00 (d, J = 7.9 Hz, 1H), 7.55 (d, J = 7.9 Hz, 2H), 7.52 (d, J = 7.9 Hz, 2H), 7.44 (t, J = 7.7 Hz, 1H), 7.36 (t, J = 7.7 Hz, 2H), 7.33 (t, J = 7.7 Hz, 1H), 7.31 (t, J = 7.5 Hz, 2H), 7.26 (t, J = 7.5 Hz, 1H), 7.14 (d, J = 7.9 Hz, 1H), 5.56 (d, J = 15.9 Hz, 1H), 5.40 (d, J = 15.9 Hz, 1H), 5.12 (t, J = 7.3 Hz, 1H), 4.45 (t, J = 9.1 Hz, 1H), 3.94 (dt, J = 9.1, 7.3 Hz, 1H), 3.44 (s, 3H). ^{13}C NMR (100 MHz, $CDCl_3$): δ 159.4, 154.5, 139.3, 138.5, 132.9, 132.2, 130.6, 130.1, 129.8, 128.7, 128.6, 127.2, 127.1, 125.9 (d, J = 7.7 Hz), 123.9 (q, J = 272.1 Hz), 123.8, 113.9, 74.8, 59.1, 47.5, 45.6. ^{19}F NMR (376 MHz, $CDCl_3$) δ -62.64; HRESIMS calcd for $[C_{25}H_{21}F_3N_2O_2 + H]^+$ 439.1633, found 439.1621.

3-(2-Methoxy-1-phenylethyl)-1-(naphthalen-2-ylmethyl)quinoxalin-2(1*H*)-one (**4i**)

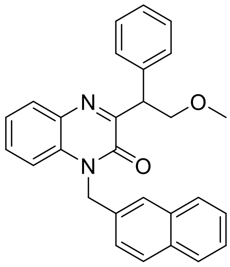

Yellow solid (29 mg, 35%). Mp: 56-58 ℃. ^1H NMR (400 MHz, $CDCl_3$): δ 7.99 (d, J = 8.0 Hz,

1H), 7.80 (d, J = 4.9 Hz, 2H), 7.72 (t, J = 4.9 Hz, 1H), 7.59 (d, J = 7.9 Hz, 2H), 7.56 (s, 1H), 7.47 (t, J = 4.9 Hz, 2H), 7.40-7.30 (m, 7H), 5.72 (d, J = 15.8 Hz, 1H), 5.48 (d, J = 15.8 Hz, 1H), 5.19 (t, J = 7.3 Hz, 1H), 4.49 (t, J = 9.0 Hz, 1H), 3.99 (t, J = 7.8 Hz, 1H), 3.47 (s, 3H). ^{13}C NMR (100 MHz, $CDCl_3$): δ 159.4, 154.7, 138.7, 133.3, 133.0, 132.8, 132.7, 132.5, 130.4, 130.0, 128.9, 128.8, 128.6, 127.8, 127.7, 127.2, 126.4, 126.1, 125.6, 124.8, 123.6, 114.4, 74.9, 59.1, 47.5, 46.3. HRESIMS calcd for $[C_{28}H_{24}N_2O_2 + H]^+$ 421.1916, found 421.1933.

1-Hexyl-3-(2-methoxy-1-phenylethyl)quinoxalin-2(1*H*)-one (**4j**)

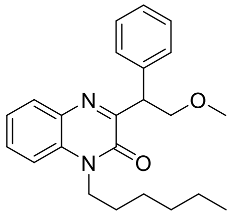

Yellow oli (35 mg, 48%). ^{1}H NMR (400 MHz, $CDCl_3$): δ 7.97 (d, J = 8.0 Hz, 1H), 7.52 (t, J = 7.0 Hz, 2H), 7.51 (s, 1H), 7.36-7.21 (m, 5H), 5.10 (t, J = 7.6 Hz, 1H), 4.42 (t, J = 9.0 Hz, 1H), 4.24 (dt, J = 7.8, 14.3 Hz, 1H), 4.11 (dt, J = 7.8, 14.3 Hz, 1H), 3.94 (t, J = 7.6 Hz, 1H), 3.42 (s, 3H), 1.71 (dt, J = 6.8, 15.5 Hz, 1H), 1.45-1.28 (m, 6H), 0.92 (t, J = 6.0 Hz, 1H). ^{13}C NMR (100 MHz, $CDCl_3$): δ 159.2, 154.2, 138.8, 133.0, 132.3, 130.5, 129.8, 128.7, 128.5, 127.1, 123.2, 113.5, 74.8, 59.0, 47.2, 42.5, 31.5, 27.2, 26.7, 22.6, 14.0. HRESIMS calcd for $[C_{23}H_{28}N_2O_2 + H]^+$ 365.2229, found 365.2211.

1-(Cyclohexylmethyl)-3-(2-methoxy-1-phenylethyl)quinoxalin-2(1*H*)-one (**4k**)

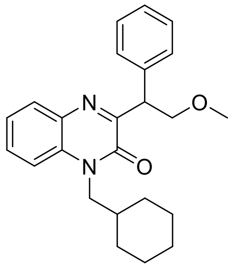

Colorless oil (48 mg, 64%). ^{1}H NMR (400 MHz, $CDCl_3$): δ 8.07 (d, J = 7.9 Hz, 1H), 7.78 (d, J = 7.9 Hz, 1H), 7.61 (t, J = 7.5 Hz, 1H), 7.55 (t, J = 7.1 Hz, 1H), 7.38 (d, J = 7.1 Hz, 2H), 7.28 (t, J = 6.8 Hz, 2H), 7.22 (d, J = 6.8 Hz, 1H), 4.85 (t, J = 6.4 Hz, 1H), 4.48 (t, J = 8.3 Hz, 1H), 4.22 (dd, J = 18.6, 5.6 Hz, 2H), 4.01 (t, J = 8.3 Hz, 1H), 3.41 (s, 3H), 1.82-1.68 (m, 7H), 1.30-1.23 (m, 2H), 1.08-1.00 (m, 2H). ^{13}C NMR (100 MHz, $CDCl_3$): δ 156.0, 149.8, 139.9, 139.4, 138.4, 129.1, 128.8, 128.7, 128.3, 126.9, 126.6, 126.0, 75.1, 71.6, 59.1, 47.5, 37.4, 29.8, 26.5, 25.8. HRESIMS calcd for $[C_{24}H_{28}N_2O_2 + H]^+$ 377.2229, found 377.2254.

Ethyl 2-(3-(2-methoxy-1-phenylethyl)-2-oxoquinoxalin-1(2*H*)-yl)acetate (**4l**)

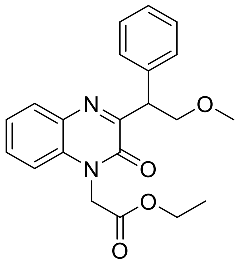

White solid (40 mg, 60%). Mp: 86-89 ℃. ^{1}H NMR (400 MHz, $CDCl_3$): δ 7.88 (dd, J = 8.0, 1.4 Hz, 1H), 7.41 (dt, J = 2.0, 8.0 Hz, 1H), 7.39 (dd, J = 3.6, 2.0 Hz, 1H), 7.37 (s, 1H), 7.28 (dt, J = 1.4, 8.0 Hz, 1H), 7.21 (t, J = 7.5 Hz, 2H), 7.13 (dt, J = 2.0, 7.5 Hz, 1H), 6.95 (d, J = 8.0 Hz, 1H), 4.95 (d, J = 17.3 Hz, 1H), 4.94 (dd, J = 8.3, 6.6 Hz, 1H), 4.78 (d, J = 17.3 Hz, 1H), 4.28 (dd, J = 9.3, 8.6 Hz, 1H), 4.11 (m, 2H), 3.83 (dd, J = 9.3, 6.6 Hz, 1H), 3.31 (s, 3H), 1.14 (t, J = 7.1 Hz, 3H). ^{13}C NMR (100 MHz, $CDCl_3$): δ 167.1, 159.0, 154.1, 138.5, 132.7, 132.2, 130.6, 130.1, 128.7, 128.6, 127.2, 123.8, 113.1, 74.7, 62.0, 59.0, 47.3, 43.7, 14.1. HRESIMS calcd for $[C_{21}H_{22}N_2O_4 + H]^+$ 367.1658, found 367.1682.

1-Allyl-3-(2-methoxy-1-phenylethyl)quinoxalin-2(1*H*)-one (**4m**)

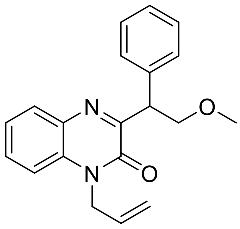

White solid (38 mg, 60%). Mp: 76-78 ℃. ^{1}H NMR (400 MHz, $CDCl_3$): δ 7.98 (d, J = 7.9 Hz, 1H), 7.53 (d, J = 7.5 Hz, 2H), 7.49 (t, J = 7.9 Hz, 1H), 7.35 (t, J = 7.9 Hz, 1H), 7.33 (t, J = 7.5 Hz, 2H), 7.26 (t, J = 8.3 Hz, 1H), 7.24 (t, J = 8.3 Hz, 1H), 5.95-5.83 (m, 1H), 5.24 (d, J = 10.3 Hz, 1H), 5.16 (d, J = 17.9 Hz, 1H), 5.11 (t, J = 7.1 Hz, 1H), 4.92 (dd, J = 15.9, 4.3 Hz, 1H), 4.77 (dd, J = 15.9, 4.3 Hz, 1H), 4.43 (t, J = 9.0 Hz, 1H), 3.94 (t, J = 7.7 Hz, 1H), 3.43 (s, 3H). ^{13}C NMR (100 MHz, $CDCl_3$): δ 159.3, 154.1, 138.7, 132.9, 132.4, 130.8, 130.4, 129.9, 128.7, 128.5, 127.1, 123.5, 118.2, 114.1, 74.9, 59.0, 47.3, 44.7. HRESIMS calcd for $[C_{20}H_{20}N_2O_2 + H]^+$ 321.1603, found 321.1625.

3-(2-Methoxy-1-phenylethyl)-1-(prop-2-yn-1-yl)quinoxalin-2(1*H*)-one (**4n**)

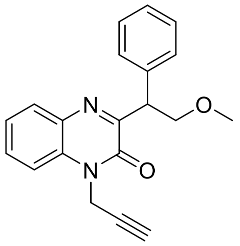

Yellow solid (38 mg, 60%). Mp: 87-90 ℃. ^{1}H NMR (400 MHz, $CDCl_3$): δ 7.97 (d, J = 7.6 Hz, 1H), 7.57 (t, J = 7.6 Hz, 1H), 7.50 (d, J = 7.1 Hz, 2H), 7.44 (d, J = 8.3 Hz, 1H), 7.39 (t, J = 8.3 Hz, 1H), 7.31 (d, J = 7.1 Hz, 2H), 7.24 (t, J = 7.1 Hz, 1H), 5.08 (d, J = 17.4 Hz, 1H), 5.07 (t, J = 6.8 Hz, 1H), 4.90 (d, J = 17.4 Hz, 1H), 4.40 (t, J = 9.0 Hz, 1H), 3.90 (d, J = 7.9 Hz, 1H), 3.41 (s, 3H), 2.27 (s, 1H). ^{13}C NMR (100 MHz, $CDCl_3$): δ 159.1, 153.5, 138.4, 132.9, 131.6, 130.4, 130.0,

128.7, 128.5, 127.2, 123.8, 114.1, 76.8, 74.8, 73.2, 59.0, 47.3, 31.6. HRESIMS calcd for $[C_{20}H_{18}N_2O_2 + H]^+$ 319.1447, found 319.1469.

3-(2-Methoxy-1-phenylethyl)quinoxalin-2(1*H*)-one (**4o**)

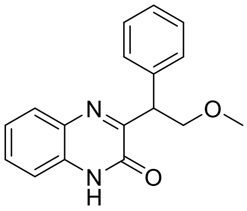

Yellow solid (32 mg, 58%). Mp: 160-163 ℃. ^{1}H NMR (400 MHz, $CDCl_3$): δ 12.24 (s, 1H), 7.92 (d, J = 8.0 Hz, 1H), 7.50 (d, J = 7.6 Hz, 1H), 7.49 (t, J = 8.5 Hz, 2H), 7.35 (t, J = 7.6 Hz, 1H), 7.30 (t, J = 8.5 Hz, 2H), 7.22 (t, J = 8.0 Hz, 2H), 5.07 (t, J = 7.0 Hz, 1H), 4.41 (t, J = 8.8 Hz, 1H), 3.95 (t, J = 7.6 Hz, 1H), 3.41 (s, 3H). ^{13}C NMR (100 MHz, $CDCl_3$): δ 159.7, 156.2, 138.6, 132.8, 130.9, 129.9, 129.2, 128.7, 128.5, 127.1, 124.0, 115.6, 74.8, 59.1, 46.8. HRESIMS calcd for $[C_{17}H_{16}N_2O_2 + H]^+$ 281.1290, found 281.1296.

3-(2-Methoxyl-1-(*p*-tolyl)ethyl)-1-methylquinoxalin-2(1*H*)-one (**4p**)

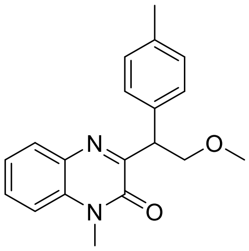

White solid (34 mg, 55%). Mp: 107-110 ℃. ^{1}H NMR (400 MHz, $CDCl_3$): δ 7.95 (d, J = 7.5 Hz, 1H), 7.53 (t, J = 8.1 Hz, 1H), 7.40 (d, J = 7.7 Hz, 2H), 7.36 (t, J = 7.5 Hz, 1H), 7.26 (d, J = 8.1 Hz, 1H), 7.13 (d, J = 7.7 Hz, 2H), 5.04 (t, J = 6.7 Hz, 1H), 4.40 (t, J = 9.0 Hz, 1H), 3.92 (dd, J = 9.0, 6.7 Hz, 1H), 3.63 (s, 3H), 3.42 (s, 3H), 2.31 (s, 3H). ^{13}C NMR (100 MHz, $CDCl_3$): δ 159.4, 154.5, 136.7, 135.6, 133.1, 132.7, 130.2, 129.9, 129.3, 128.6, 123.4, 113.5, 74.8, 59.0, 46.9, 29.1, 21.1. HRESIMS calcd for $[C_{19}H_{20}N_2O_2 + H]^+$ 309.1603, found 309.1021.

3-(1-(4-(*tert*-Butyl)phenyl)-2-methoxyl)-1-methylquinoxalin-2(1*H*)-one (**4q**)

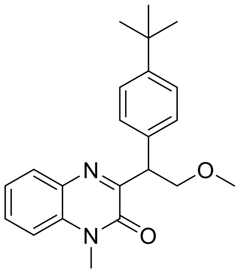

Yellow oil (32 mg, 55%). ^{1}H NMR (400 MHz, $CDCl_3$): δ 7.95 (dd, J = 8.3, 1.1 Hz, 1H), 7.52 (dt, J = 1.4, 8.6 Hz, 1H), 7.43 (dt, J = 1.4, 8.6 Hz, 2H), 7.36 (dt, J = 1.1, 8.3 Hz, 1H), 7.33 (dt, J = 1.4, 8.6 Hz, 2H), 7.26 (d, J = 8.3 Hz, 1H), 5.07 (dd, J = 9.2, 6.0 Hz, 1H), 4.42 (t, J = 9.2 Hz, 1H), 3.90 (dd, J = 9.2, 6.0 Hz, 1H), 3.64 (s, 3H), 3.41 (s, 3H), 1.29 (s, 9H). ^{13}C NMR (100 MHz, $CDCl_3$): δ 159.4, 154.6, 149.7, 135.4, 133.1, 132.7, 130.2, 129.8, 128.3, 125.5, 123.4, 113.5, 74.8, 59.0, 46.7,

34.4, 31.3, 29.1. HRESIMS calcd for [$C_{22}H_{26}N_2O_2$ + H]$^+$ 351.2073, found 351.2075.

3-(1-(4-Chlorophenyl)-2-methoxyethyl)-1-methylquinoxalin-2(1*H*)-one (**4r**)

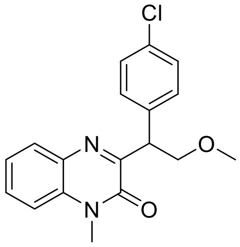

Yellow solid (28 mg, 43%). Mp: 124-126 ℃. ^{1}H NMR (400 MHz, $CDCl_3$): δ 7.94 (d, *J* = 8.0 Hz, 1H), 7.55 (d, *J* = 7.8 Hz, 1H), 7.44 (d, *J* = 8.0 Hz, 2H), 7.37 (t, *J* = 7.8 Hz, 1H), 7.28 (s, 1H), 7.27 (d, *J* = 8.0 Hz, 2H), 5.03 (t, *J* = 7.4 Hz, 1H), 4.32 (t, *J* = 8.8 Hz, 1H), 3.92 (t, *J* = 8.1 Hz, 1H), 3.64 (s, 3H), 3.40 (s, 3H). ^{13}C NMR (100 MHz, $CDCl_3$): δ 158.8, 154.4, 137.2, 133.1, 132.9, 132.6, 130.3, 130.2, 130.1, 128.6, 123.6, 113.6, 74.6, 59.0, 46.7, 29.2. HRESIMS calcd for [$C_{18}H_{17}ClN_2O_2$ + H]$^+$ 329.1057, found 329.1077.

3-(2-Methoxyl-1-(4-(trifluoromethyl)phenyl)ethyl)-1-methylquinoxalin-2(1*H*)-one (**4s**)

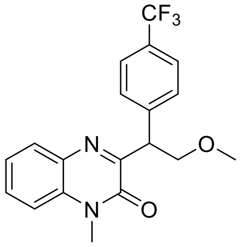

White solid (36 mg, 50%). Mp: 118-122 ℃. ^{1}H NMR (400 MHz, $CDCl_3$): δ 7.95 (d, *J* = 8.0 Hz, 1H), 7.63 (d, *J* = 8.1 Hz, 2H), 7.57 (t, *J* = 8.0 Hz, 1H), 7.56 (d, *J* = 8.1 Hz, 2H), 7.39 (t, *J* = 7.6 Hz, 1H), 7.30 (d, *J* = 7.6 Hz, 1H), 5.11 (t, *J* = 7.3 Hz, 1H), 4.34 (t, *J* = 8.6 Hz, 1H), 3.98 (t, *J* = 8.6 Hz, 1H), 3.66 (s, 3H), 3.41 (s, 3H). ^{13}C NMR (100 MHz, $CDCl_3$): δ 158.4, 154.4, 142.9, 133.1, 132.6, 130.3, 129.4, 129.1, 128.8, 125.4 (d, *J* = 3.8 Hz), 124.2 (d, *J* = 272.0 Hz), 123.7, 113.6, 74.5, 59.1, 47.2, 29.2. ^{19}F NMR (376 MHz, $CDCl_3$) δ -62.49; HRESIMS calcd for [$C_{19}H_{17}F_3N_2O_2$ + H]$^+$ 363.1320, found 363.1334.

4-(2-Methoxy-1-(4-methyl-3-oxo-3,4-dihydroquinoxalin-2-yl)ethyl)phenyl acetate (**4t**)

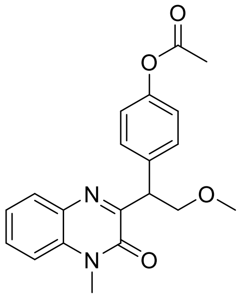

Yellow solid (37 mg, 52%). Mp:128-130 ℃. ^{1}H NMR (400 MHz, $CDCl_3$): δ 7.91 (dd, *J* = 8.0, 1.4 Hz, 1H), 7.52 (dt, *J* = 1.4, 8.5 Hz, 1H), 7.49 (dt, *J* = 2.6, 7.6 Hz, 2H), 7.34 (dt, *J* = 1.4, 7.6 Hz, 1H), 7.25 (d, *J* = 8.5 Hz, 1H), 6.99 (dt, *J* = 2.6, 8.6 Hz, 2H), 5.06 (dd, *J* = 8.6, 6.3 Hz, 1H), 4.36 (t, *J* = 9.0 Hz, 1H), 3.88 (dd, *J* = 9.0, 6.3 Hz, 1H), 3.63 (s, 3H), 3.38 (s, 3H), 2.26 (s, 3H). ^{13}C NMR (100

MHz, $CDCl_3$): δ 169.6, 158.9, 154.5, 149.7, 136.1, 133.1, 132.6, 130.2, 130.1, 129.8, 123.5, 121.5, 113.6, 74.6, 59.0, 46.6, 29.2, 21.2. HRESIMS calcd for $[C_{20}H_{20}N_2O_4 + H]^+$ 353.1501, found 353.1487.

3-(1-(3-Bromophenyl)-2-methoxyethyl)-1-methylquinoxalin-2(1*H*)-one (**4u**)

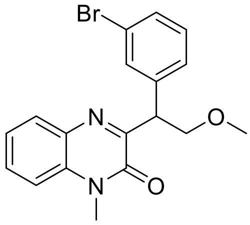

White solid (34 mg, 45%). Mp: 96-101 ℃. ^1H NMR (400 MHz, $CDCl_3$): δ 7.94 (d, *J* = 8.2 Hz, 1H), 7.62 (d, *J* = 1.1 Hz, 1H), 7.53 (t, *J* = 7.8 Hz, 1H), 7.45 (d, *J* = 7.8 Hz, 1H), 7.36 (t, *J* = 7.1 Hz, 1H), 7.35 (d, *J* = 7.1 Hz, 1H), 7.26 (d, *J* = 8.2 Hz, 1H), 7.18 (t, *J* = 7.8 Hz, 1H), 5.02 (t, *J* = 7.7 Hz, 1H), 4.32 (t, *J* = 8.8 Hz, 1H), 3.92 (t, *J* = 8.8, 7.7 Hz, 1H), 3.63 (s, 3H), 3.40 (s, 3H). ^{13}C NMR (100 MHz, $CDCl_3$): δ 158.5, 154.4, 141.1, 133.1, 132.6, 131.5, 130.3, 130.2, 130.1, 130.0, 127.7, 123.6, 122.5, 113.6, 74.6, 59.1, 46.9, 29.2. HRESIMS calcd for $[C_{18}H_{17}BrN_2O_2 + H]^+$ 373.0552, found 373.0558.

3-(2-Methoxyl-1-(*o*-tolyl)ethyl)-1-methylquinoxalin-2(1*H*)-one (**4v**)

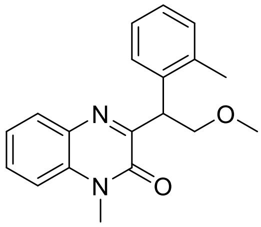

Yellow solid (32 mg, 52%). Mp:129-132 ℃. ^1H NMR (400 MHz, $CDCl_3$): δ 8.00 (d, *J* = 7.8 Hz, 1H), 7.54 (t, *J* = 7.8 Hz, 1H), 7.38 (t, *J* = 7.6 Hz, 1H), 7.29-7.22 (m, 3H), 7.13 (t, *J* = 7.3 Hz, 1H), 7.08 (t, *J* = 7.3 Hz, 1H), 5.30 (dd, *J* = 8.6, 5.7 Hz, 1H), 4.42 (t, *J* = 9.2 Hz, 1H), 3.75 (dd, *J* = 9.2, 5.7 Hz, 1H), 3.63 (s, 3H), 3.42 (s, 3H), 2.72 (s, 3H). ^{13}C NMR (100 MHz, $CDCl_3$): δ 159.6, 154.6, 137.5, 136.9, 133.1, 132.7, 130.7, 130.3, 129.9, 127.1, 126.9, 125.8, 123.4, 113.5, 74.8, 59.1, 42.9, 29.1, 20.0. HRESIMS calcd for $[C_{19}H_{20}N_2O_2 + H]^+$ 309.1603, found 309.1612.

3-(1-(2,5-Dimethylphenyl)-2-methoxyethyl)-1-methylquinoxalin-2(1*H*)-one (**4w**)

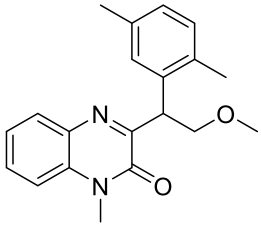

Yellow solid (22 mg, 35%). Mp: 58-61 ℃. ^1H NMR (400 MHz, $CDCl_3$): δ 8.02 (d, *J* = 8.0 Hz, 1H), 7.55 (t, *J* = 7.6 Hz, 1H), 7.40 (t, *J* = 7.6 Hz, 1H), 7.28 (d, *J* = 8.0 Hz, 1H), 7.13 (d, *J* = 7.6 Hz, 1H), 7.04 (s, 1H), 6.95 (d, *J* = 7.6 Hz, 1H), 5.26 (dd, *J* = 8.8, 5.5 Hz, 1H), 4.42 (t, *J* = 9.2 Hz, 1H), 3.75 (dd, *J* = 9.2, 5.5 Hz, 1H), 3.63 (s, 3H), 3.43 (s, 3H), 2.68 (s, 3H), 2.23 (s, 3H). ^{13}C NMR (100

MHz, $CDCl_3$): *δ* 159.7, 154.6, 136.6, 135.1, 134.3, 133.1, 132.7, 130.6, 130.3, 129.8, 127.8, 127.7, 123.4, 113.5, 74.7, 59.0, 43.0, 29.1, 21.1, 19.6. HRESIMS calcd for $[C_{20}H_{22}N_2O_2 + H]^+$ 323.1760, found 323.1781.

3-(1-(2-Chlorophenyl)-2-methoxyethyl)-1-methylquinoxalin-2(1*H*)-one (**4x**)

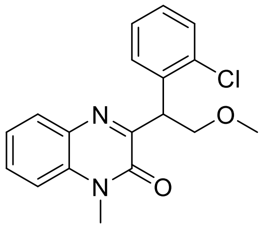

Yellow solid (16 mg, 25%). Mp: 94-96 ℃. ^{1}H NMR (400 MHz, $CDCl_3$): *δ* 7.96 (d, *J* = 7.9 Hz, 1H), 7.57 (t, *J* = 7.9 Hz, 1H), 7.44 (d, *J* = 7.5 Hz, 1H), 7.38 (t, *J* = 7.5 Hz, 1H), 7.32 (t, *J* = 8.0 Hz, 1H), 7.31 (d, *J* = 8.0 Hz, 1H), 7.16 (dt, *J* = 4.9, 8.0 Hz, 2H), 5.61 (t, *J* = 7.0 Hz, 1H), 4.33 (t, *J* = 9.0 Hz, 1H), 3.82 (dd, *J* = 9.0, 5.7 Hz, 1H), 3.67 (s, 3H), 3.44 (s, 3H). ^{13}C NMR (100 MHz, $CDCl_3$): *δ* 158.7, 154.5, 136.4, 135.0, 133.2, 132.6, 130.4, 130.2, 129.9, 128.8, 128.2, 126.6, 123.5, 113.6, 73.7, 59.0, 43.4, 29.2. HRESIMS calcd for $[C_{18}H_{17}ClN_2O_2 + H]^+$ 329.1057, found 329.1077.

3-(2-Methoxyl-1-(naphthalen-2-yl)ethyl)-1-methylquinoxalin-2(1*H*)-one (**4y**)

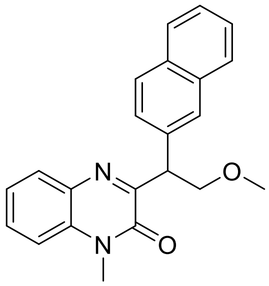

Yellow solid (18 mg, 26%). Mp: 107-109 ℃. ^{1}H NMR (400 MHz, $CDCl_3$): *δ* 8.00 (d, *J* = 8.0 Hz, 1H), 7.91 (s, 1H), 7.83-7.76 (m, 3H), 7.67 (d, *J* = 8.4 Hz, 1H), 7.54 (t, *J* = 8.0 Hz, 1H), 7.46-7.40 (s, 2H), 7.39 (d, *J* = 7.6 Hz, 1H), 7.27 (d, *J* = 8.4 Hz, 1H), 5.23 (t, *J* = 7.3 Hz, 1H), 4.49 (t, *J* = 9.0 Hz, 1H), 4.03 (t, *J* = 8.0 Hz, 1H), 3.63 (s, 3H), 3.43 (s, 3H). ^{13}C NMR (100 MHz, $CDCl_3$): *δ* 159.1, 154.5, 136.2, 133.5, 133.1, 132.7, 132.6, 130.3, 130.0, 128.2, 127.9, 127.6, 127.5, 126.9, 125.9, 125.6, 123.5, 113.6, 74.7, 59.1, 47.5, 29.2. HRESIMS calcd for $[C_{22}H_{20}N_2O_2 + H]^+$ 345.1603, found 345.1615.

3-(2-ethoxy-1-phenylethyl)-1-methylquinoxalin-2(1*H*)-one (**4z**)

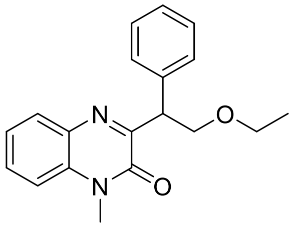

Yellow solid (38 mg, 62%). Mp: 72-74℃. ^{1}H NMR (400 MHz, $CDCl_3$): *δ* 7.93 (dd, *J* = 8.0, 1.3 Hz, 1H), 7.51 (dt, *J* = 1.3, 8.5 Hz, 1H), 7.46 (d, *J* = 7.3 Hz, 2H), 7.34 (t, *J* = 1.0, 8.0 Hz, 1H), 7.28 (t, *J*

= 7.3 Hz, 1H), 7.26 (t, *J* = 8.5 Hz, 2H), 7.20 (dt, *J* = 1.0, 7.3 Hz, 1H), 5.03 (dd, *J* = 8.8, 6.0 Hz, 1H), 4.44 (t, *J* = 9.2 Hz, 1H), 3.90 (dd, *J* = 9.5, 6.0 Hz, 1H), 3.62 (s, 3H), 3.56 (q, *J* = 7.0 Hz, 2H), 1.13 (t, *J* = 7.0 Hz, 3H). ^{13}C NMR (100 MHz, $CDCl_3$): δ 159.3, 154.6, 138.8, 133.1, 132.7, 130.2, 129.9, 128.7, 128.5, 127.0, 123.4, 113.5, 72.5, 66.5, 47.5, 29.1, 15.1. HRESIMS calcd for $[C_{19}H_{20}N_2O_2 + H]^+$ 309.1603, found 309.1620.

8. References

1. X.-K. He, J. Lu, A.-J. Zhang, Q.-Q. Zhang, G.-Y. Xu, J. Xuan. BI-OAc-accelerated C3-H alkylation of quinoxalin-2(1*H*)-ones under visible-light irradiation. *Org. Lett.*, **2020**, *22*, 5984-5989.
2. Jr. W. S. Hummers, R. E. Offeman. Preparation of graphitic oxide. *J. Am. Chem. Soc.*, **1958**, *80*, 1339−1339.
3. (a) T. Soejima, Y. Maru, S. Ito. Facile low-temperature synthesis and photocatalytic activity of graphene oxide/TiO_2 composite. *Bull. Chem. Soc. Jpn.*, **2013**, *86*, 1065−1070. (b) C.-M. Chen, J.-Q. Huang, Q. Zhang, W.-Z. Gong, Q.-H. Yang, M.-Z. Wang, Y.-G. Yang. Annealing a graphene oxide film to produce a free standing high conductive graphene film. *Carbon*, **2012**, *50*, 659−667. (c) C. W. Lo, D. Zhu, H. Jiang. An infrared-light responsive graphene-oxide incorporated poly (N-isopropylacrylamide) hydrogel nanocomposite. *Soft Matter*, **2011**, *7*, 5604−5609. (d) S, Lv. Y. Ma, C. Qiu, T. Sun, J. Liu, Q. Zhou. Effect of graphene oxide nanosheets of microstructure and mechanical properties of cement composites. *Constr. Build. Mater.*, **2013**, *49*, 121−127.
4. D. A. Dikin, S. Stankovich, E. J. Zimney, R. D. Piner, G. H. B. Dommett, G. Evmenenko, S. T. Nguyen, R. S. Ruoff. Preparation and characterization of graphene oxide paper. *Nature*, **2007**, *448*, 457−460.

9. Copies of ^{1}H NMR, ^{19}F NMR and ^{13}C NMR Spectra for All Compounds

^{1}H and ^{13}C NMR Spectra for **4a**

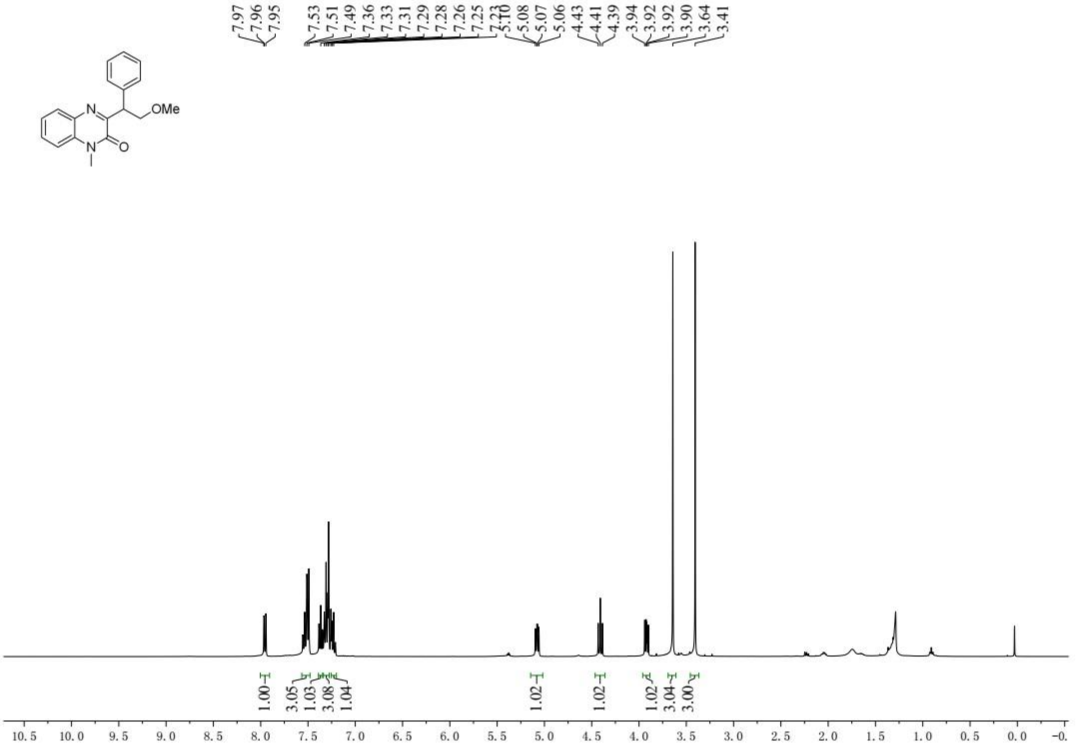

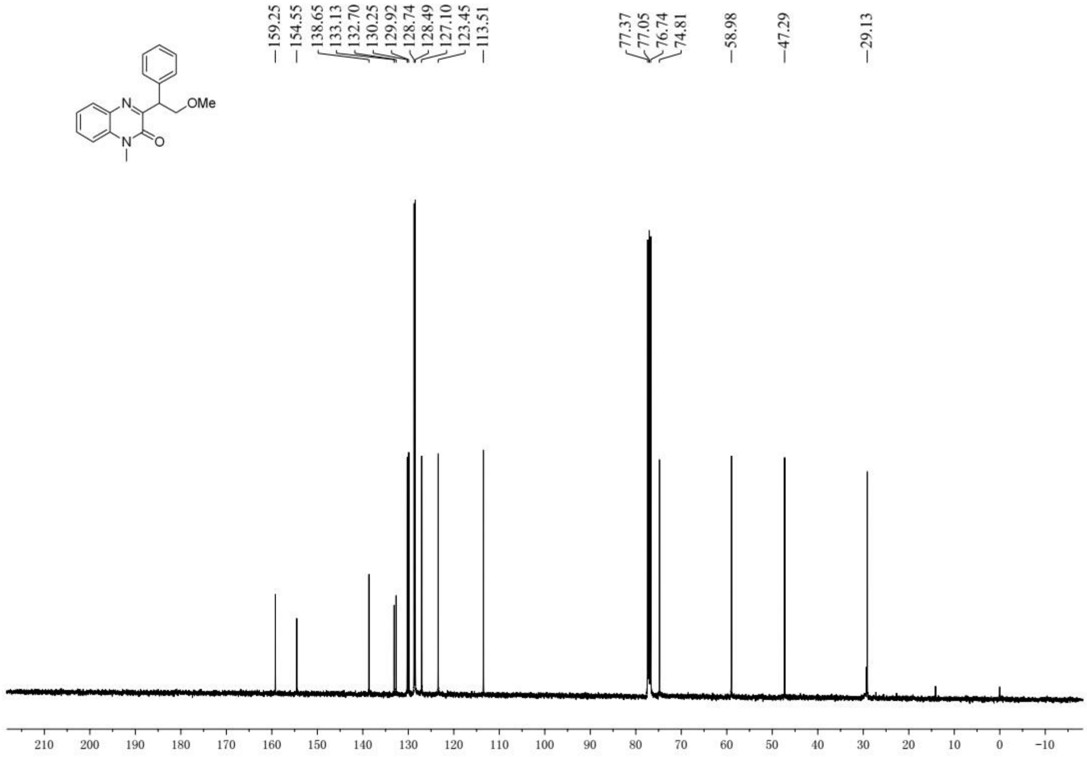

^{1}H and ^{13}C NMR Spectra for **4b**

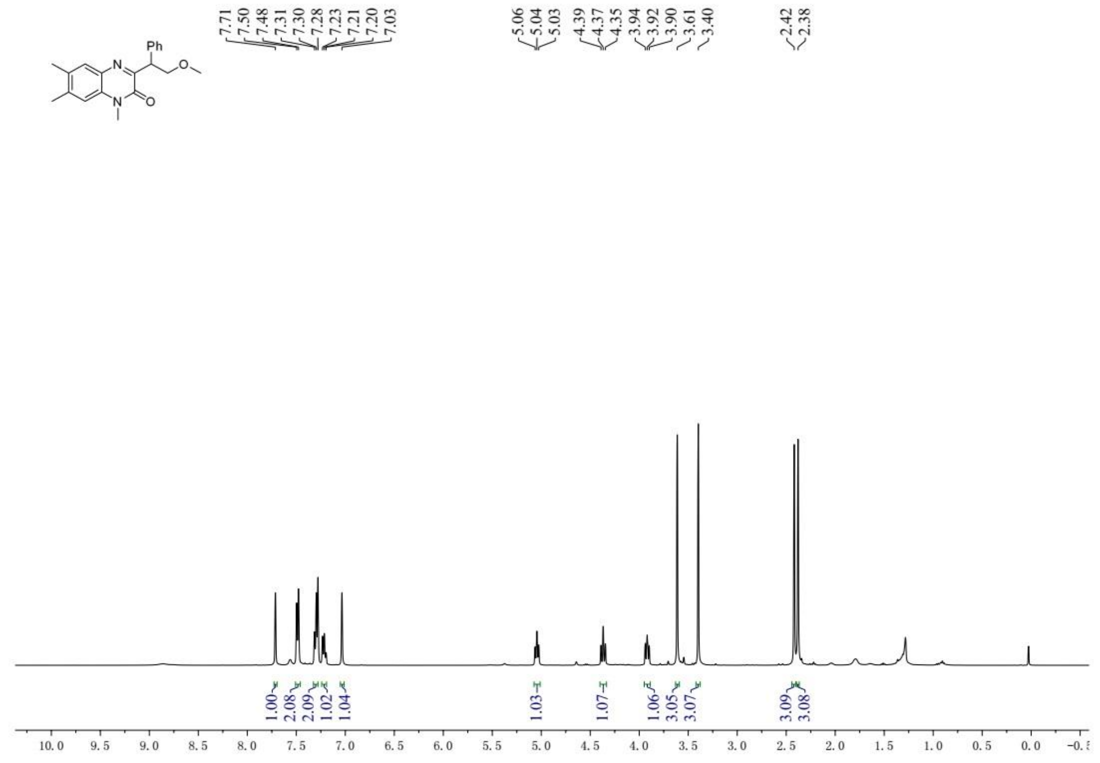

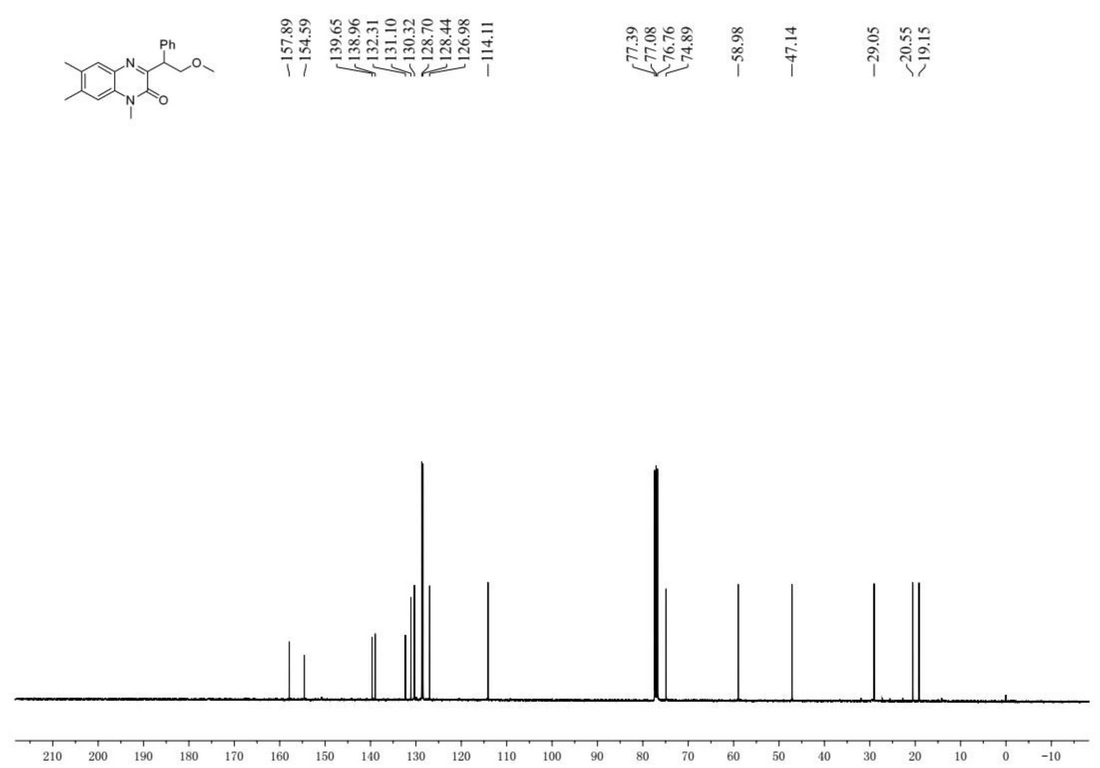

^{1}H and ^{13}C NMR Spectra for **4c**

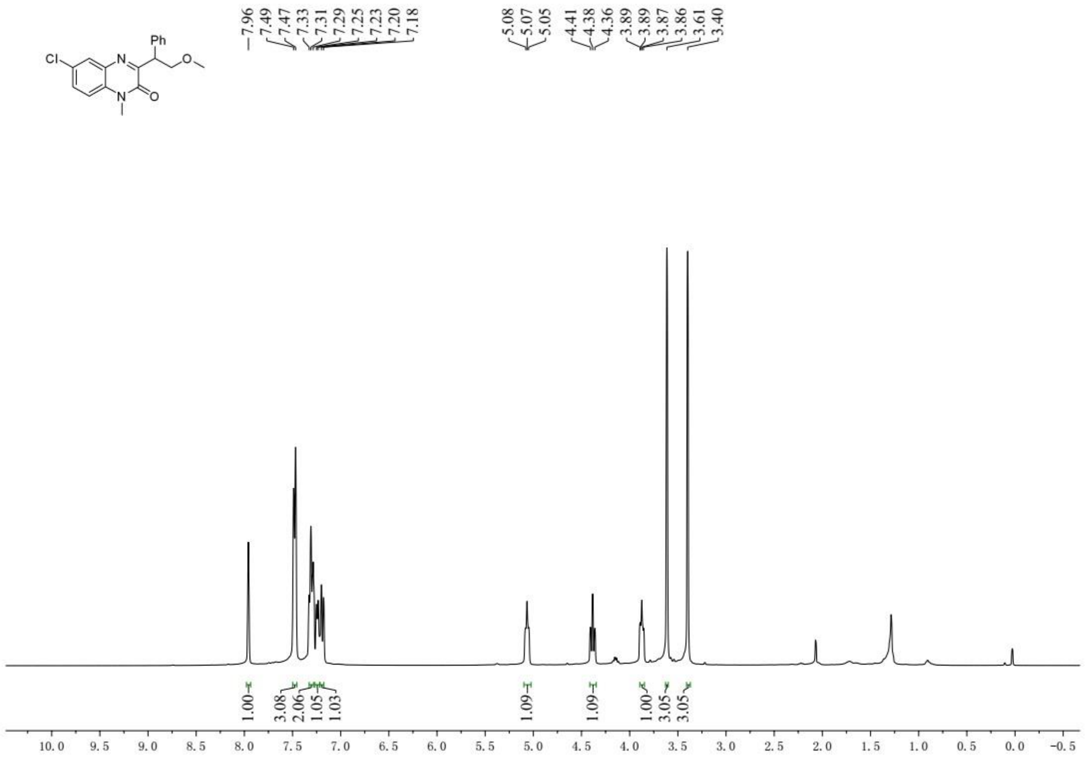

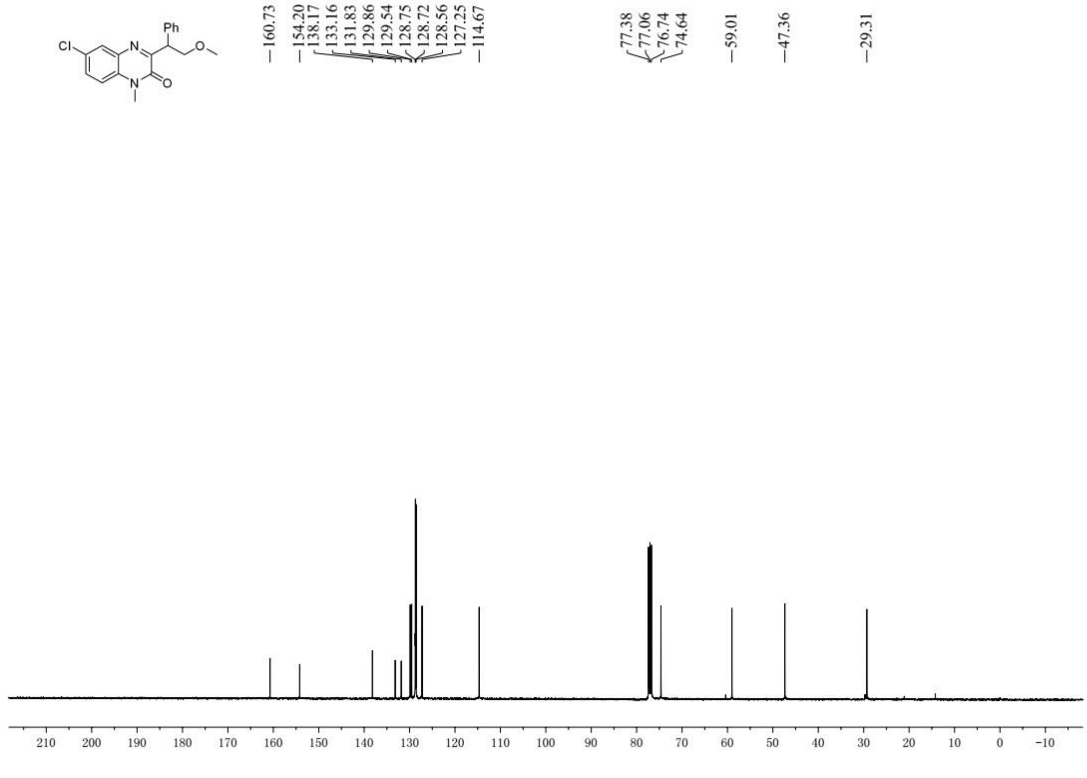

^{1}H and ^{13}C NMR Spectra for **4d**

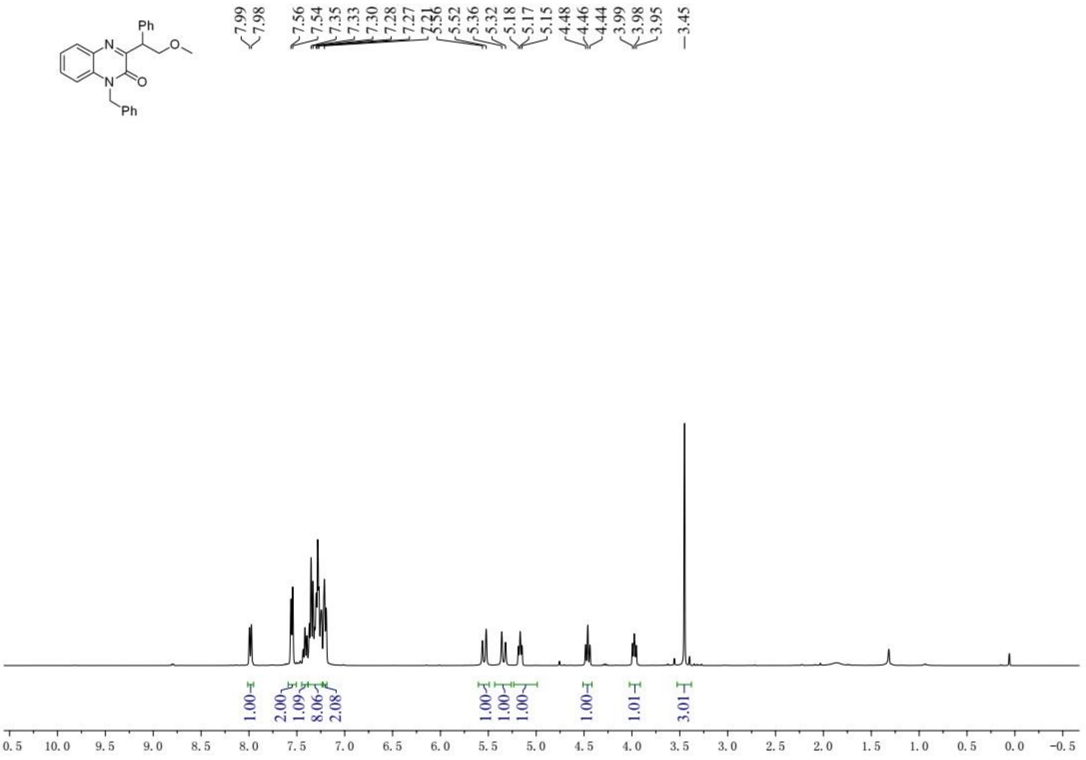

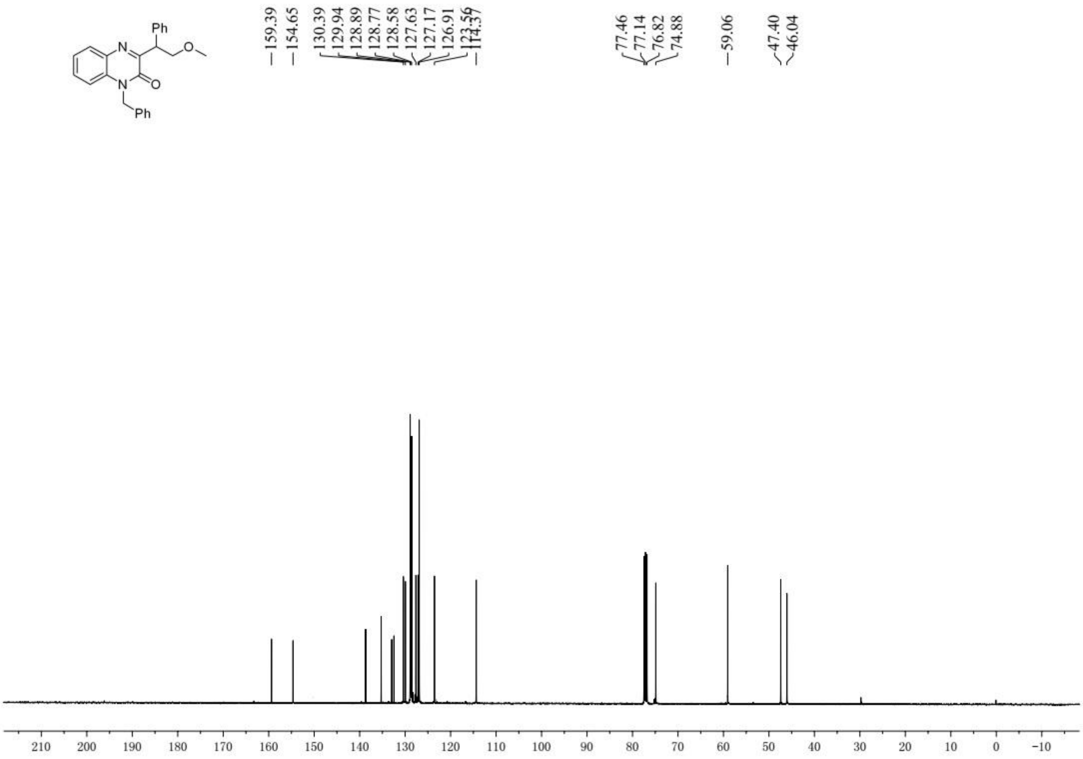

^{1}H and ^{13}C NMR Spectra for **4e**

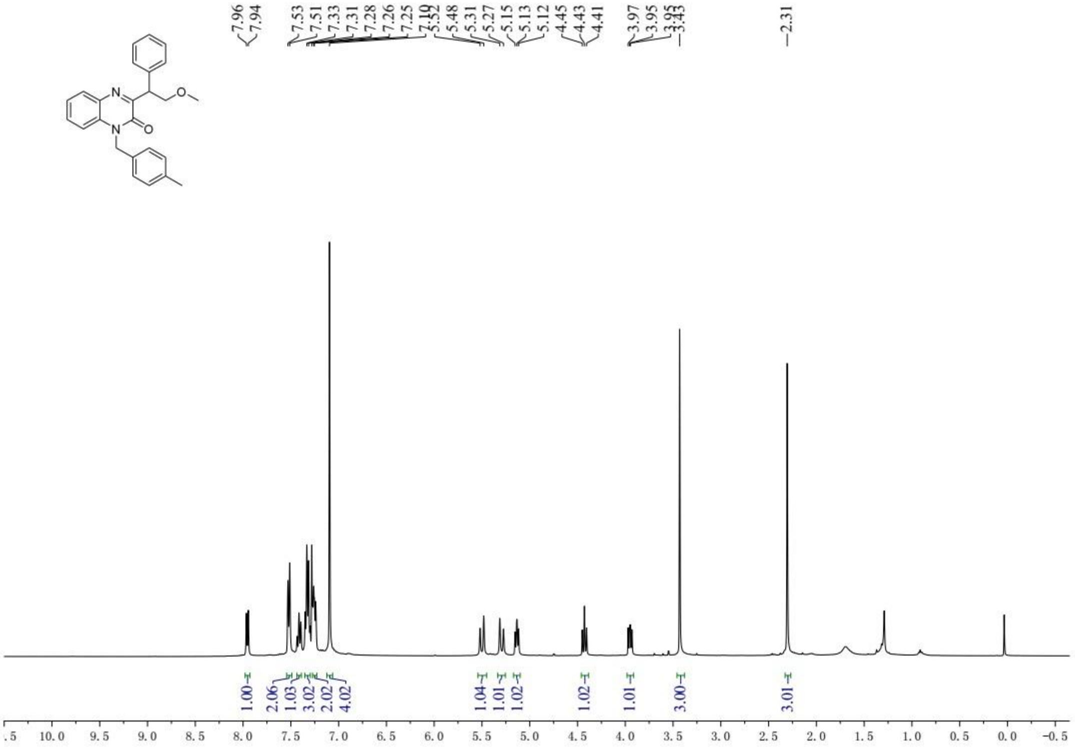

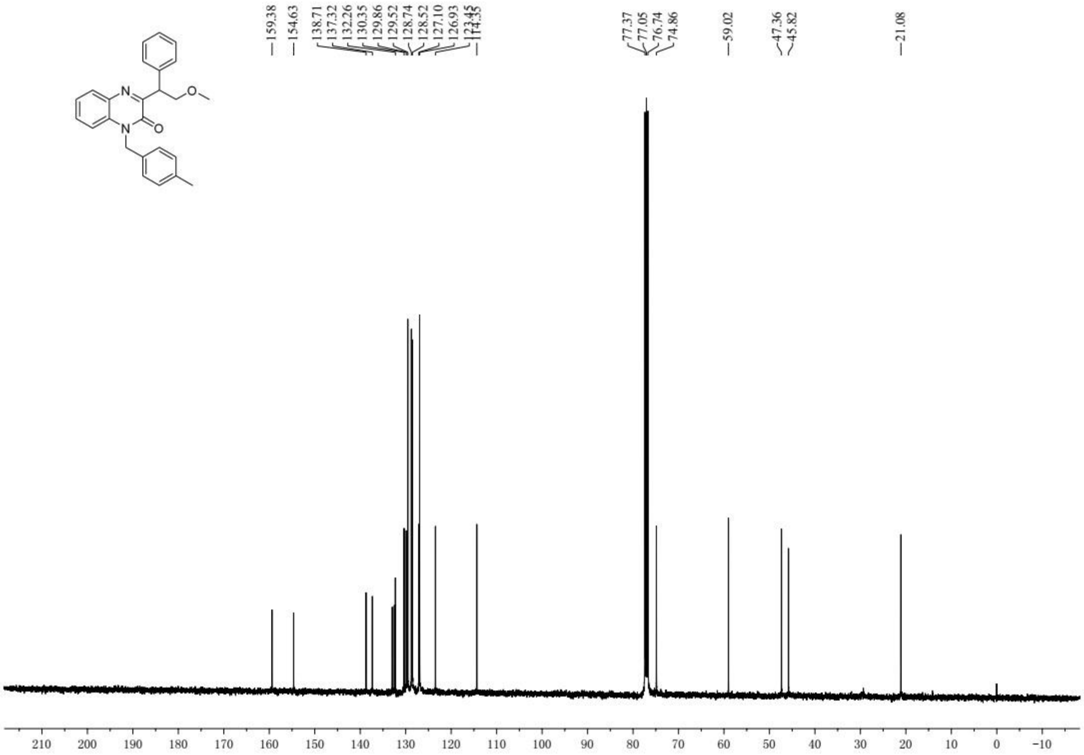

^{1}H and ^{13}C NMR Spectra for **4f**

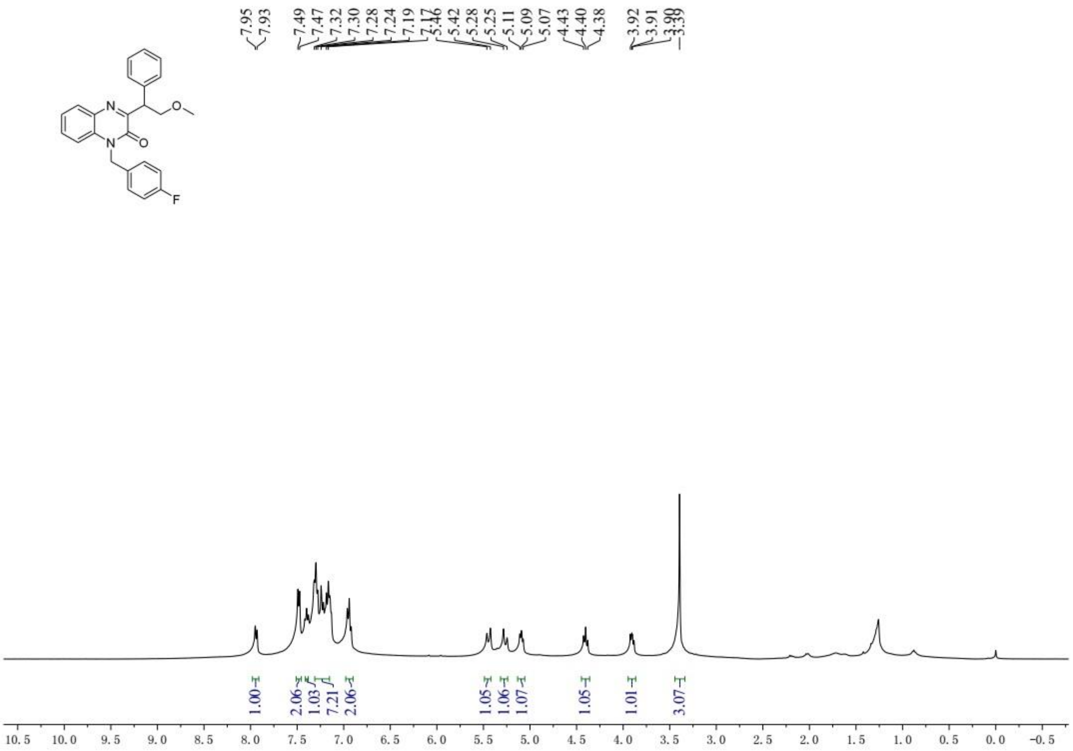

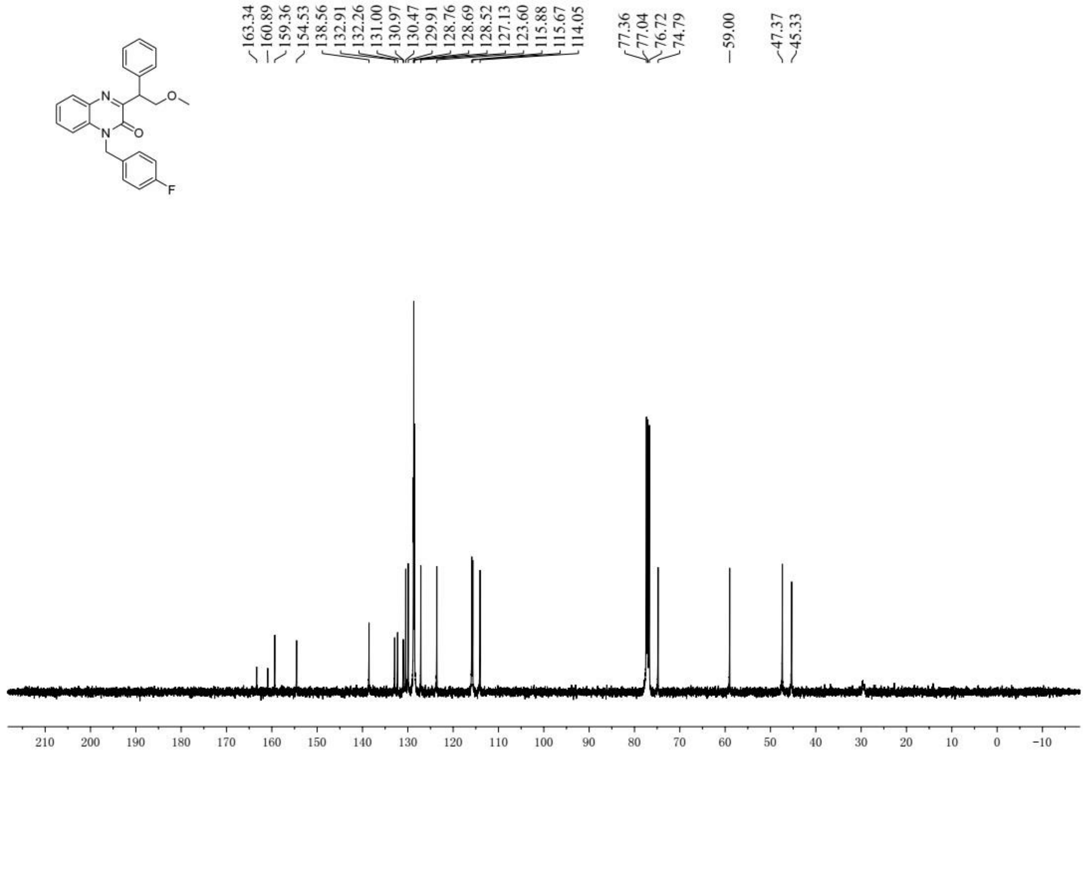

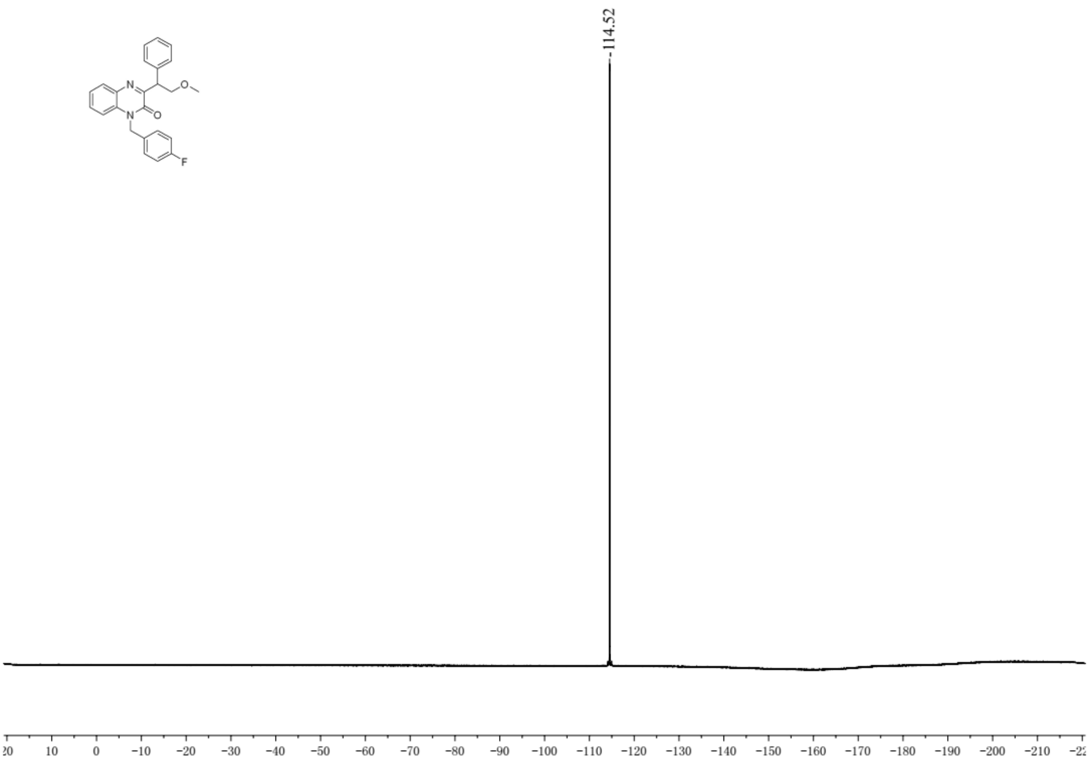
-114.52
N
N
O
O
F
20 10 0 −10 −20 −30 −40 −50 −60 −70 −80 −90 −100 −110 −120 −130 −140 −150 −160 −170 −180 −190 −200 −210

^{1}H and ^{13}C NMR Spectra for **4g**

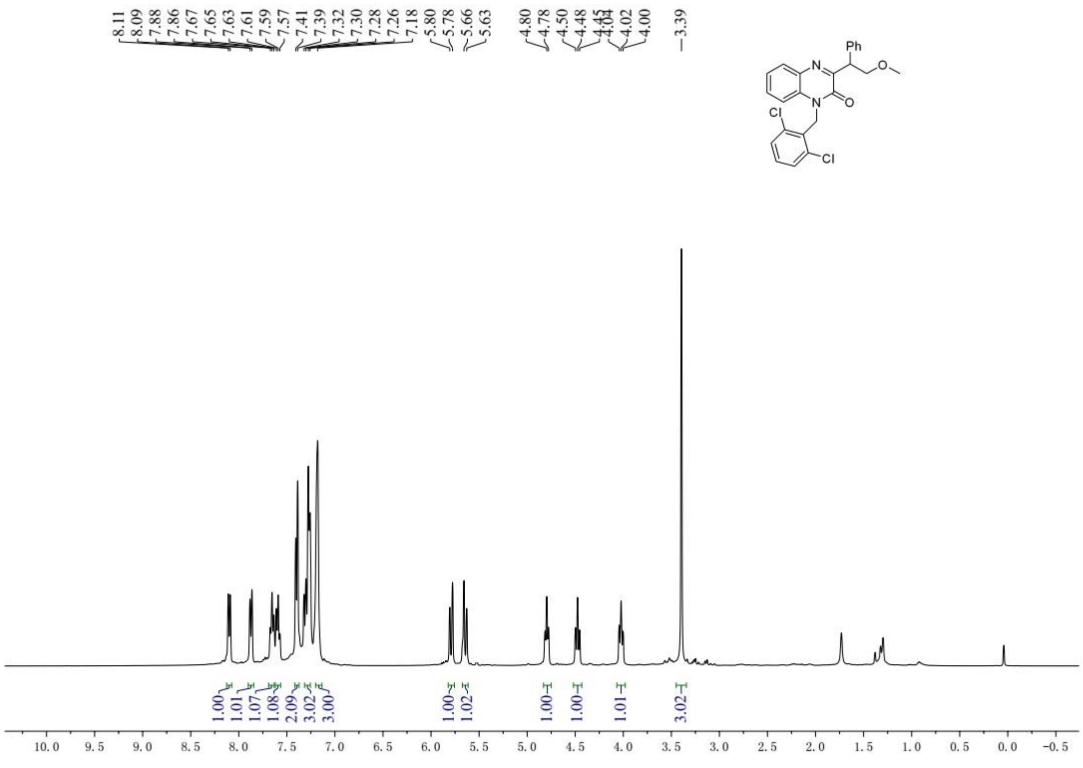

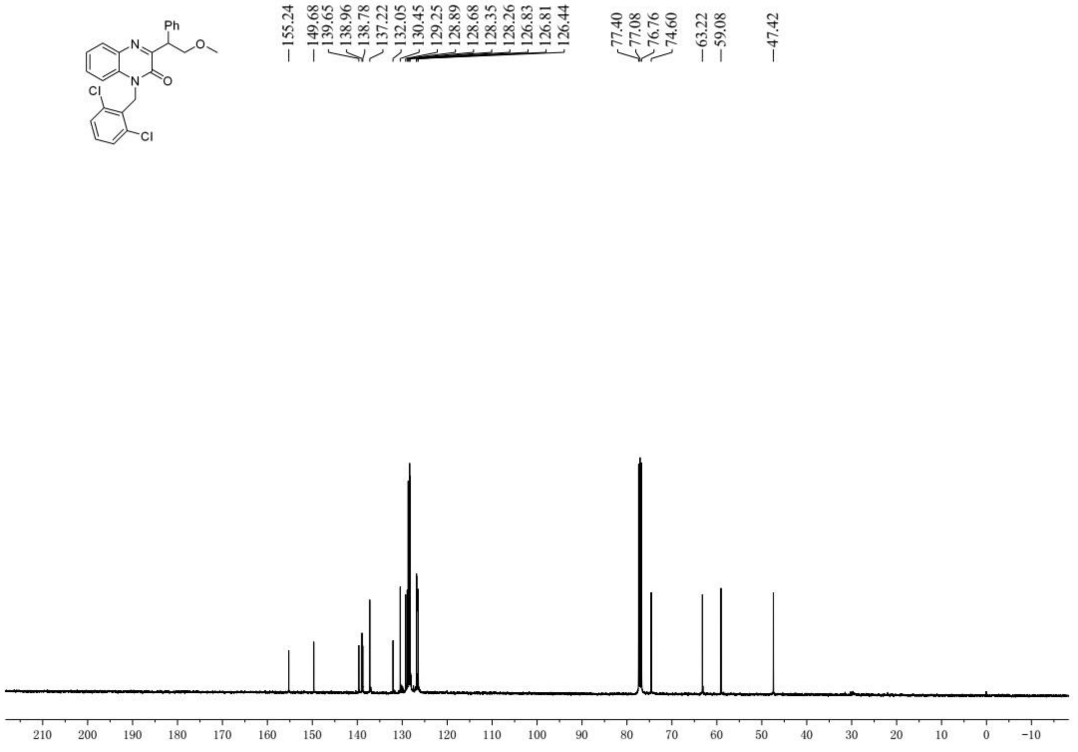

^{1}H and ^{13}C NMR Spectra for **4h**

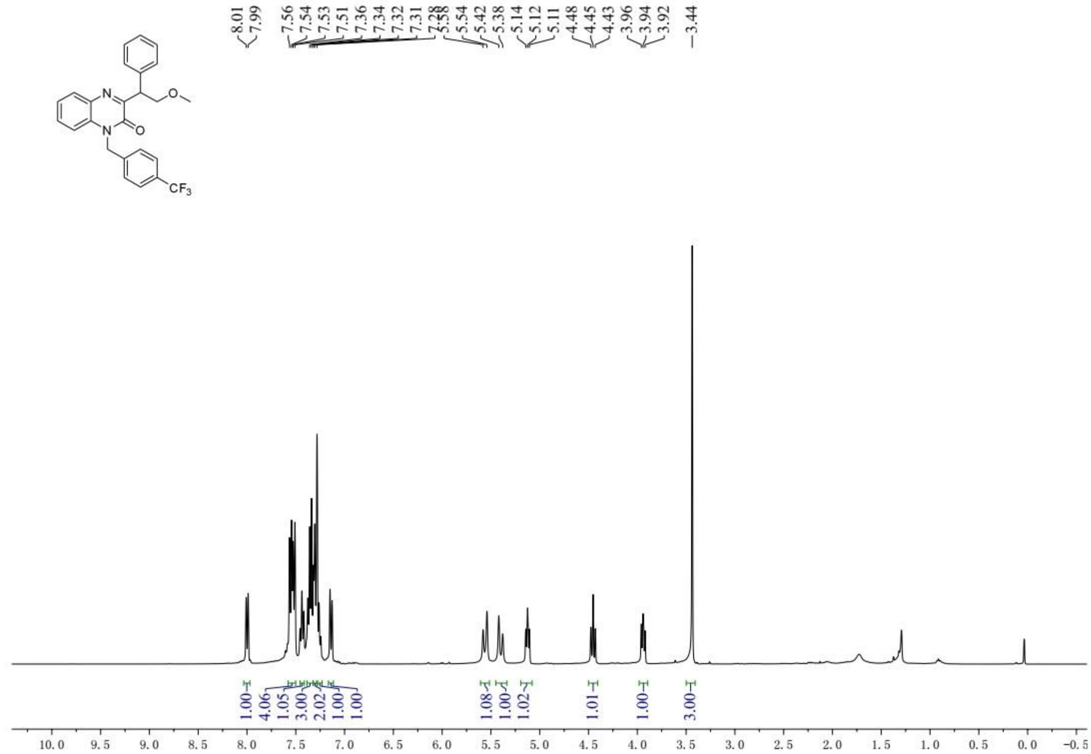

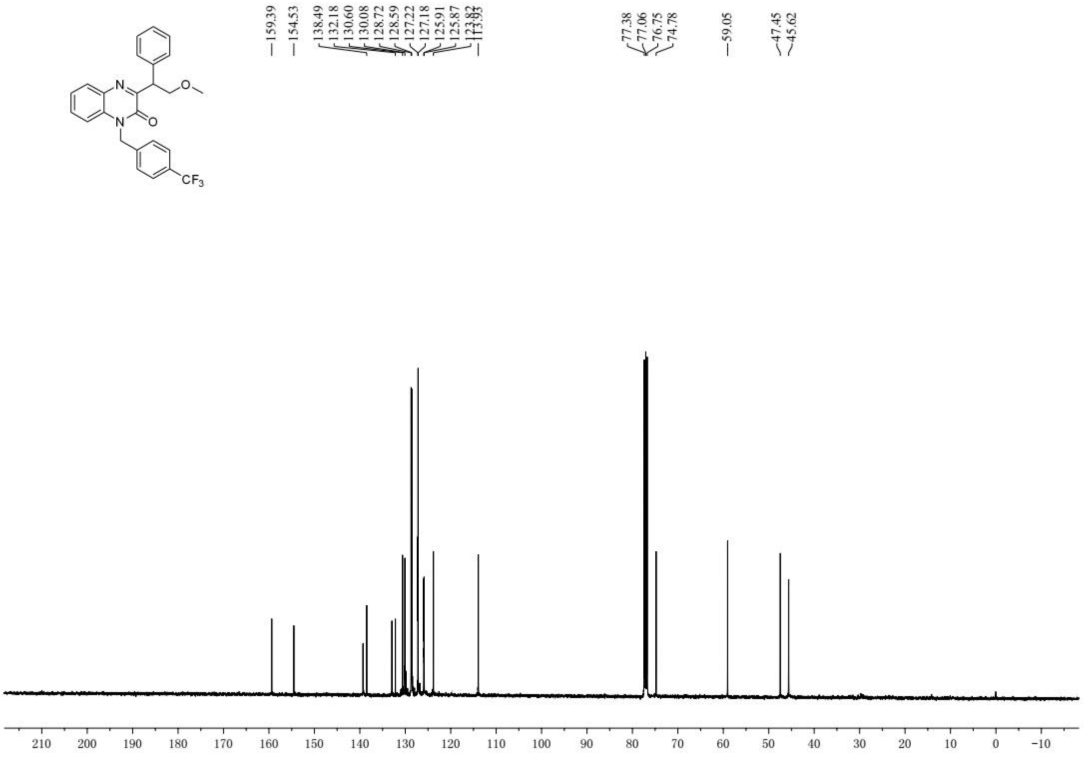

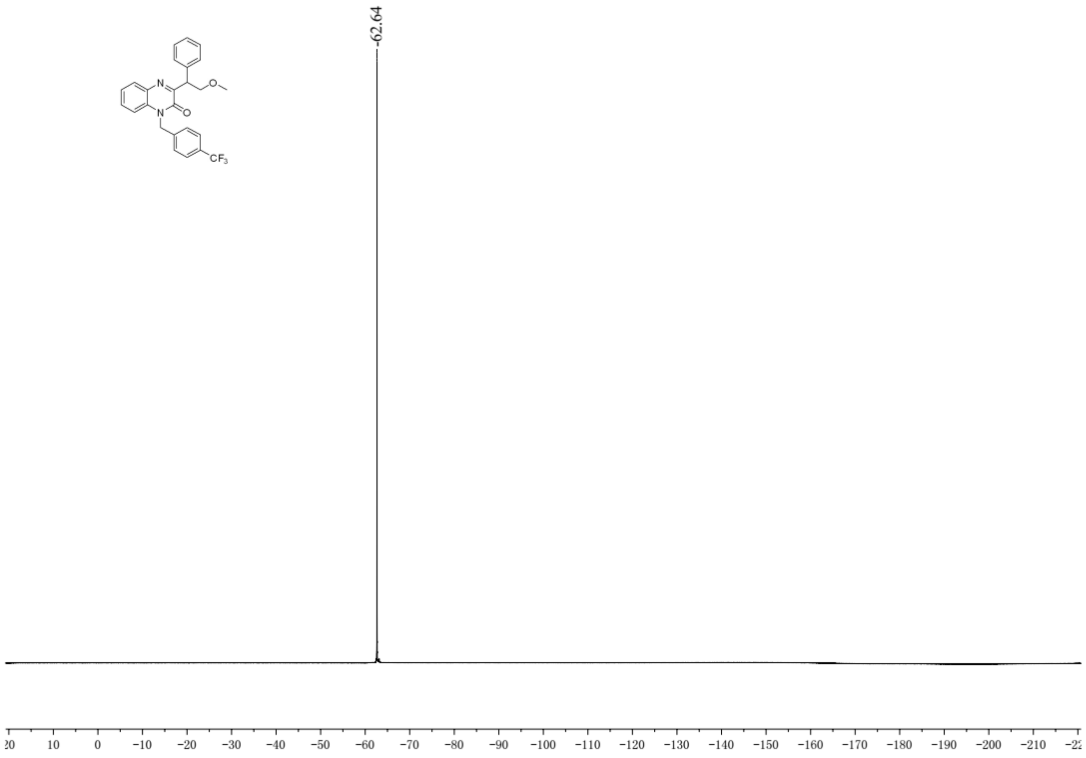
-62.64
N
N
O
O
CF3
10
0
−10
−20
−30
−40
−50
−60
−70
−80
−90
−100
−110
−120
−130
−140
−150
−160
−170
−180
−190
−200
−210

^{1}H and ^{13}C NMR Spectra for **4i**

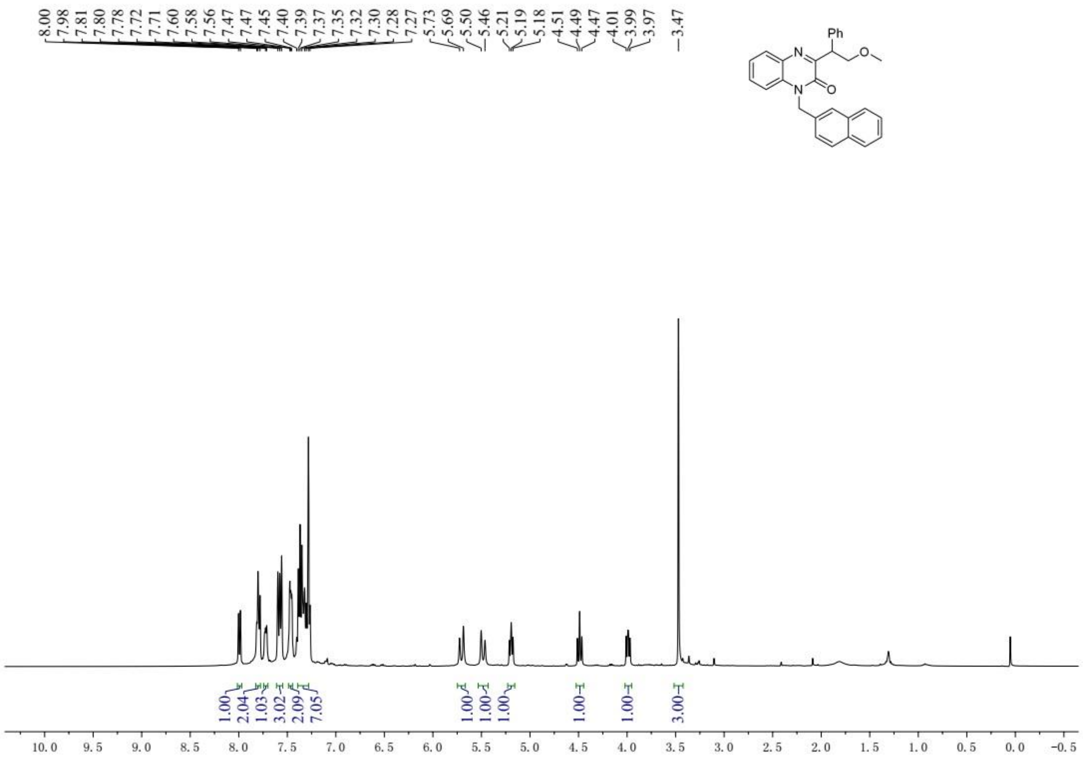

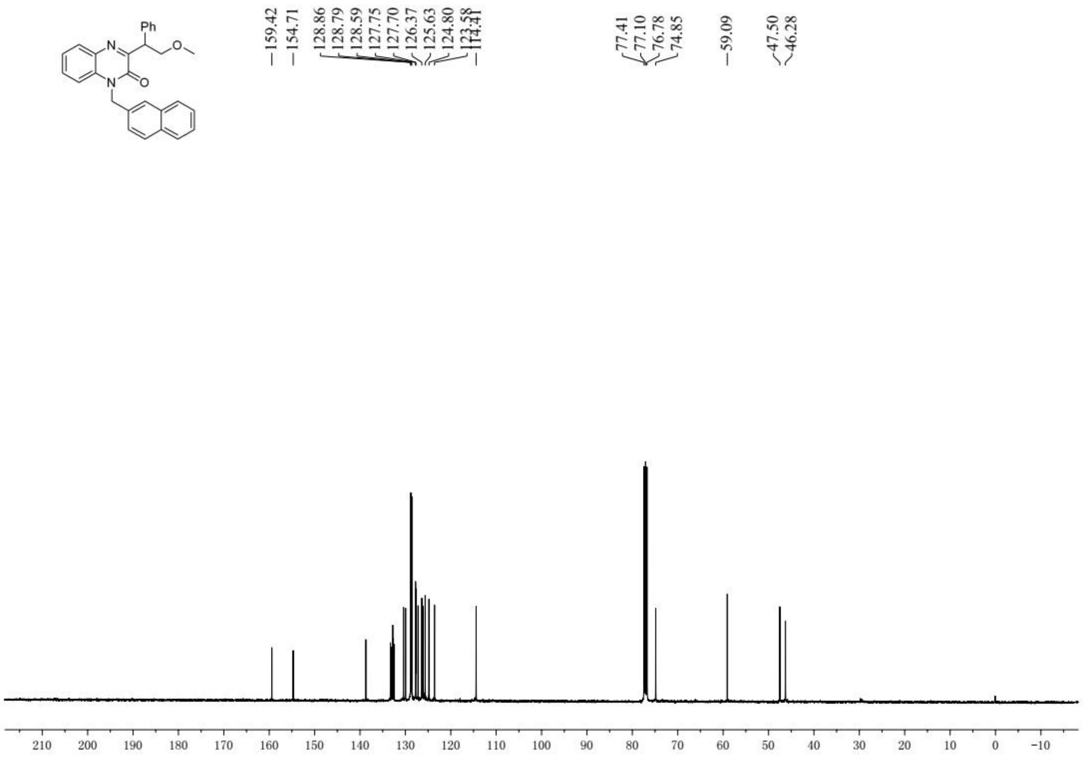

^{1}H and ^{13}C NMR Spectra for **4j**

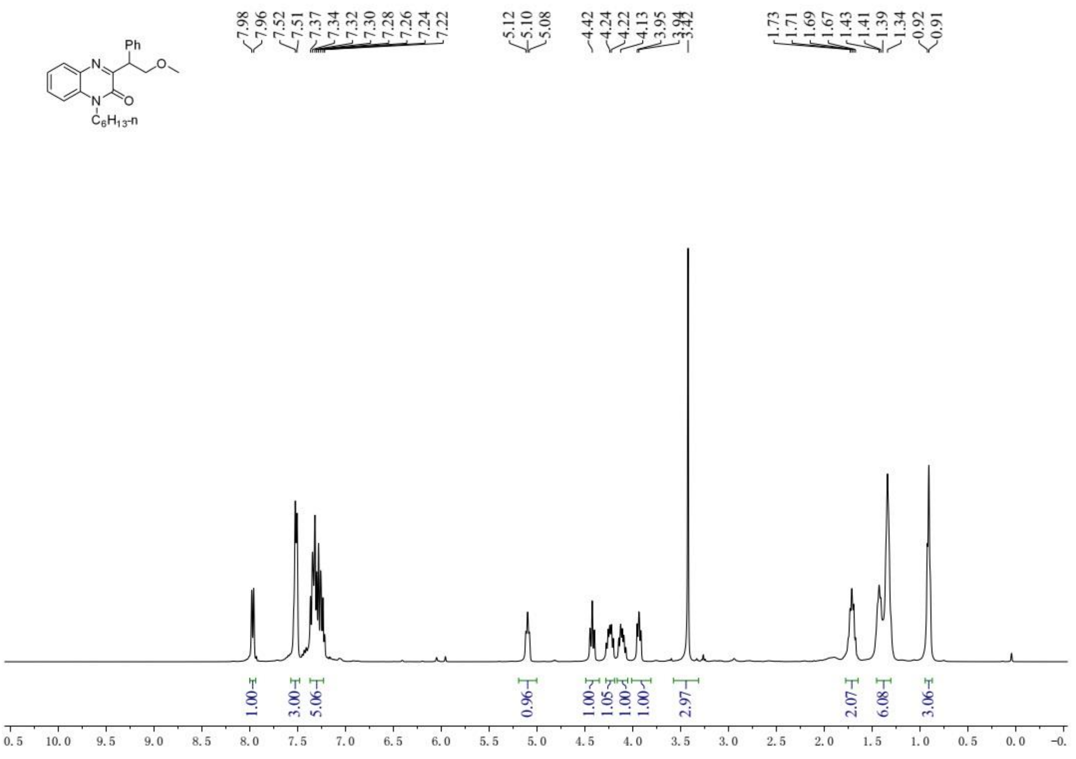

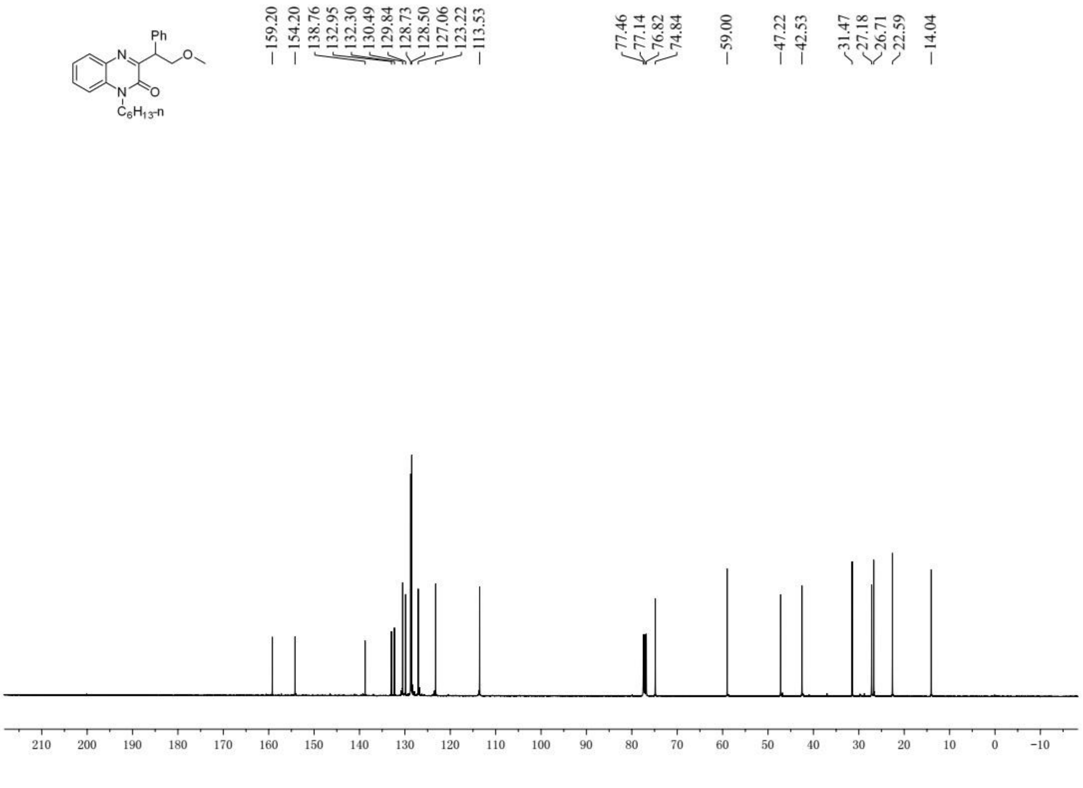

^{1}H and ^{13}C NMR Spectra for **4k**

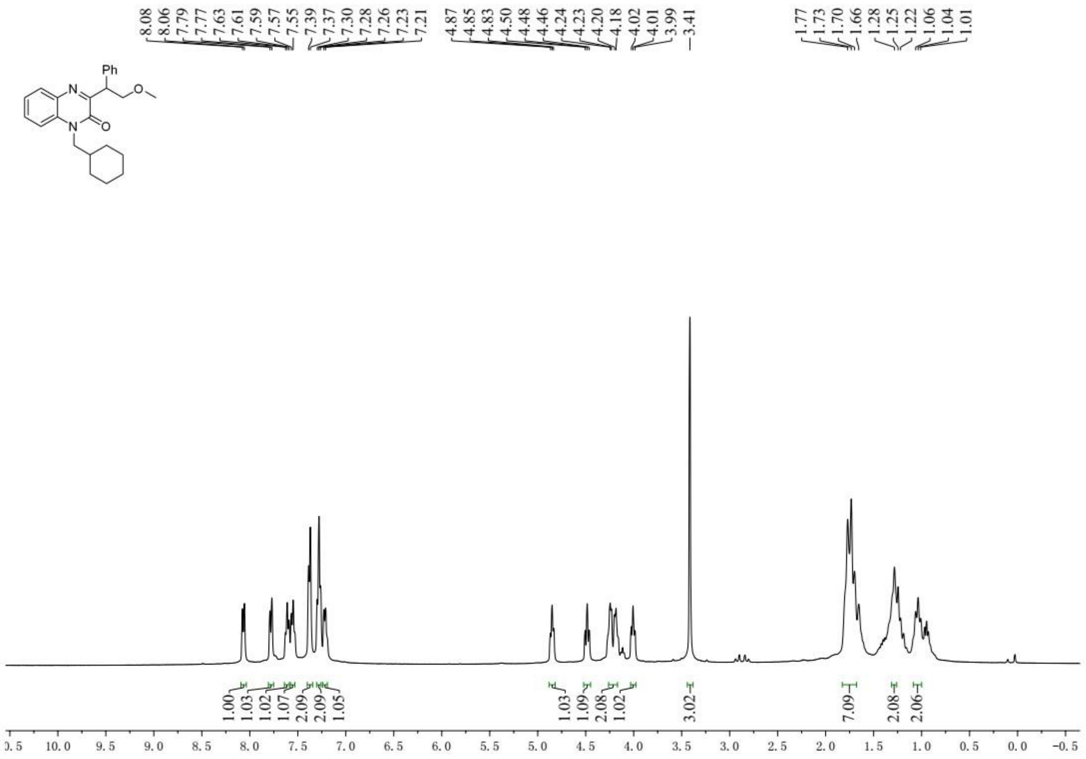

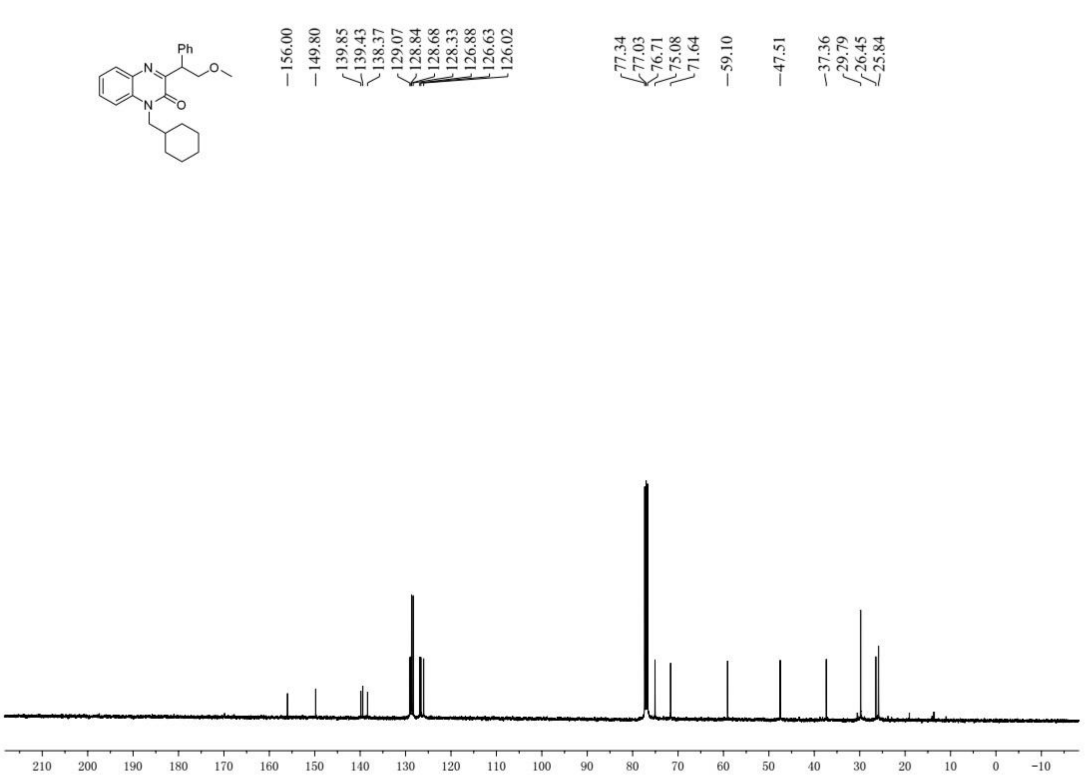

^{1}H and ^{13}C NMR Spectra for **4l**

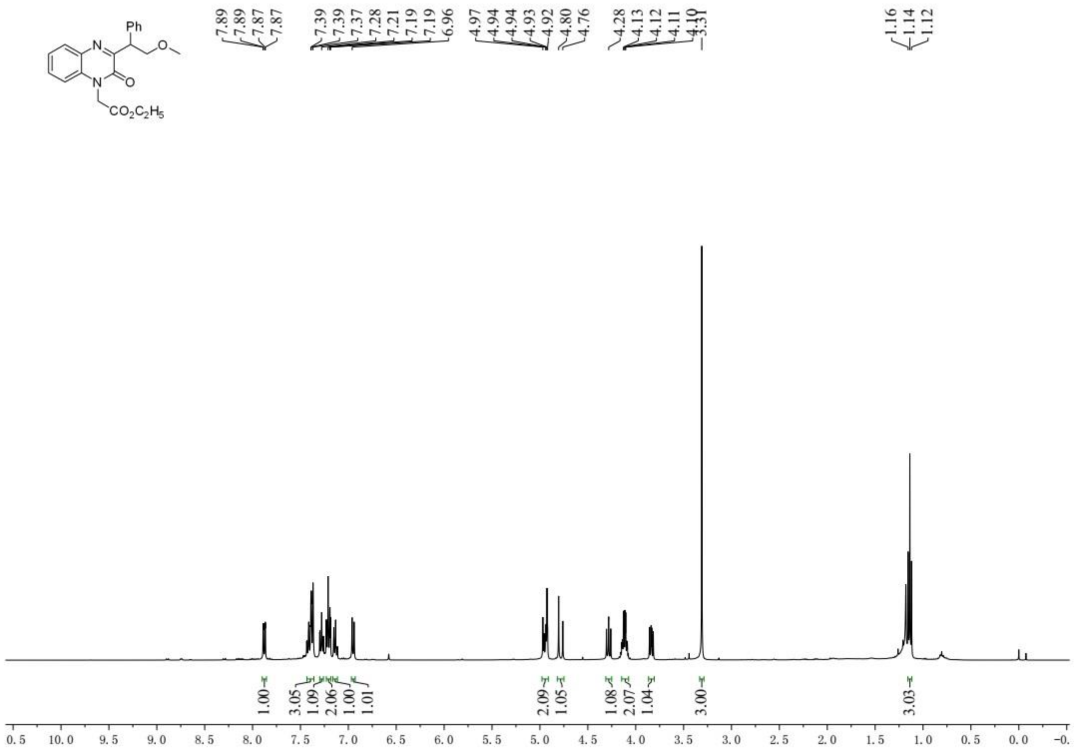

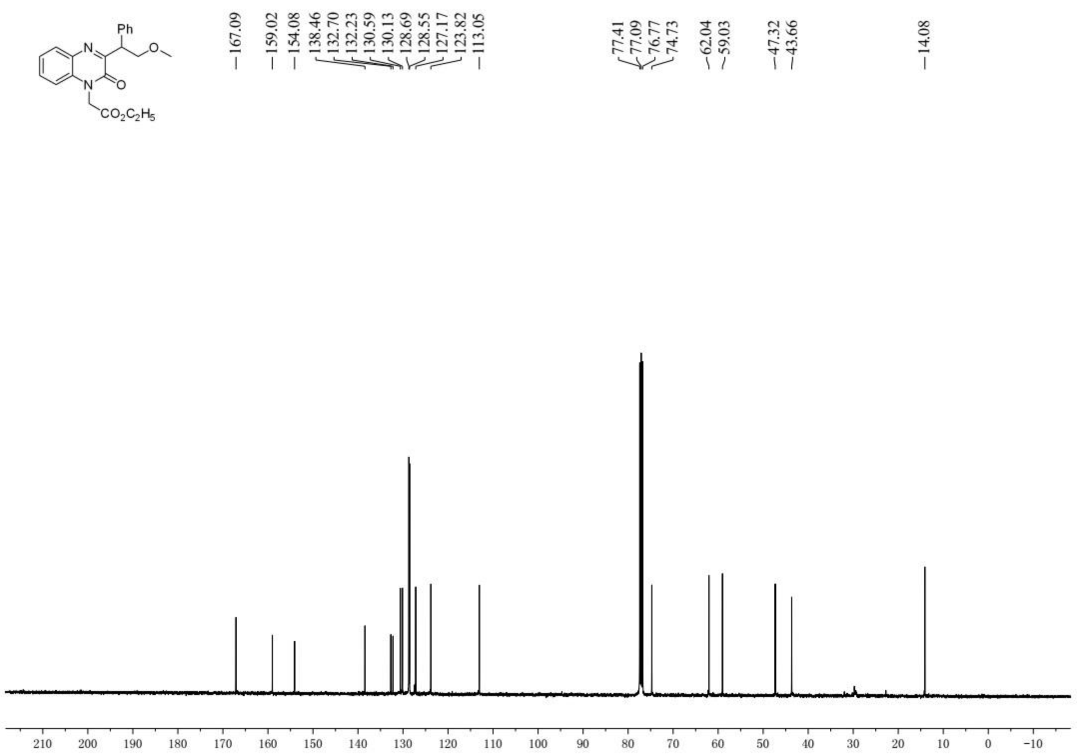

^{1}H and ^{13}C NMR Spectra for **4m**

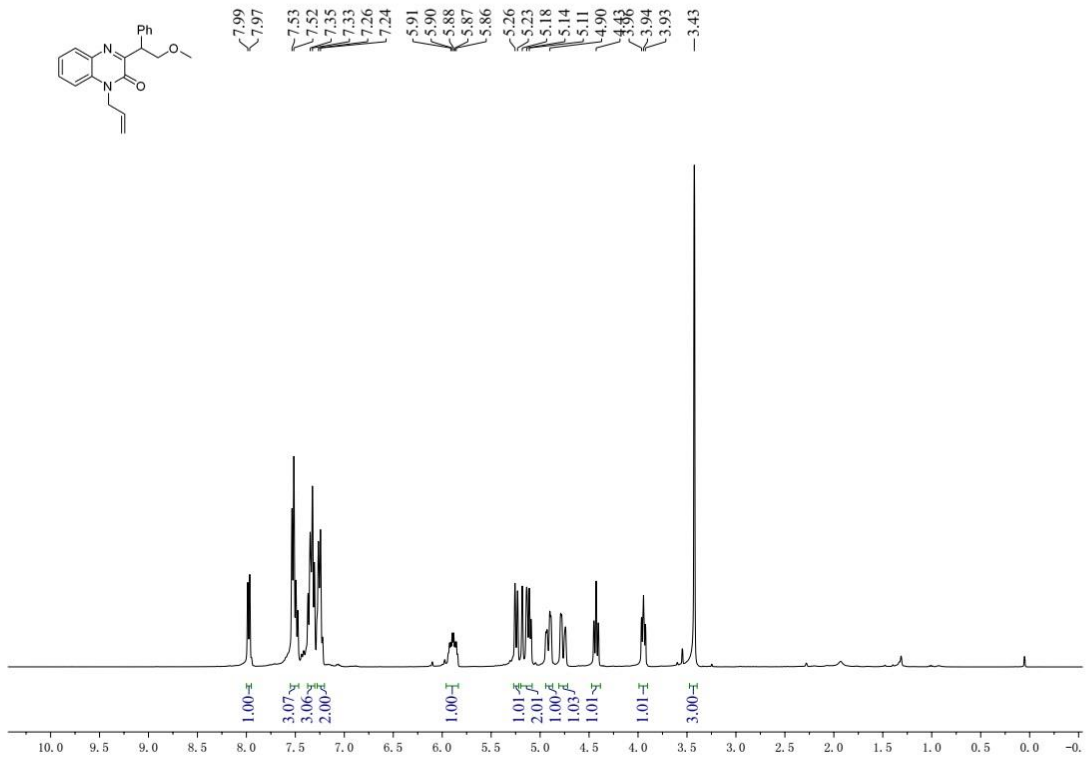

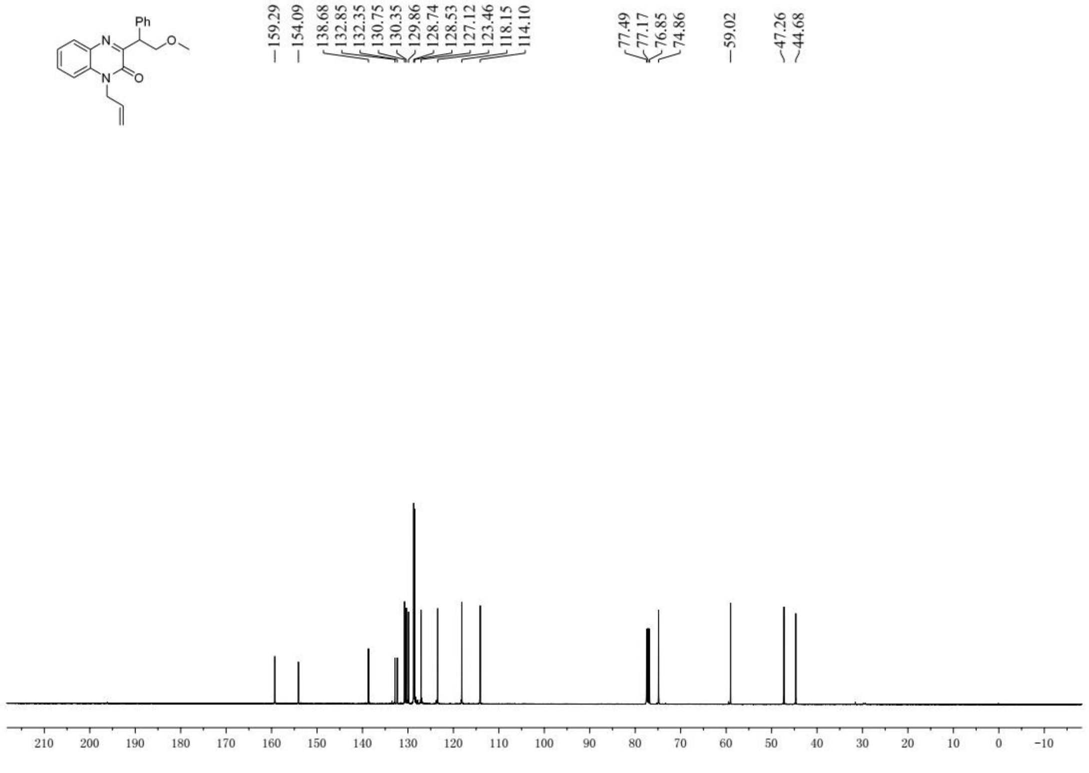

^{1}H and ^{13}C NMR Spectra for **4n**

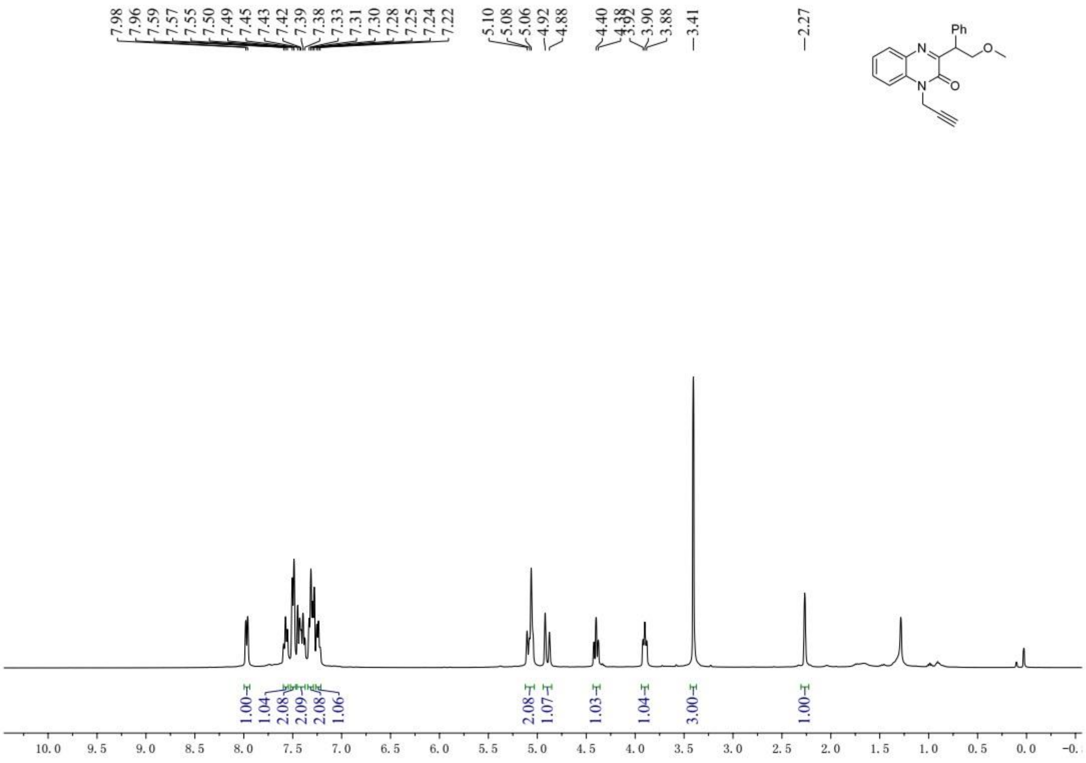

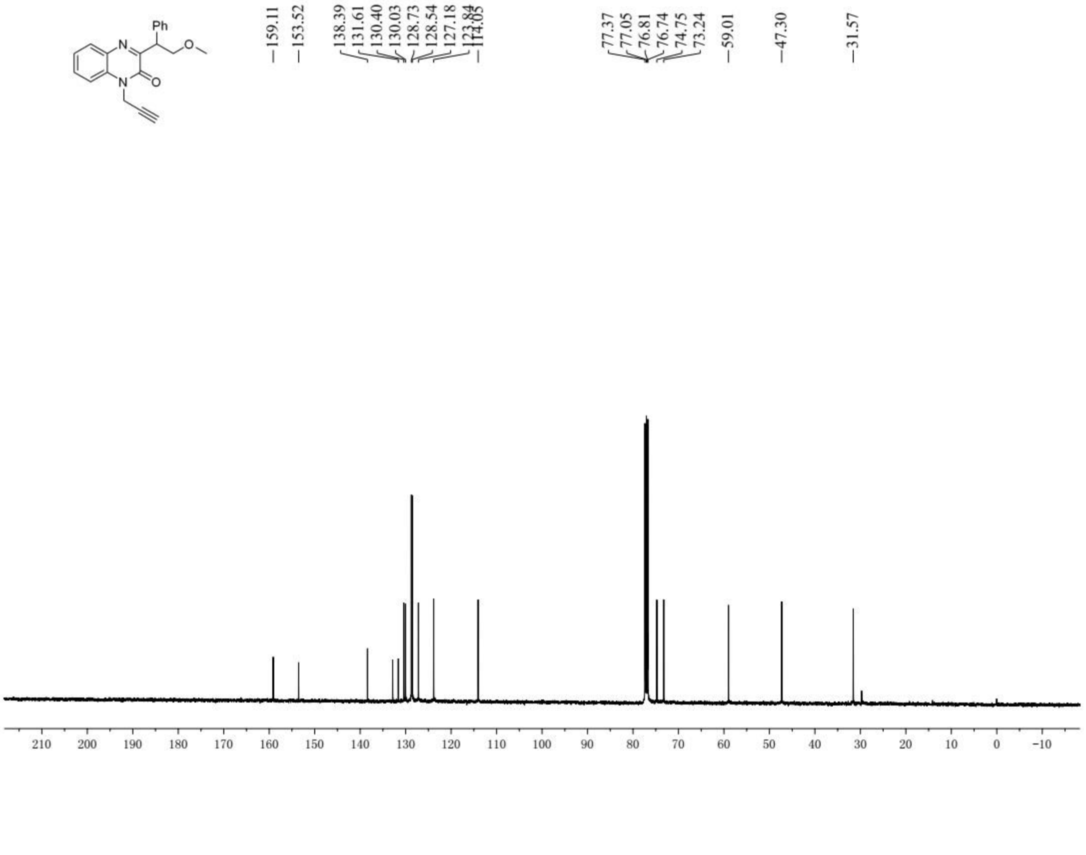

^1H and ^{13}C NMR Spectra for **4o**

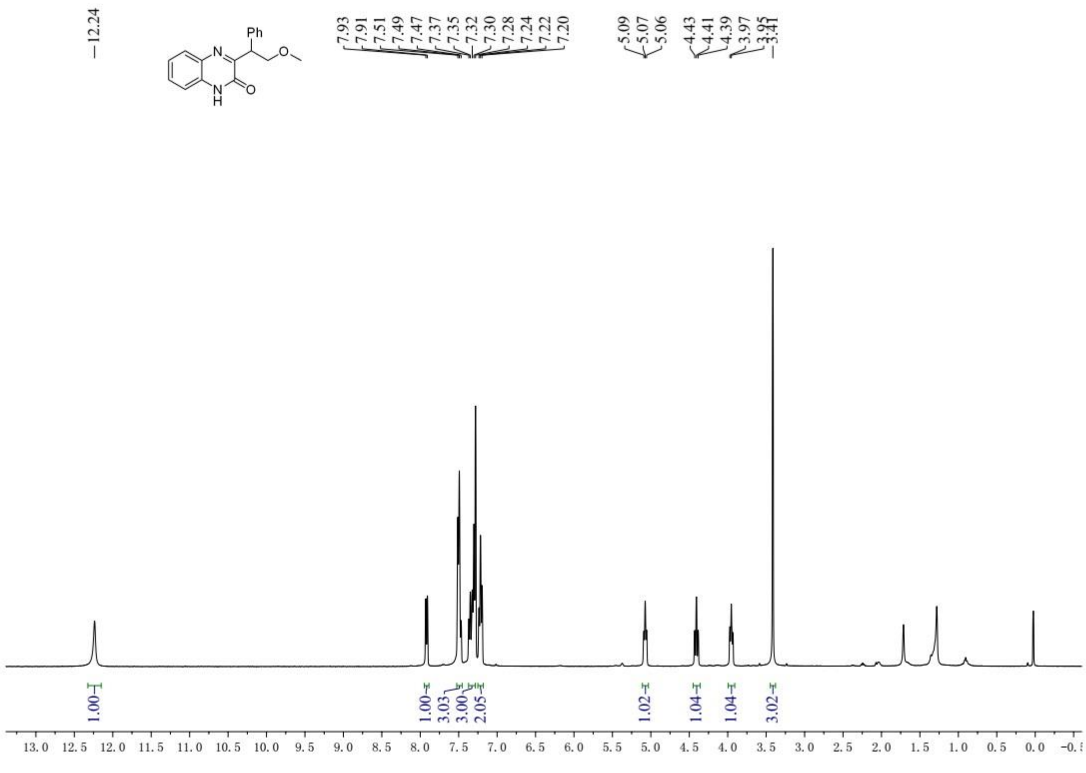

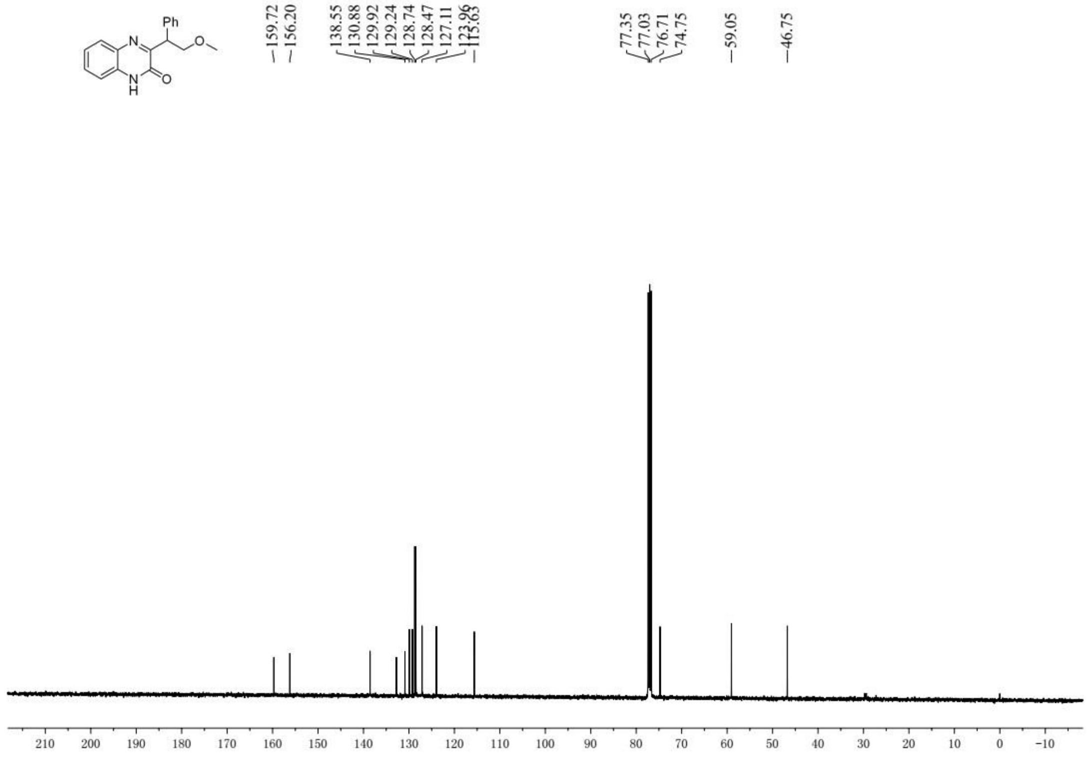

^{1}H and ^{13}C NMR Spectra for **4p**

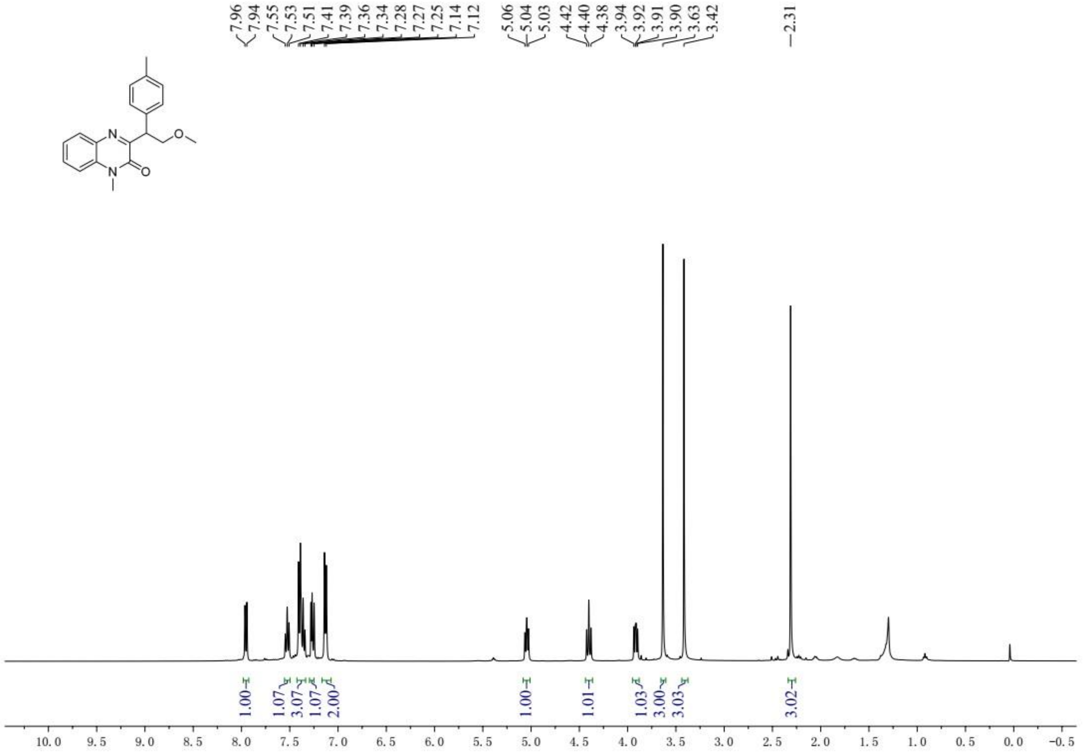

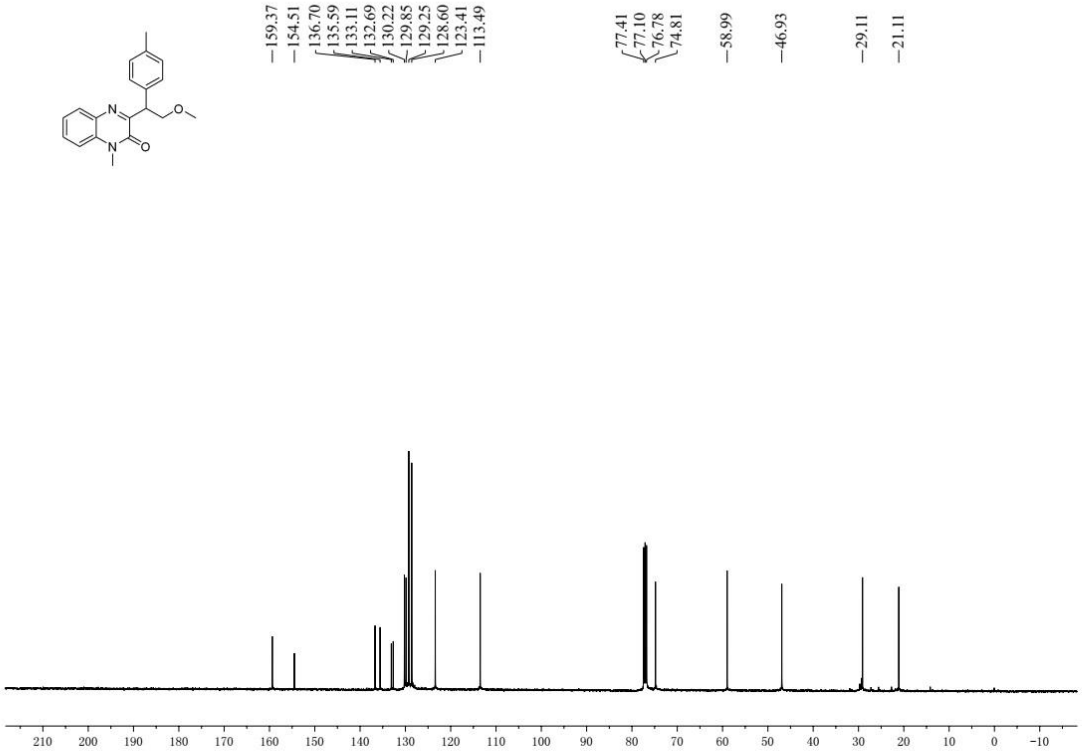

^{1}H and ^{13}C NMR Spectra for **4q**

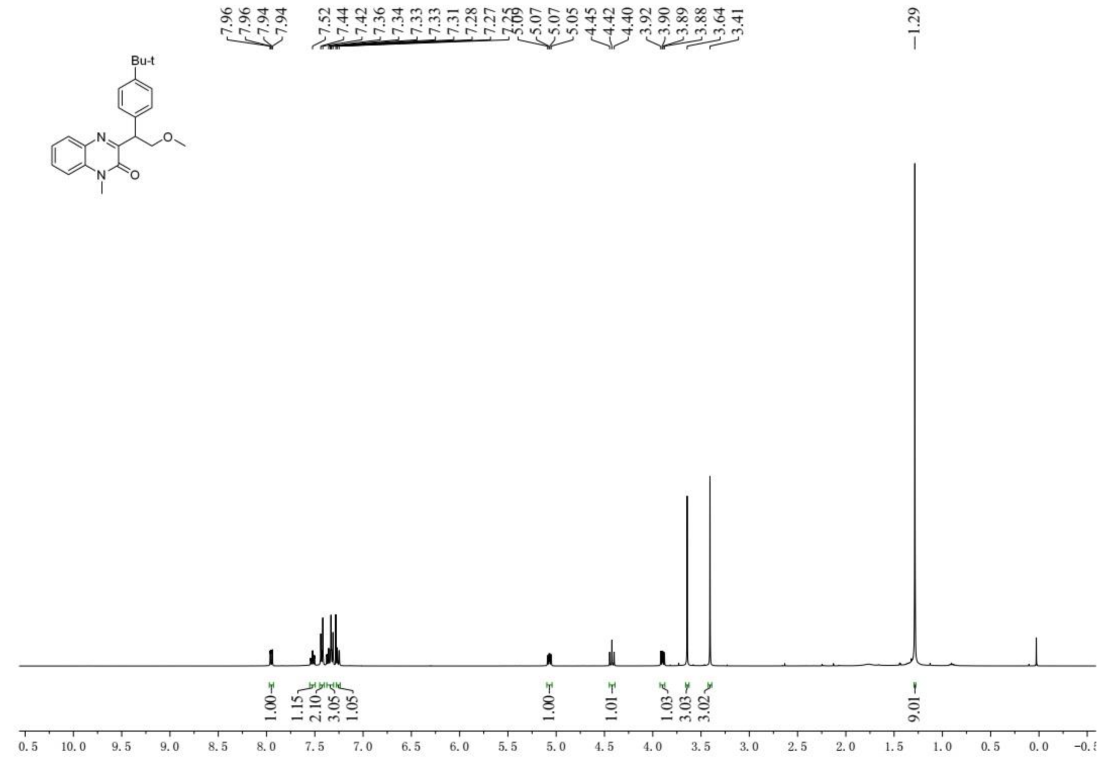

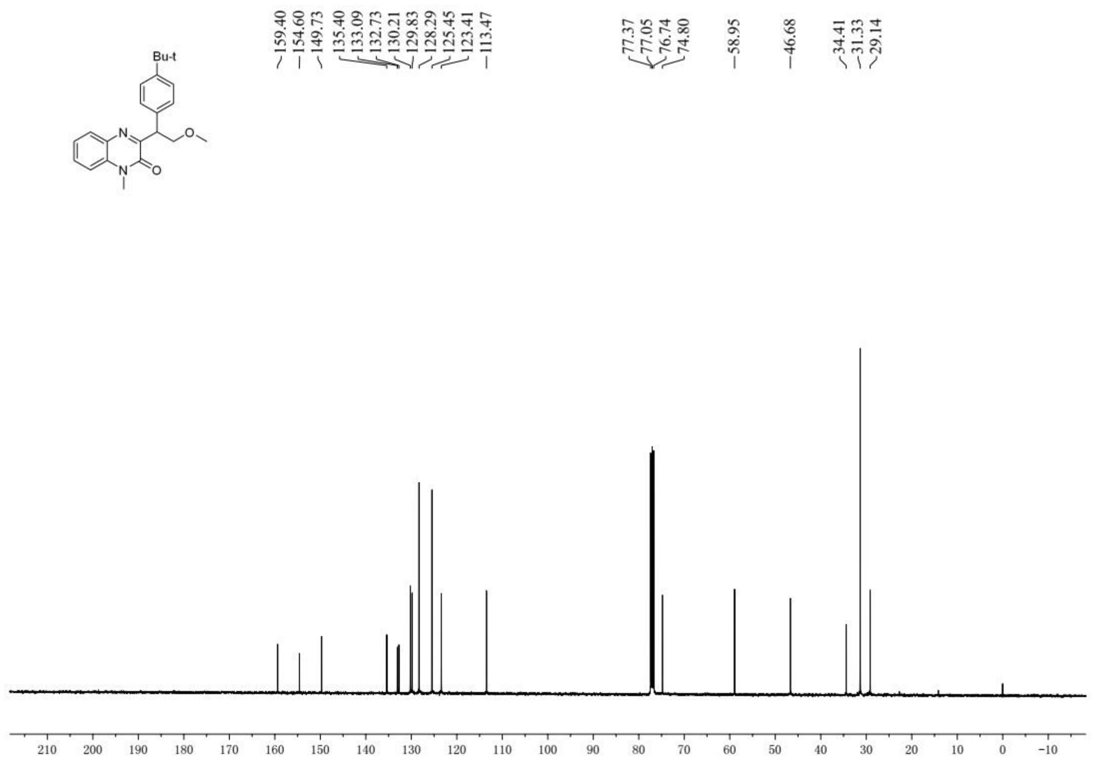

^{1}H and ^{13}C NMR Spectra for **4r**

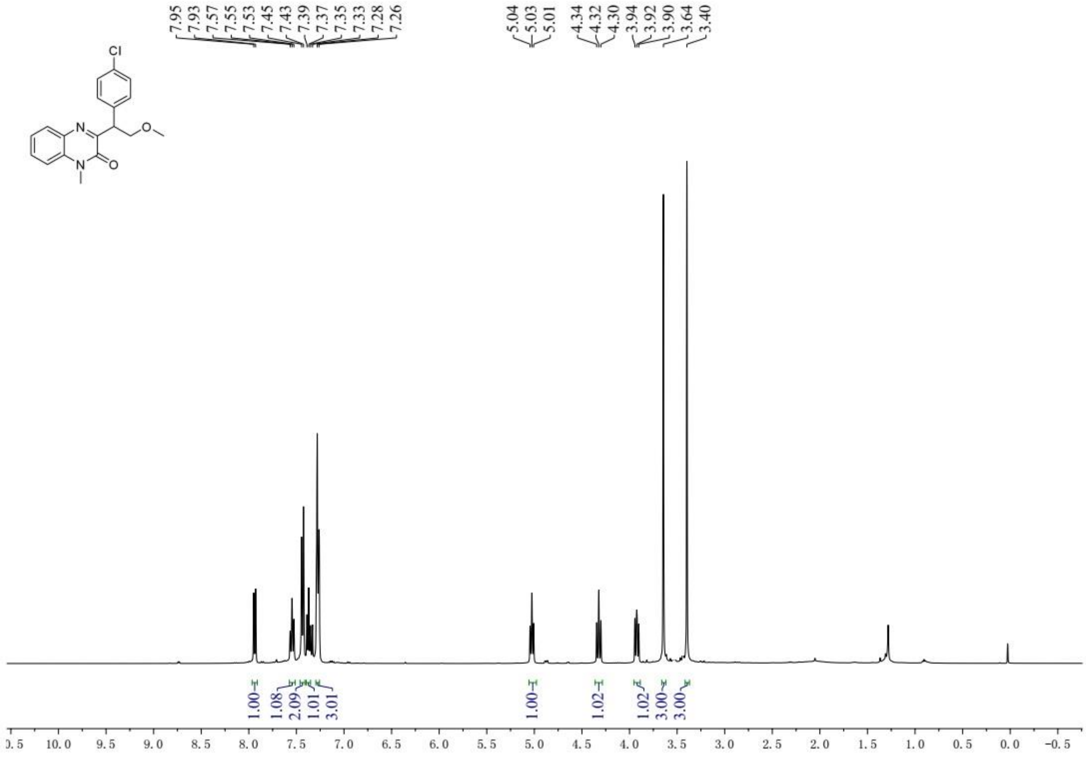

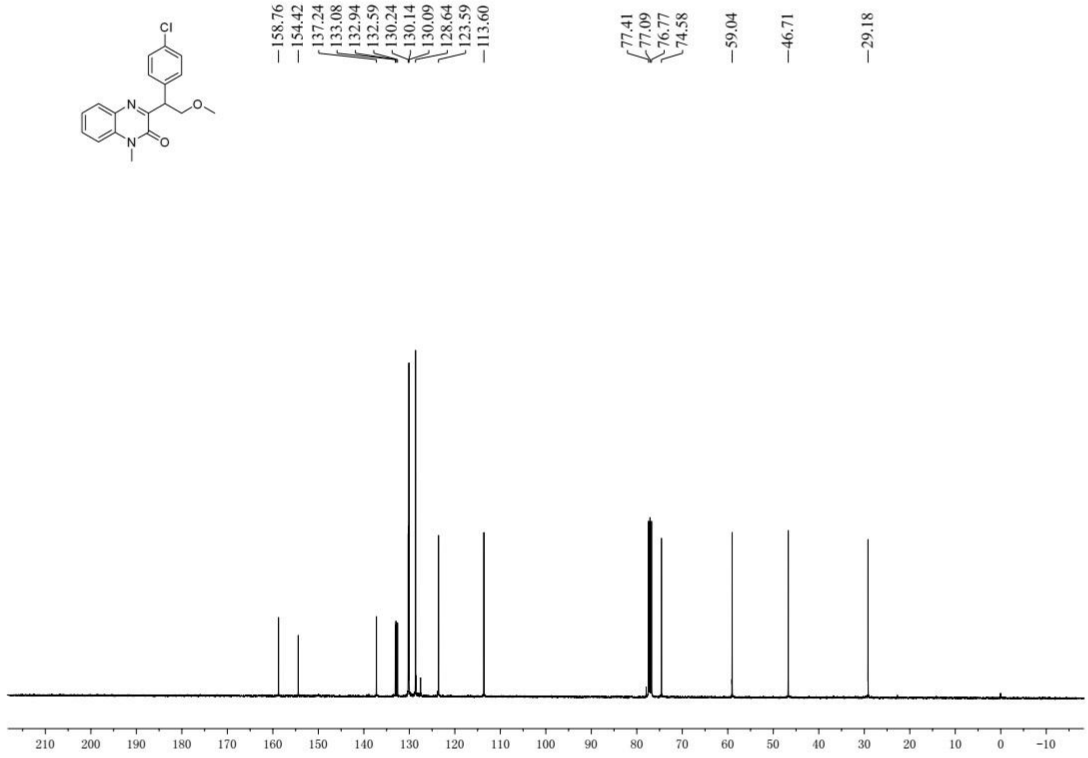

^{1}H and ^{13}C NMR Spectra for **4s**

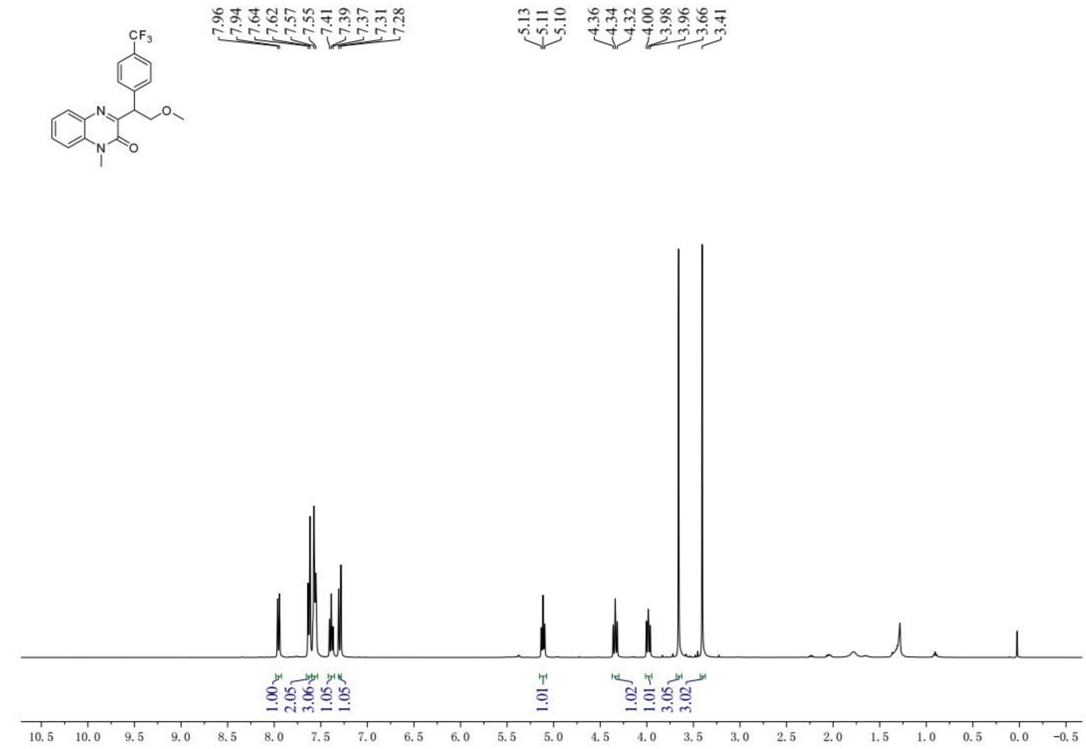

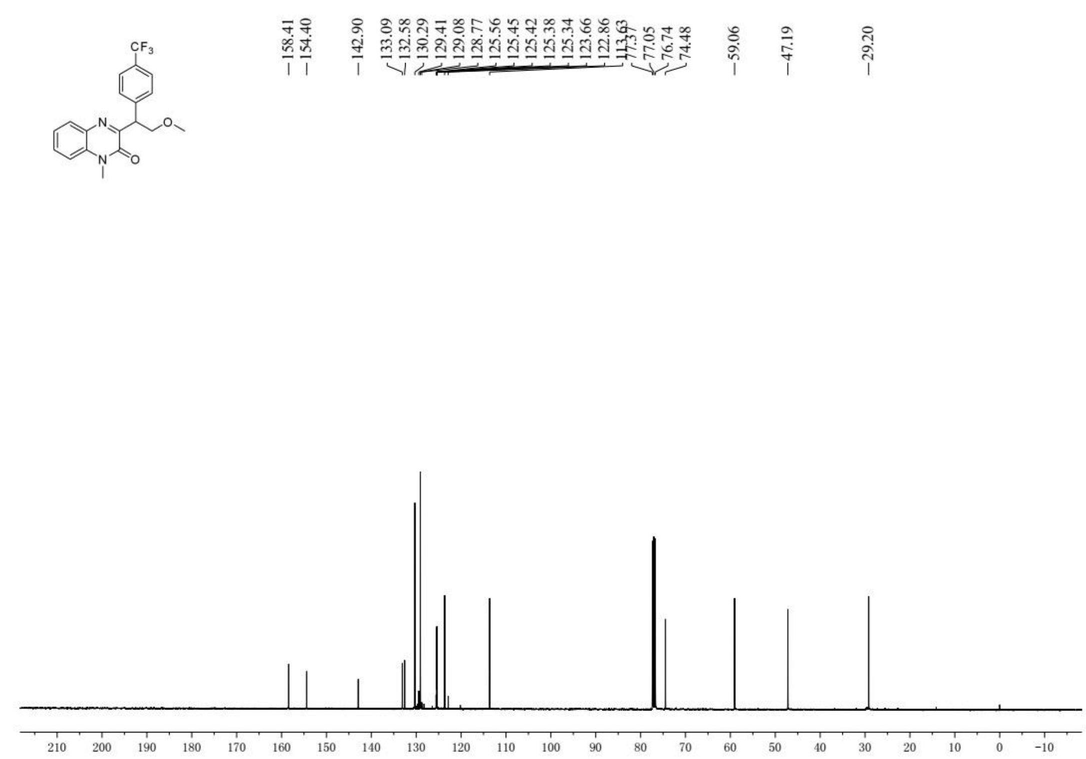

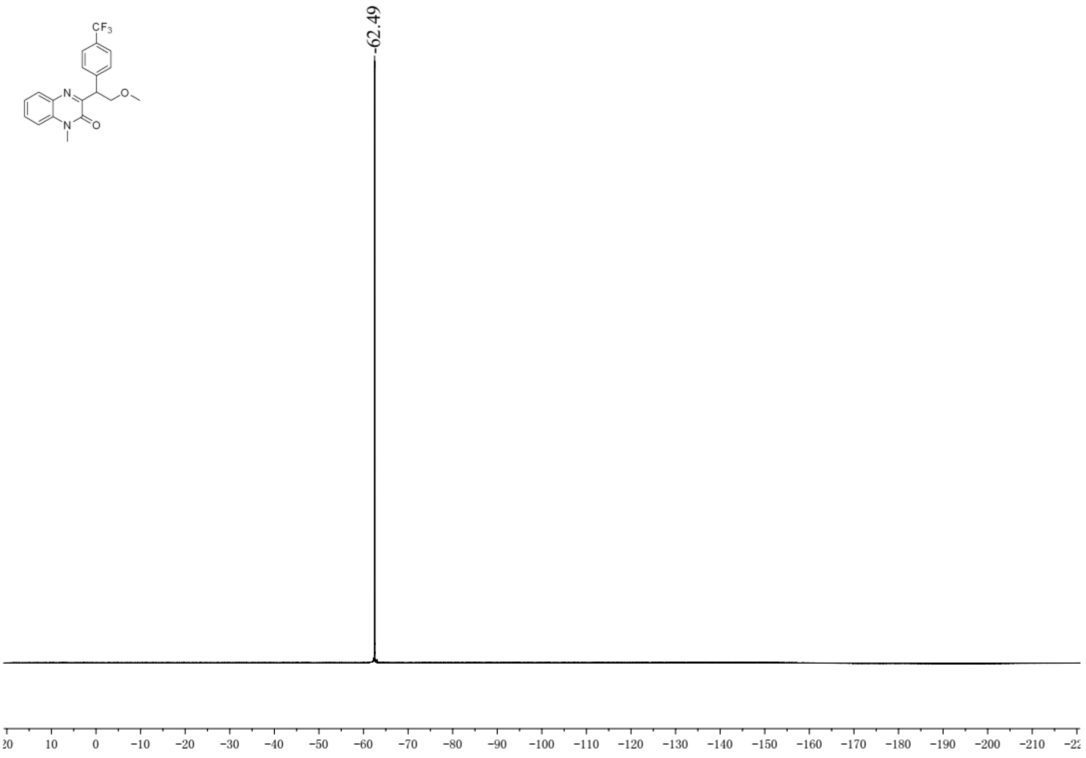

CF3
N
O
-62.49
20
10
0
−10
−20
−30
−40
−50
−60
−70
−80
−90
−100
−110
−120
−130
−140
−150
−160
−170
−180
−190
−200
−210

^{1}H and ^{13}C NMR Spectra for **4t**

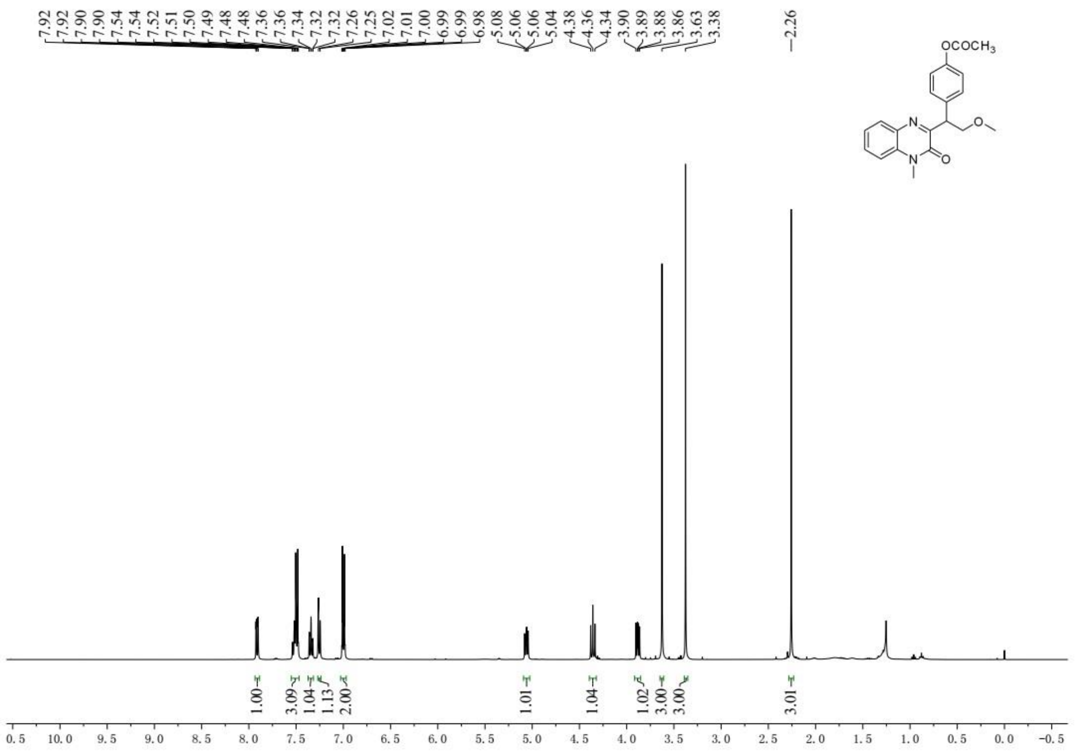

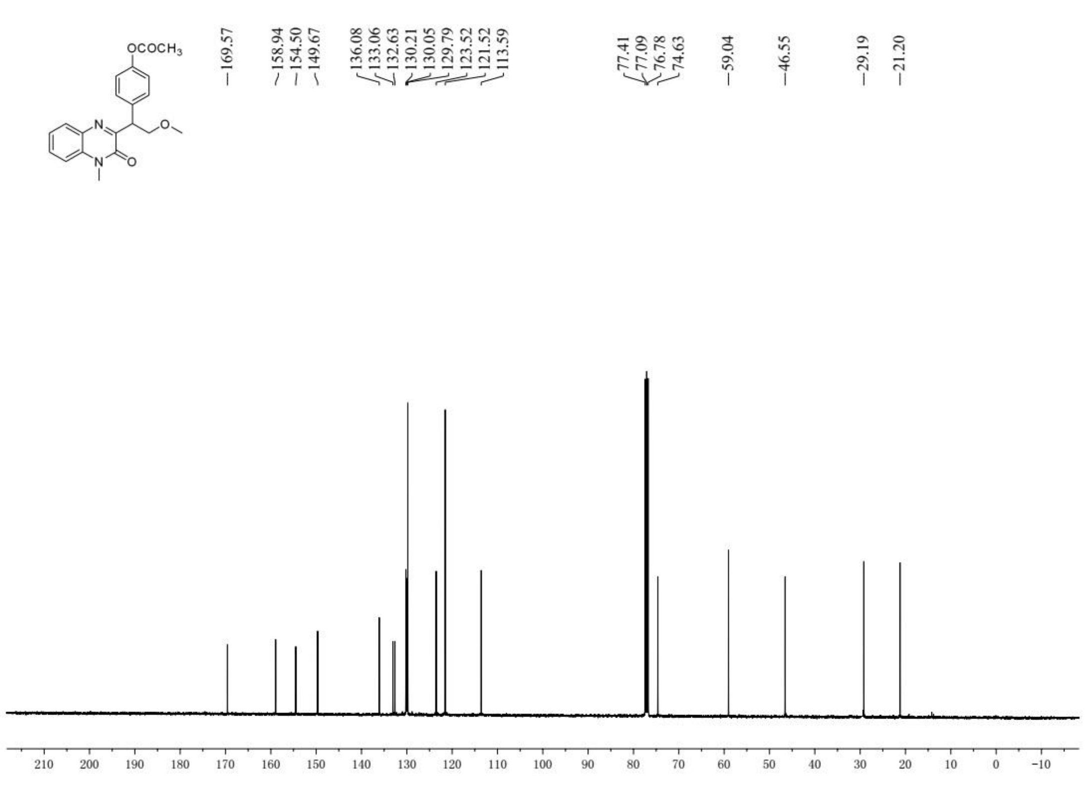

^{1}H and ^{13}C NMR Spectra for **4u**

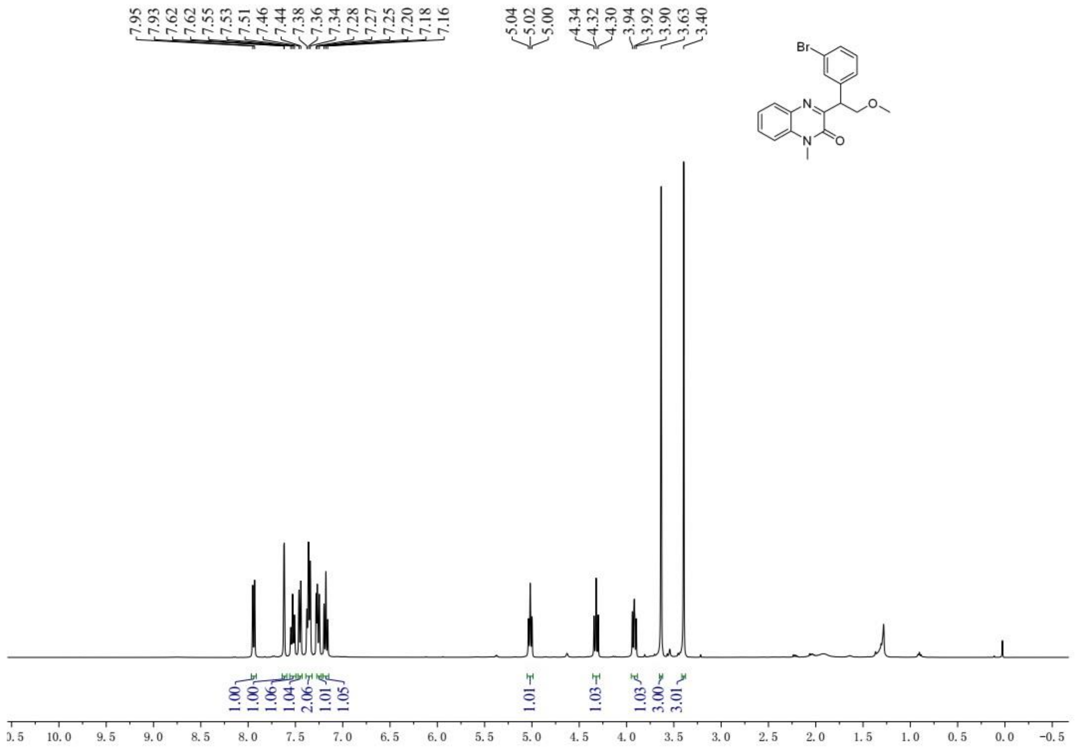

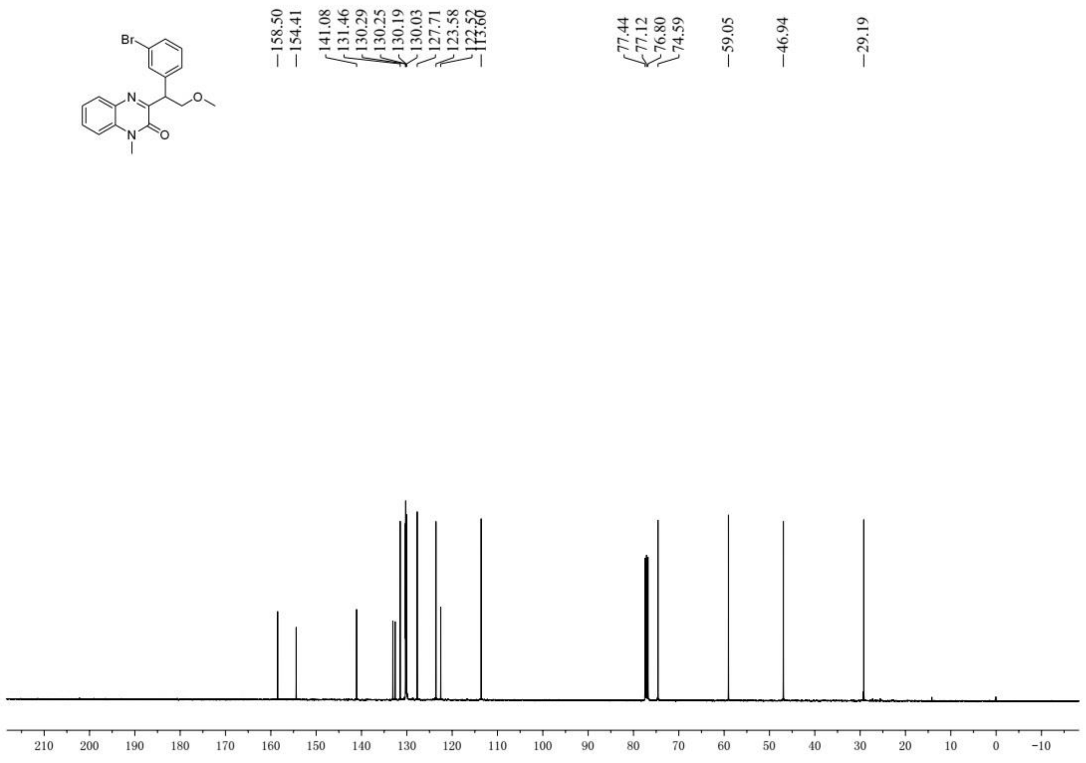

^{1}H and ^{13}C NMR Spectra for **4v**

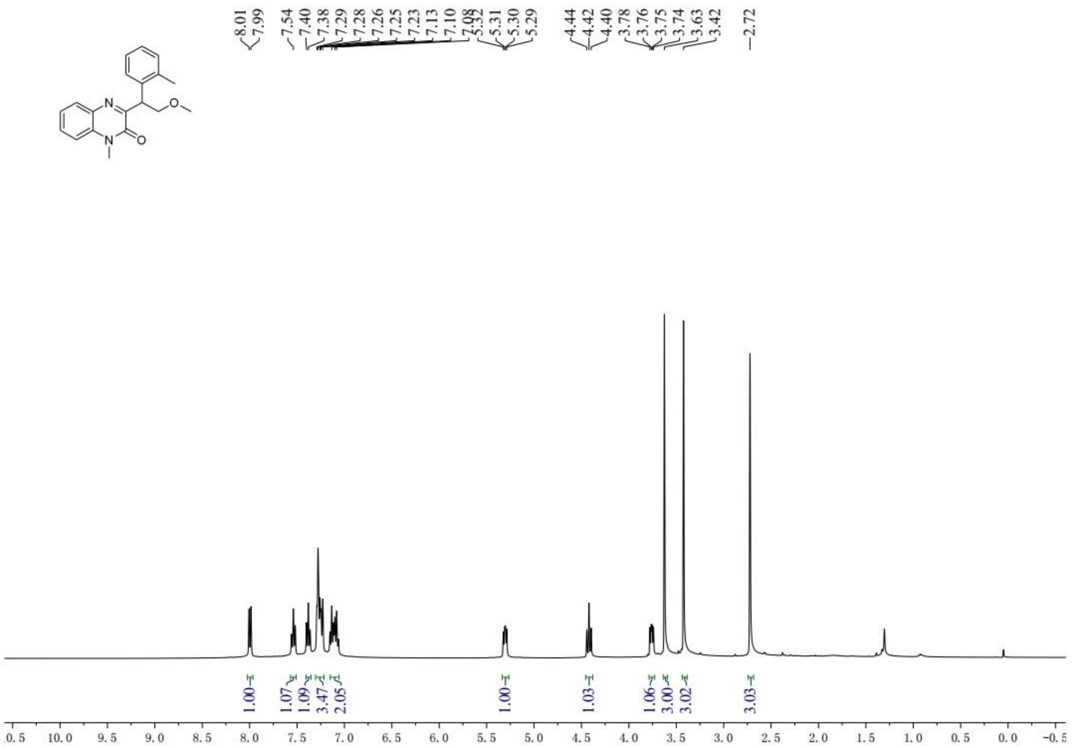

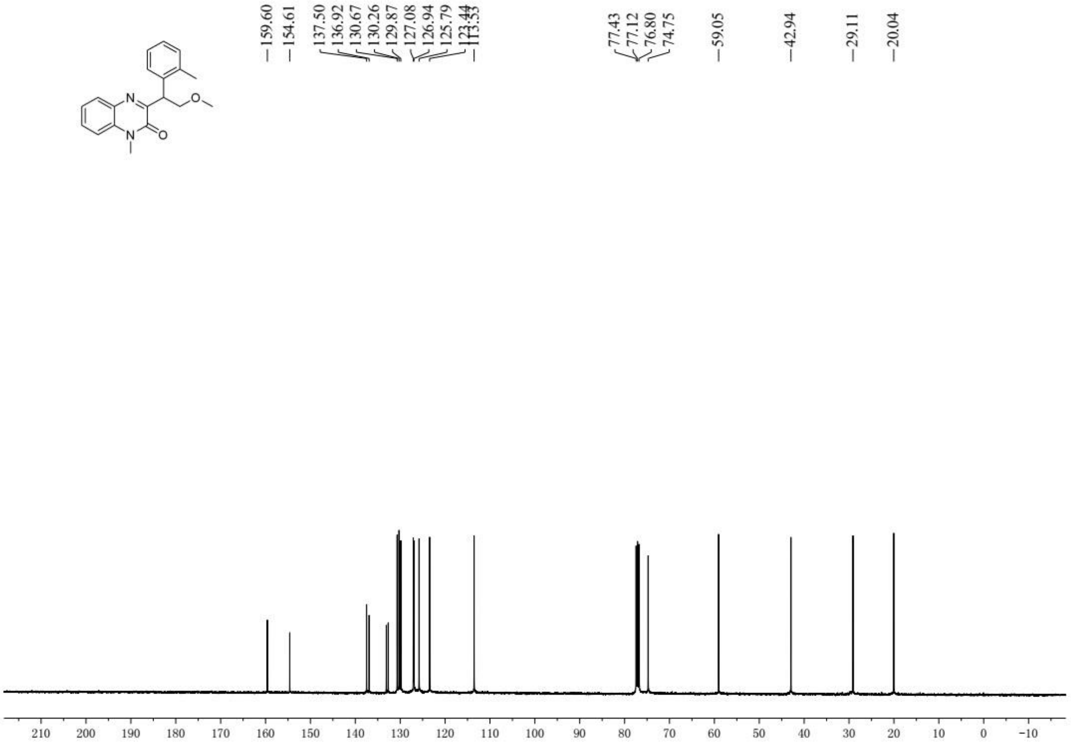

^{1}H and ^{13}C NMR Spectra for **4w**

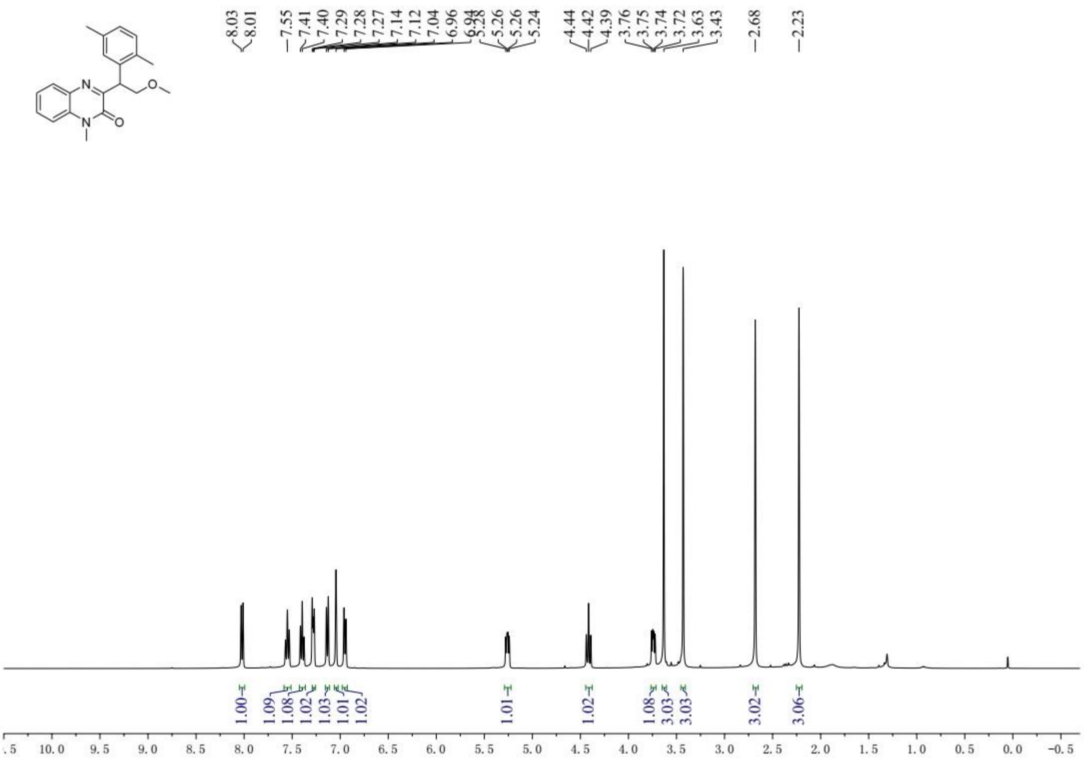

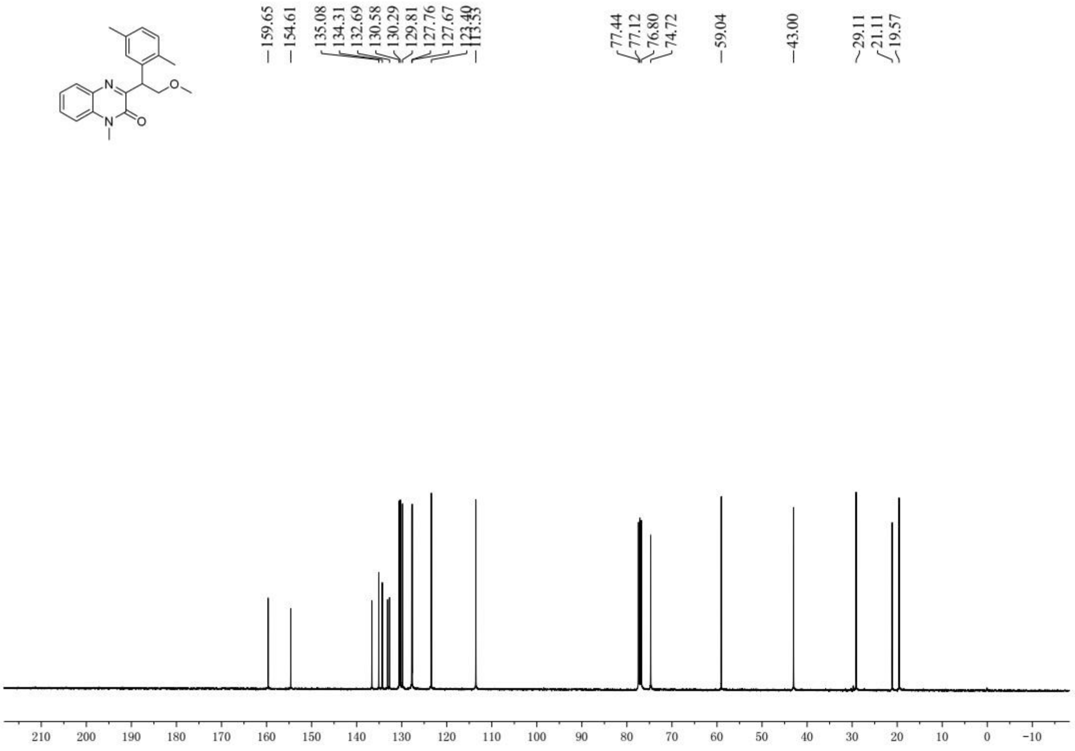

^{1}H and ^{13}C NMR Spectra for **4x**

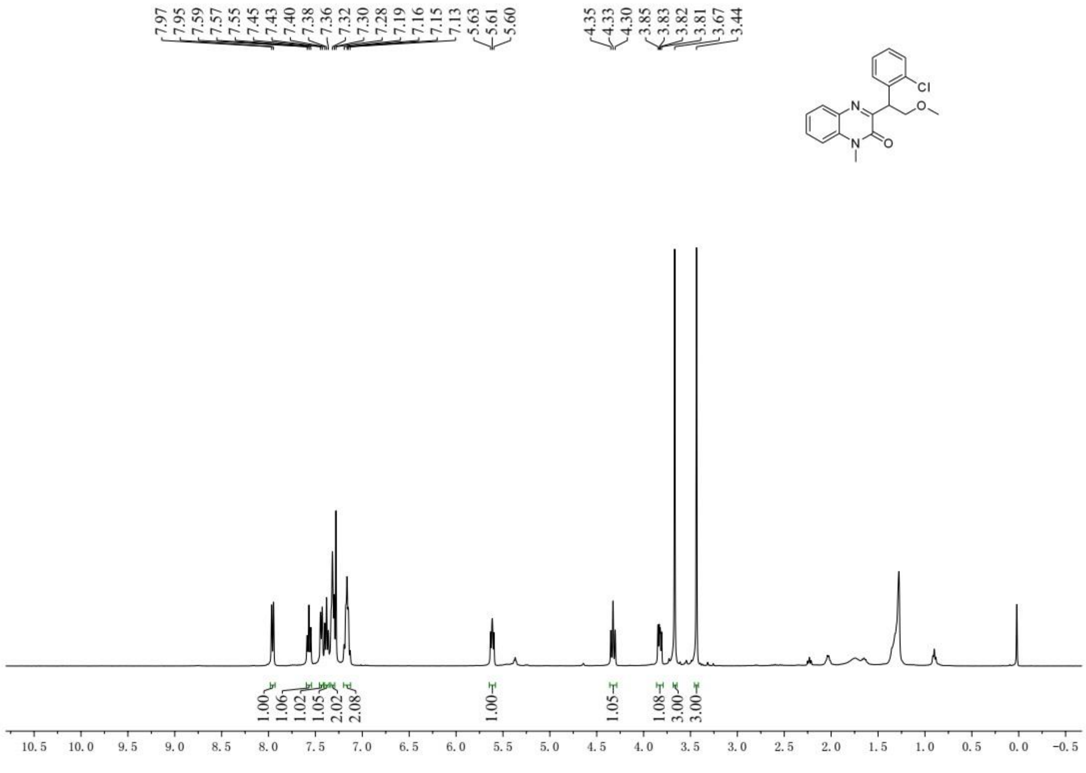

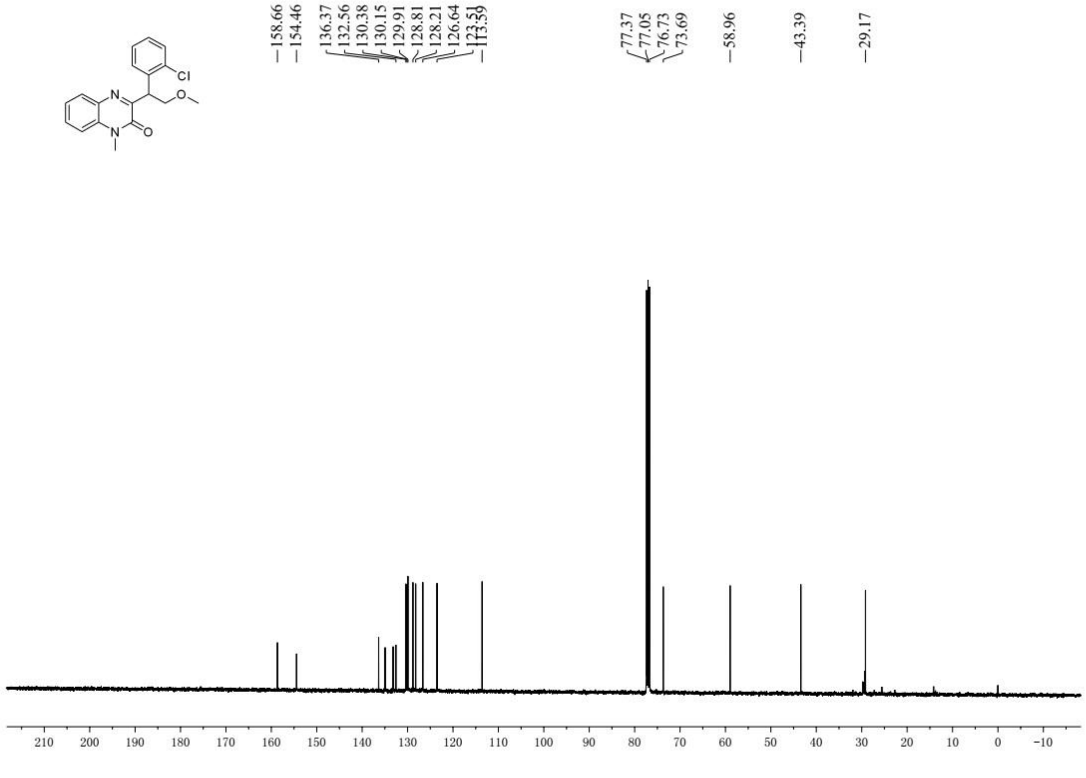

^{1}H and ^{13}C NMR Spectra for **4y**

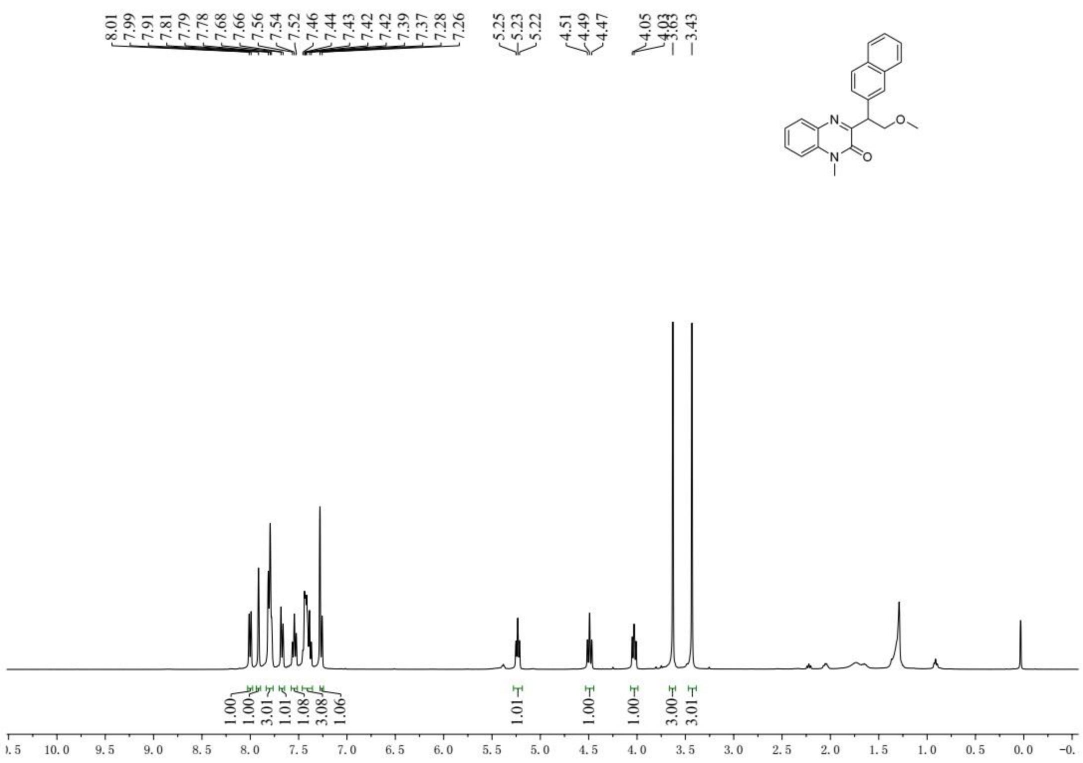

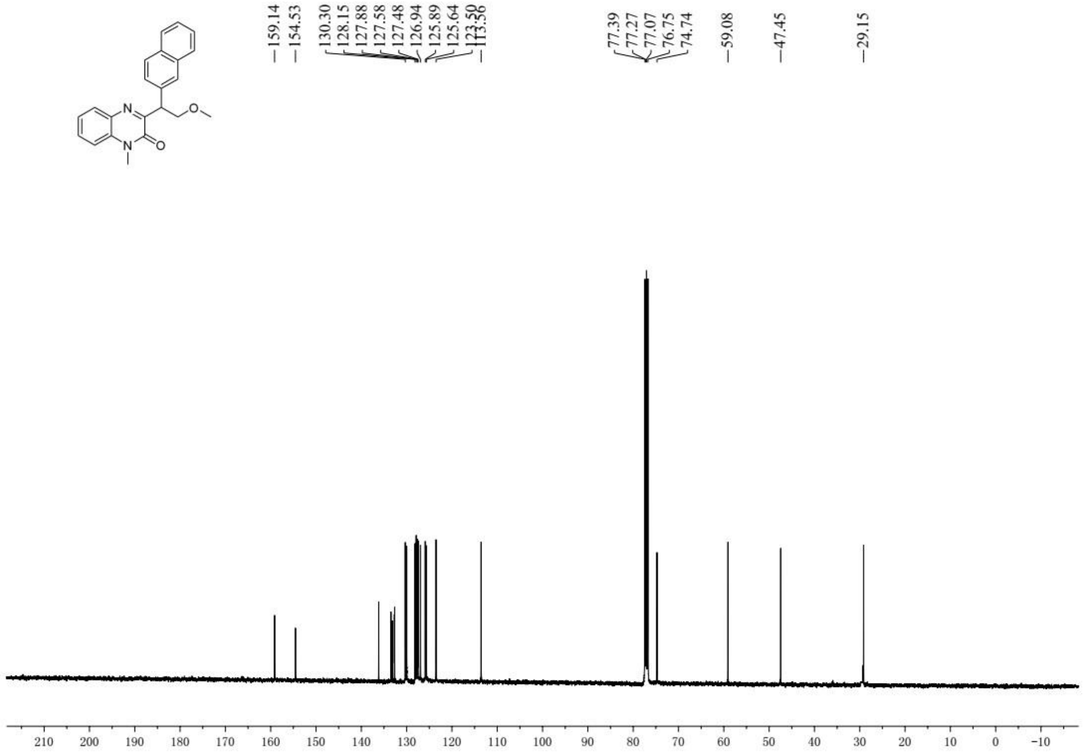

^{1}H and ^{13}C NMR Spectra for **4z**

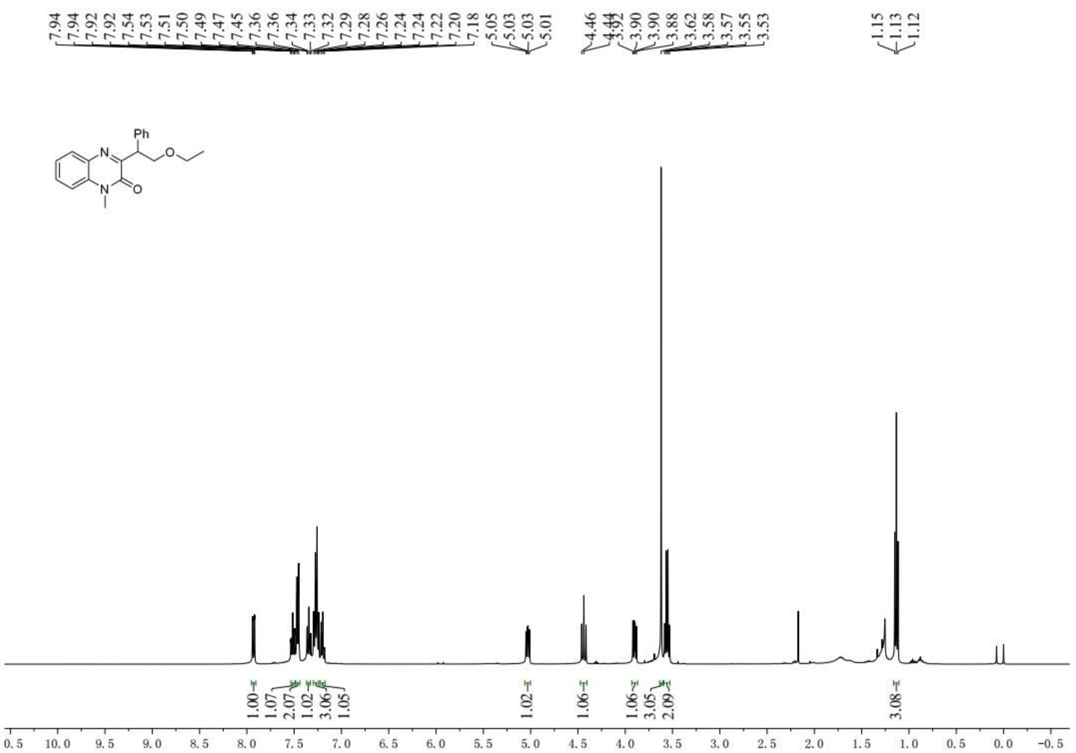

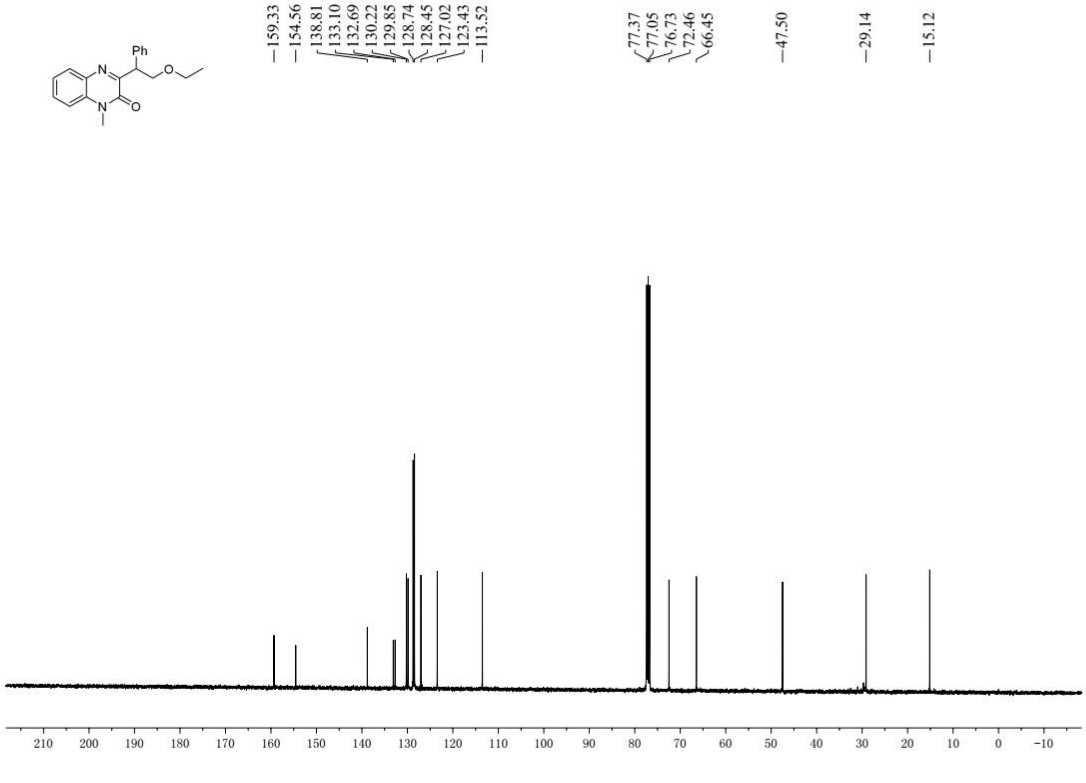

Supplement: Supplementary file 1 [file molecules-27-00475-s001.zip › molecules-1517434-supplementary.pdf]
